# An Overview on the Synthesis of Lamellarins and Related Compounds with Biological Interest

**DOI:** 10.3390/molecules29174032

**Published:** 2024-08-26

**Authors:** Vasiliki-Panagiota M. Mitsiou, Anastasia-Maria N. Antonaki, Matina D. Douka, Konstantinos E. Litinas

**Affiliations:** Laboratory of Organic Chemistry, Department of Chemistry, Aristotle University of Thessaloniki, 54124 Thessaloniki, Greece; vmitsiou@chem.auth.gr (V.-P.M.M.); anastasna@chem.auth.gr (A.-M.N.A.); doukamatina@chem.auth.gr (M.D.D.)

**Keywords:** lamellarins, azacoumestans, isolamellarins, isoazacoumestans, isoquinoline, coumarins, cytotoxic activity, anti-HIV activity, anti-inflammatory activity

## Abstract

Lamellarins are natural products with a [3,4]-fused pyrrolocoumarin skeleton possessing interesting biological properties. More than 70 members have been isolated from diverse marine organisms, such as sponges, ascidians, mollusks, and tunicates. There is a continuous interest in the synthesis of these compounds. In this review, the synthetic strategies for the synthesis of the title compounds are presented along with their biological properties. Three routes are followed for the synthesis of lamellarins. Initially, pyrrole derivatives are the starting or intermediate compounds, and then they are fused to isoquinoline or a coumarin moiety. Second, isoquinoline is the starting compound fused to an indole moiety. In the last route, coumarins are the starting compounds, which are fused to a pyrrole moiety and an isoquinoline scaffold. The synthesis of isolamellarins, azacoumestans, isoazacoumestans, and analogues is also described. The above synthesis is achieved via metal-catalyzed cross-coupling, [3 + 2] cycloaddition, substitution, and lactonization reactions. The title compounds exhibit cytotoxic, multidrug resistance (MDR), topoisomerase I-targeted antitumor, anti-HIV, antiproliferative, anti-neurodegenerative disease, and anti-inflammatory activities.

## 1. Introduction

**Figure 1:**
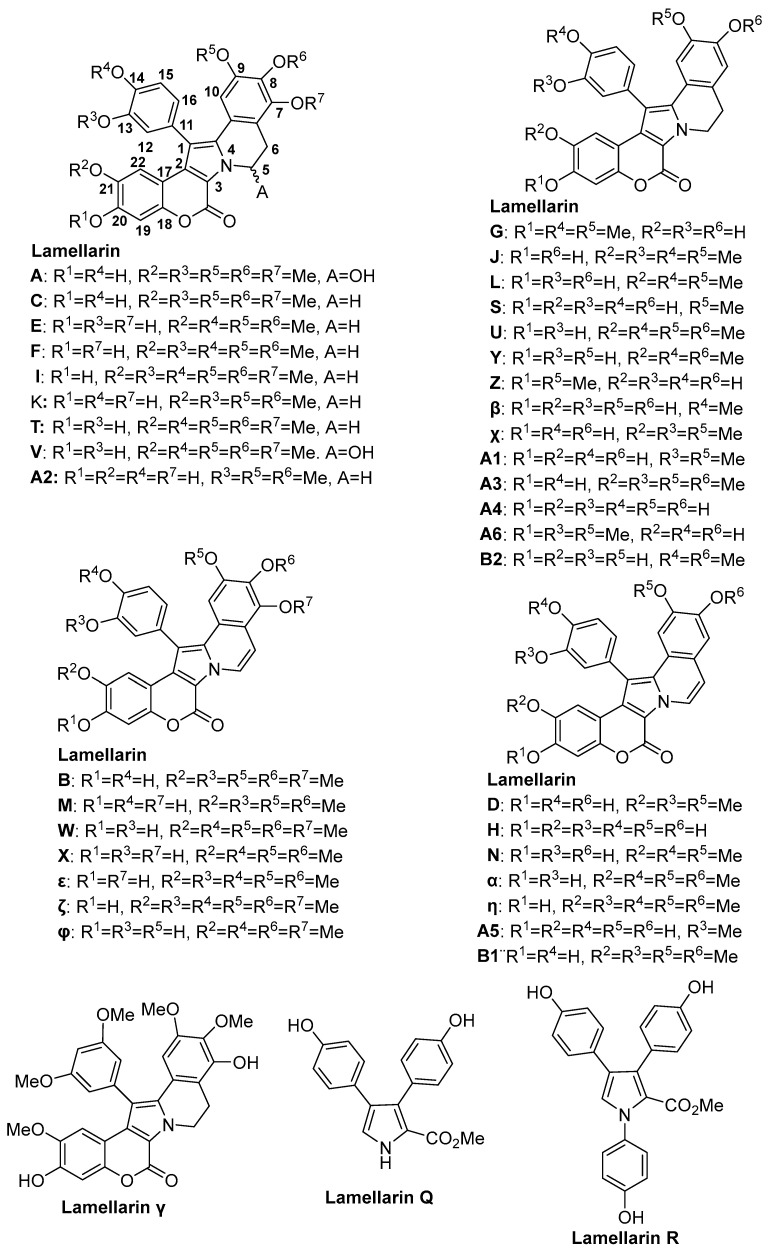
Lamellarins found in nature.

**Figure 2:**
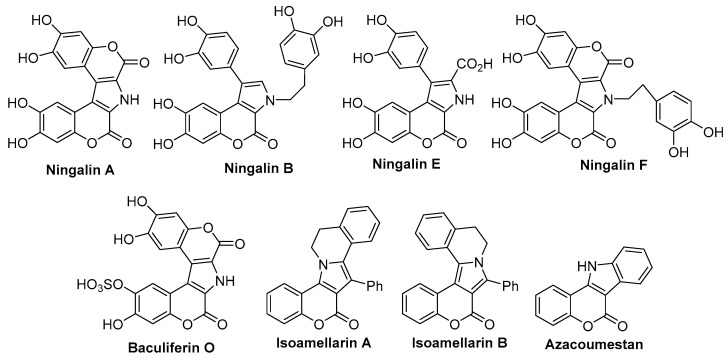
Lamellarin’s related compounds with biological interest.

Coumarin (2*H*-1-benzopyran-2-one) derivatives are widely distributed in nature as secondary metabolites from plants, bacteria, fungi, and marine microorganisms [1,2,3,4,5,6,7,8,9,10,11]. Different derivatives of coumarins, natural or synthetic, possess diverse biological properties [12,13], such as anticancer [14,15], anti-inflammatory [16,17], anticoagulant [18,19], anti-HIV [20,21], antidiabetic [22,23], antibiotic [24,25], antitubercular [26,27], neuroprotective [28,29], etc. Fused coumarins also possess biological activities, and many of them, like furocoumarins [30,31], pyranocoumarins [32], pyridocoumarins [33,34], or pyrrolocoumarins [34,35,36], are isolated from nature. Fused pyrrolocoumarins present interesting biological activities, such as cytotoxic [37,38], antioxidant [39], anti-inflammatory [39,40], multidrug resistance (MDR) reversal [37], and act as benzodiazepine receptor (BZR) ligands [41,42], angiogenesis inhibitors [43], DYRK1A inhibitors [37,44], Wnt signaling inhibitors [45], or fluorescent sensors for live cell imaging [46]. The classes of lamellarins (Figure 1) and their related compounds (Figure 2) are the most important among [3,4]-fused pyrrolocoumarins. Lamellarins have been isolated from diverse marine organisms, such as sponges, ascidians, mollusks, and tunicates, and they are divided into two main groups [47,48,49,50,51]. Type I contains a fused pyrrole ring, while type II has a non-fused pyrrole ring (e.g., lamellarins Q and R). Type I lamellarins involve compounds with the fused piperidine ring being saturated (type Ia) (e.g., lamellarins A, C, G, J, etc.) or with an unsaturated piperidine ring (type Ib) (e.g., lamellarins B, W, D, H, etc.).

More than 70 members of the family of lamellarins have now been isolated. In more detail, lamellarins A–D (Figure 1) were isolated in 1985 from the methanol extracts of the marine prosobranch mollusk, *Lamellaria* sp., by Faukner, Clardy, and coworkers [52]. Lamellarins E–H were identified in 1988 during the chemical investigation of the marine ascidian *Didemnum chartaceum* from the Indian Ocean by Fenical, Clardy et al. [53]. Lamellarins I–M and lamellarin N triacetate were isolated in 1993 by Caroll et al. from the marine ascidian *Didemnum* sp. collected at Southwest Cay, off the North Queensland coast [54]. Lamellarins Q and R were obtained in 1995 by Capon and coworkers from a specimen of *Dendrila Cactos* collected near the coast of New South Wales [55]. In 1996, an Australian tunicate *Didemnum* sp., collected near Duras, New South Wales, yielded the enantiomerically enriched lamellarin S, as reported by Urban and Capon [56]. Lamellarins T–X along with lamellarin Y 20-sulfate were isolated in 1997 by Faukner, Venkateswarlu, and coworkers from an unidentified ascidian (collection # IIC-197) collected in the Arabian Sea [57]. The same group presented the isolation of lamellarin E 20-sulfate and lamellarin α 20-sulfate from the same source in 1999 [58]. In 1999 also, Davis et al. described the isolation of lamellarin Z and lamellarins B 20-sulfate, C 20-sulfate, L 20-sulfate, and G 8-sulfate from the Australian Great Barrier Reef ascidian, *Didemnum* chartaceum [59]. In 2002, lamellarin β was isolated by Ham and Kang from a marine ascidian, *Didemnum* sp., collected in the Indian Ocean [60]. In 2004, Venkateswarlu and coworkers reported the isolation of lamellarins α, γ, and e from the Indian ascidian *Didemnum* obscurum [61]. Lamellarins ζ, η, φ, and χ were also isolated by the same group in 2005 from the Indian tunicate *Didemnum* obscurum [62]. In 2012, lamellarins A1, A2, A3, A4, and A5 were identified by Capon and coworkers during the chemical analysis of *Didemnum* sp. (CMB-01656) collected near Wasp Island, New South Wales, while lamellarin A6 was isolated from another *Didemnum* sp. (CMB-02127) collected from the Northern Rottnest Shelf, Western Australia [63]. In 2019, Bracegirdle et al. presented the isolation of lamellarins D 8-sulfate, E 20-sulfate, K 20-sulfate, A3 20-sulfate, B1 20-sulfate, and B2 20-sulfate from the methanolic extract of Pacific tunicate *Didemnum ternerratum*, collected from the Kingdom of Tonga [64]. 

Ningalins A and B (Figure 2) were isolated, in 1997, by Kang and Fenical from the methanol/chloroform extracts of an ascidian of the genus *Didemnum* collected in Western Australia near Ningaloo Reef [65]. Ningalins E and F were identified in 2012 by Capon and coworkers as components of a *Didemnum* sp. (CMB-02127) collected from the Northern Rottnest Shelf, Western Australia, after its chemical analysis [66]. Baculiferin O was isolated along with other baculiferins, in 2010, by Fan et al. from the Chinese marine sponge *lotrochota baculifera* [67]. The above-mentioned natural products, lamellarins and ningalins, present interesting biological activities, such as cytotoxic, immunomodulatory, anti-HIV-1 virus, antioxidant, antibacterial, and multidrug resistance (MDR) reversal [52,54,57,58,60,61,62,63,64,66,67]. Furthermore, they present promising kinase inhibitory properties [66].

The fused pyrrolo[3,2-*c*]coumarin and pyrrolo[3,4-*c*]coumarin are the core moieties of biologically active isolamellarin A, azacoumestans, and isolamellarin B, respectively (Figure 2) [36,68,69]. In the literature, lamellarins appear in a small number of specific reviews [48,50,51,70,71,72,73,74] or as part of a few reviews [1,36,49,75]. Azacoumestans are also presented in two reviews [36,69]. Herein, we present an overview of the advances demonstrated in the literature on the synthesis and biological evaluation of lamellarins, isolamellarins, and azacoumestans. First, the design and synthesis of those derivatives will be presented, followed by their biological properties.

## 2. Synthetic Strategies for the Preparation of Lamellarins and Related Compounds

The synthetic methods will be presented separately in three parts involving lamellarins, isolamellarins, and azacoumestans, respectively. Similar reaction strategies are used to build pyrrole, isoquinoline, or coumarin rings of those compounds, such as metal-catalyzed cross-coupling, [3 + 2] cycloaddition, substitution, lactonization, cyclocondensation, multicomponent reactions, or *N*-ylide-mediated pyrrole ring formation.

### 2.1. Synthesis of Lamellarins

The synthesis of lamellarins was achieved mainly by pyrrole derivatives or isoquinoline derivatives as starting or intermediate compounds. In a few cases, coumarin derivatives were utilized as starting compounds.

#### 2.1.1. Synthesis Using Pyrrole Derivatives

**Scheme 1:**
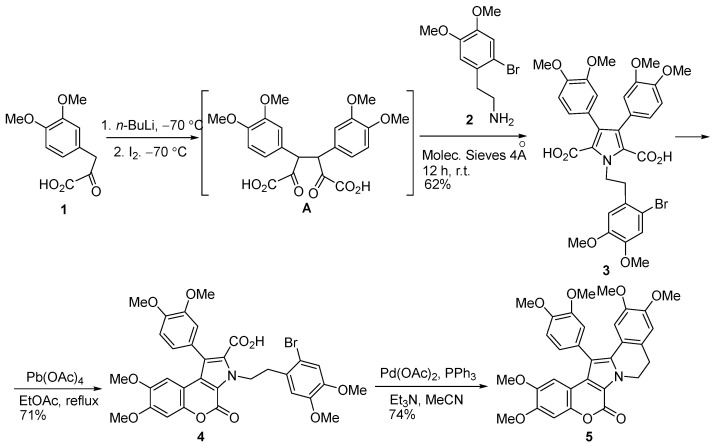
Synthesis of lamellarin G trimethyl ether (**5**).

In 1997, the research group of Prof. Steglich synthesized lamellarin G trimethyl ether (**5**) in 33% total yield in a biomimetic synthesis [76]. Oxidative coupling of two molecules of 3-(3,4-dimethoxyphenyl) pyruvic acid (**1**) furnished intermediate **A**, which in a cyclocondensation reaction with 2-phenylethylamine **2** furnished the pyrrole derivative **3** (Scheme 1). Treatment of **3** with Pb(OAc)_4_ afforded pyrrolo[2,3-*c*]coumarin derivative **4**. Intramolecular Pd-catalyzed Heck coupling reaction of the latter under elimination of CO_2_ resulted in the formation of isoquinoline moiety of lamellarin G trimethyl ether (**5**).

**Scheme 2:**
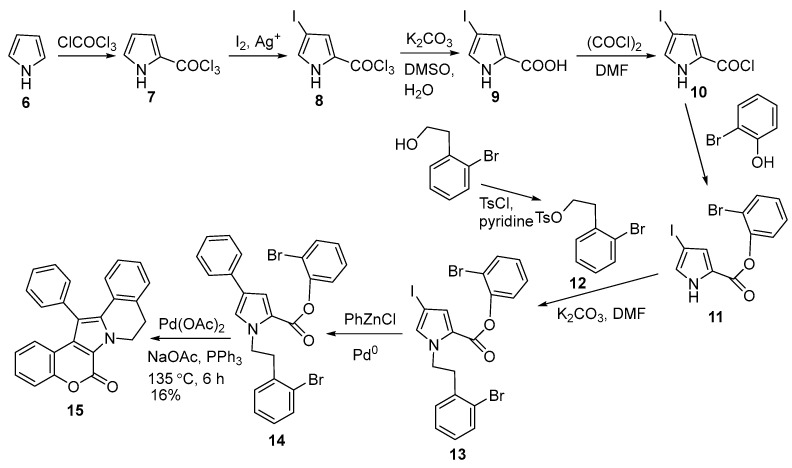
Synthesis of lamellarin framework **15**.

In 1998, Banwell et al. reported the synthesis of lamellarin framework **15** [77]. The synthesis started from the reaction of pyrrole (**6**) with trichloroacetyl chloride to trichloroacetyl derivative **7**, which reacted with iodine in the presence of CF_3_CO_2_Ag to provide 4-iodinated adduct **8** (Scheme 2). Hydrolysis of the latter provided acid **9**. The corresponding chloride **10** by reaction with *o*-bromophenol furnished ester **11**. Alkylation at nitrogen of **11** with tosylate **12** yielded tri-substituted pyrrole **13**. A Negishi cross-coupling of the latter with phenylzinc chloride chemoselectively afforded derivative **14** with 47% overall yield during the above steps. Double Heck cross-coupling reactions of **14** in the presence of Pd(OAc)_2_, PPh_3_, and NaOAc as base at 135 °C resulted in the final product **15** in low yield, 16%.

**Scheme 3:**
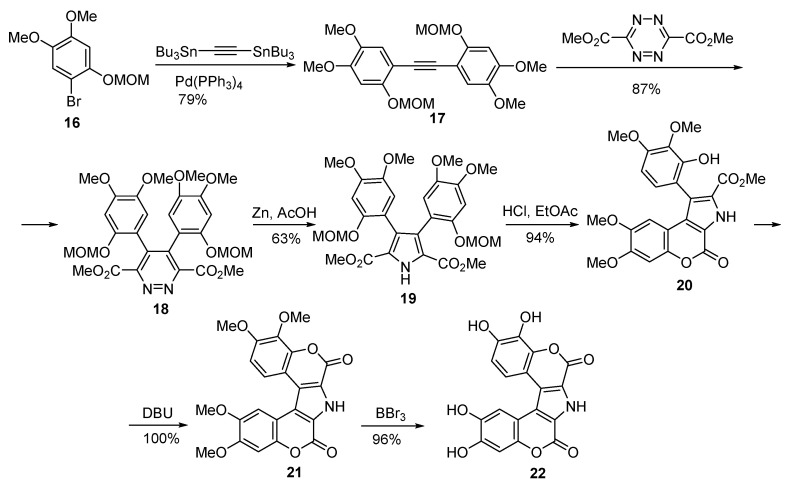
Total synthesis of ningalin A (**22**).

The next year, Boger et al., prepared ningalin A (**22**) using a heterocyclic azadiene Diels–Alder strategy for the construction of pyrrole core [78]. Double Stille cross-coupling of 1-bromo-4,5-dimethoxy-2-(methoxymethoxy)benzene (**16**) with bis-(tributylstannyl)acetylene provided diarylacetylene **17** (Scheme 3). Diels–Alder cycloaddition reaction of **17** with tetrazine derivative afforded diazine adduct **18**. Reductive ring contraction of the latter with zinc furnished pyrrole derivative **19**. Deprotection of MOM ethers with HCl led to monolactone **20**, which with the more forcing conditions of DBU resulted in tetramethyl ningalin A (**21**). Treatment with BBr completed the total synthesis of ningalin A (**22**) in 39% total yield.

In cytotoxicity test against L1210 cytotoxic assay, ningalin A was found to be weakly active.

**Scheme 4:**
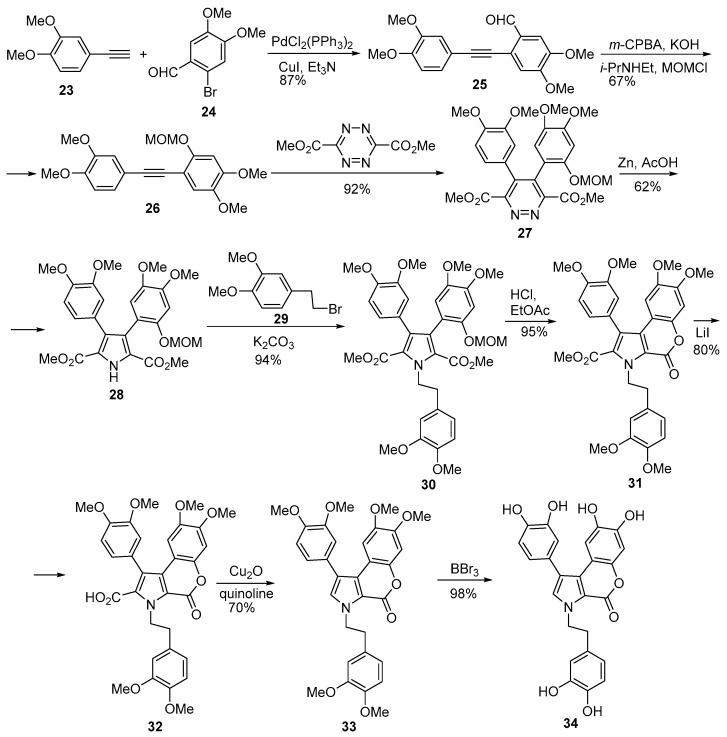
Total synthesis of ningalin B (**34**).

In 2000, Boger et al. again used the heterocyclic azadiene Diels–Alder cycloaddition reaction for the synthesis of ningalin B (**34**) [79]. Sonogashira cross-coupling of acetylene **23** with aldehyde **24** provided alkyne derivative **25**, which by Baeyer–Villinger oxidation and protection of the formed phenol with MOMCl afforded derivative **26** (Scheme 4). Diels–Alder cycloaddition reaction with tetrazine derivative furnished diazine adduct **27**. Reductive ring contraction of the latter with zinc provided pyrrole **28**, which upon *N*-alkylation with aryl ethyl bromide **29** resulted in **30**. Lactonization of **30** led to coumarin derivative **31**. Selective hydrolysis afforded acid **32** and decarboxylation with Cu_2_O in quinoline furnished hexamethyl ningalin B (**33**). Demethylation with BBr_3_ provided the product ningalin B (**34**) in 16% total yield. Compounds **30**, **31**, and **33** reversed the multidrug-resistant (MDR) phenotype, resensitizing a human colon cancer cell line (HCT116/VM46) to vinblastine and doxorubicin.

**Scheme 5:**
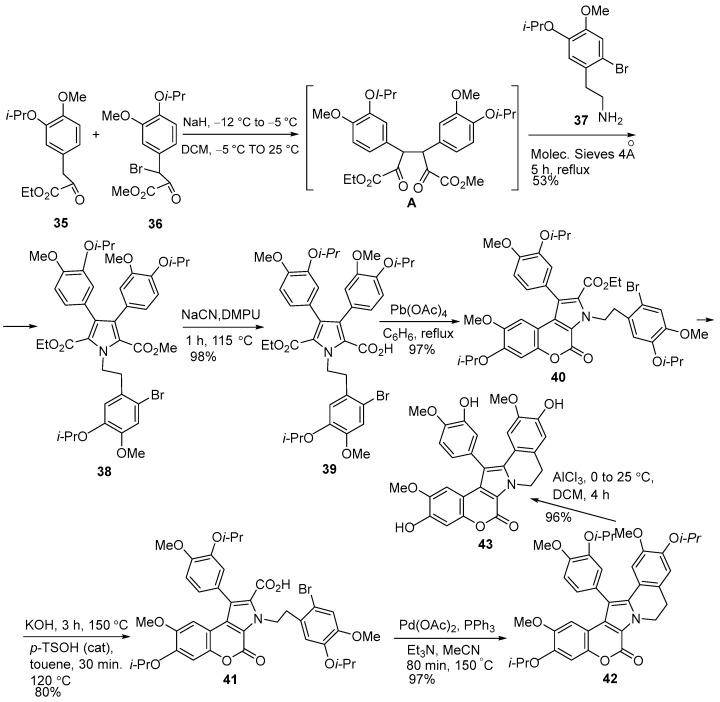
Total synthesis of lamellarin L (**43**).

The same year, Peschko et al. again used aryl puruvic acid derivatives **35**, **36**, and aryl ethyl amine **37** for the construction of pyrrole moiety **38** via cyclocondensation for the synthesis of lamellarin L (**43**) in 38% total yield [80]. Selective hydrolysis of methyl ester of **38** to derivative **39** was achieved by heating in a suspension of NaCN in 1,3-dimethyl-3,4,5,6-tetrahydropyrimidin-2(1*H*)-one (DMPU) (Scheme 5). Oxidation of pyrrole **39** with Pb(OAc)_4_ afforded pyrrolo[2,3-*c*]coumarin derivative **40**. Hydrolysis of the latter, to give adduct **41**, followed by intramolecular Pd-catalyzed Heck cross-coupling reaction, and elimination of CO_2_ furnished the isoquinoline moiety **42**. Finally, treatment of **42** with AlCl_3_ removed the isopropyl groups producing lamellarin L (**43**).

**Scheme 6:**
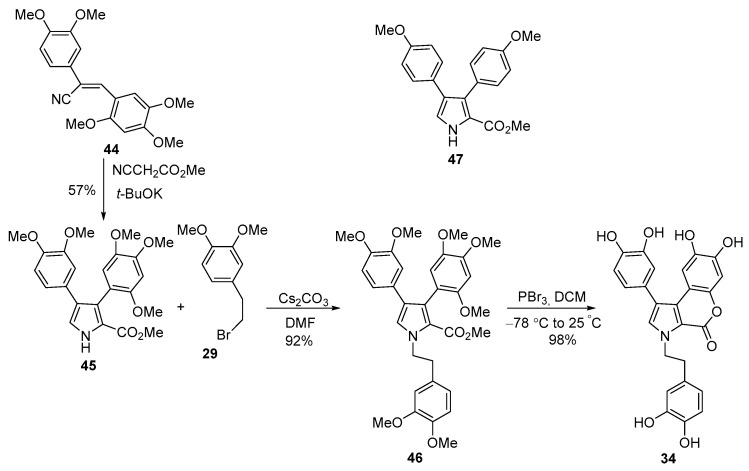
Total synthesis of ningalin B (**34**).

Bullington et al. prepared ningalin B (**34**) in excellent yield, 90%, starting from pyrrole derivative **45** [81]. Pyrrole **45** is analogous to Furstner intermediate **47** [82], prepared by a [3 + 2] cycloaddition reaction of methyl isocyanoacetate with α,β-unsaturated nitrile **44** (Scheme 6).

**Scheme 7:**
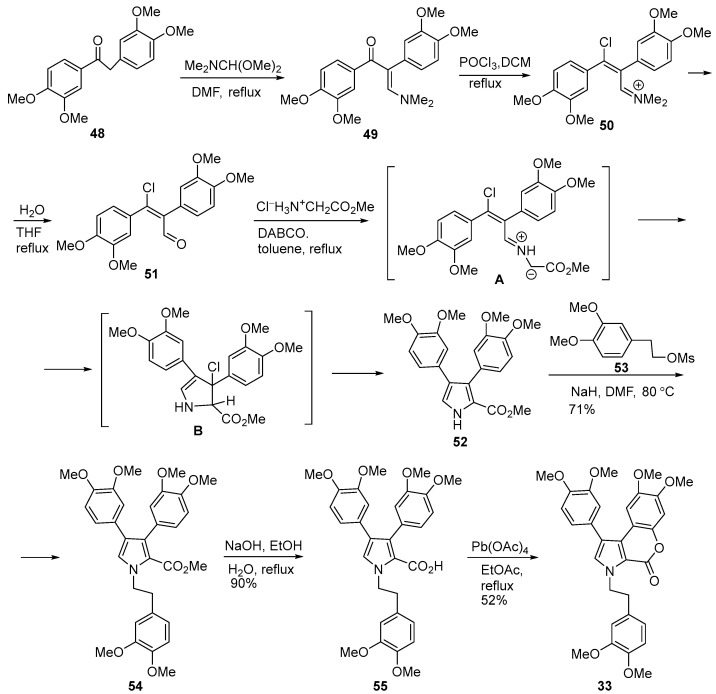
Synthesis of ningalin B hexamethyl ether (**33**).

In 2003, Gupton et al. provided an alternative pathway for the synthesis of ningalin B hexamethyl ether (**33**) in 18% overall yield, acting as multidrug reversal (MDR) agent [83]. The trisubstituted pyrrole derivative **52**, analogous to Furstner intermediate **47**, was prepared regioselectively starting from desoxyveratroin (**48**) (Scheme 7). The latter after reaction with *N*,*N*-dimethyl formamide dimethyl acetal furnished enamine **49**, which upon chlorination with POCl_3_ to **50** and hydrolysis afforded β-chloroenal **51**. Reaction of **51** with glycine methyl ester hydrochloride resulted in pyrrole **52** via the vinylogous iminium salt intermediate **A** and internal 1,3-dipolar cycloaddition reaction to dihydro pyrrole **B**. Alkylation of pyrrole **52** with mesylate ester **53** provided *N*-aryl ethyl pyrrole **54**, which upon basic hydrolysis led to acid **55**. Oxidation of the latter with lead tetraacetate, as in Scheme 1, resulted in ningalin B hexamethyl ether (**33**).

**Scheme 8:**
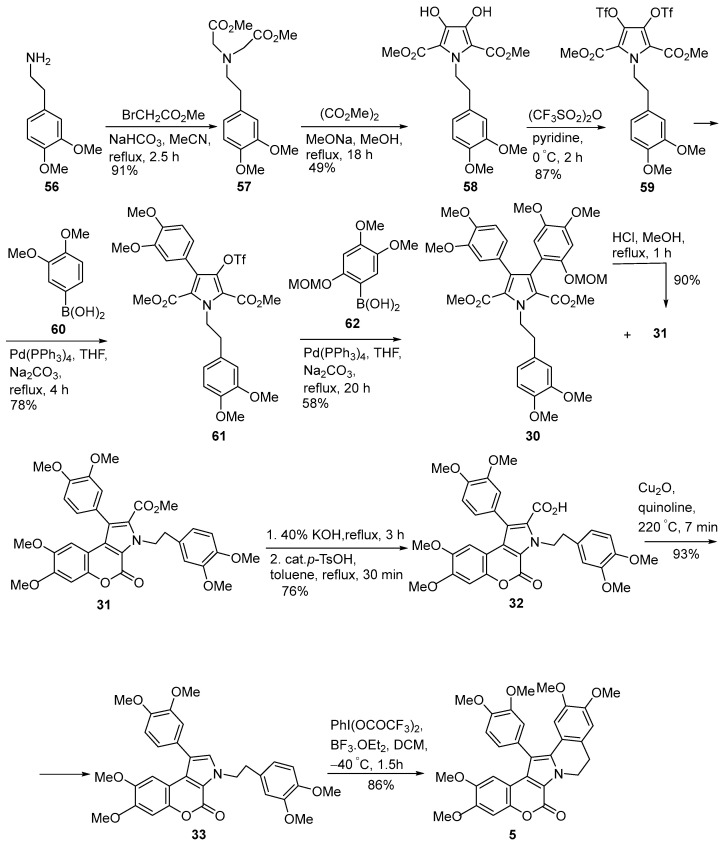
Synthesis of ningalin B hexamethyl ether (**33**) and lamellarin G trimethyl ether (**5**).

The same year, Iwao et al. achieved the synthesis of ningalin B hexamethyl ether (**33**) in 13.1% overall yield and lamellarin G trimethyl ether (**5**) in 11.3% overall yield using as key reactions the Hinsberg-type pyrrole **58** synthesis and the palladium-catalyzed Suzuki cross-coupling of 3,4-dihydroxypyrrole bis-triflate derivative **59** [84]. The Hinsberg-type cyclocondensation of aminodiacetate **57** with methyl oxalate provided the 3,4-dihydroxypyrrole derivative **58**, which afforded 3,4-bis-triflate adduct **59** (Scheme 8). Pd-catalyzed Suzuki cross-coupling of the latter with arylboronic acid **60** furnished 4-arylpyrrole derivative **61**, which with a second Suzuki coupling with arylboronic acid **62** led to 3,4-diarylpyrrole compound **30** and coumarin derivative **31**. Derivative **30** by treatment with hydrochloric acid resulted also in coumarin compound **31**. Hydrolysis of **31** to **32** followed by decarboxylation led to ningalin B hexamethyl ether (**33**). Treatment of the latter with phenyliodine bis(trifluoroacetate) (PIFA) provided lamellarin G trimethyl ether (**5**).

**Scheme 9:**
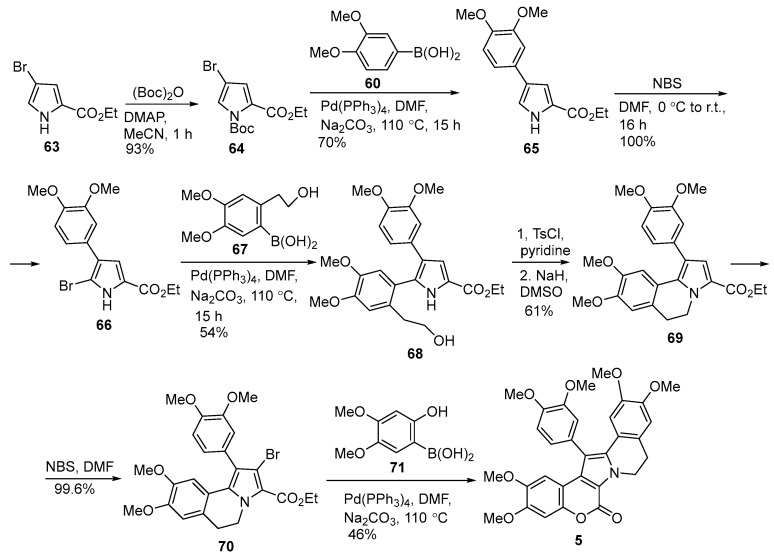
Synthesis of lamellarin G trimethyl ether (**5**).

Handy et al. reported the synthesis of lamellarin G trimethyl ether (**5**) in 11 steps and 9% total yield using three sequential halogenation/Suzuki cross-coupling reactions [85]. Protection of bromopyrrole derivative **63** with (Boc)_2_O and cross-coupling of the adduct **64** with excess of boronic acid **60** furnished arylpyrrole compound **65** (Scheme 9). Bromination with NBS regioselectively to bromopyrrole **66** followed by Suzuki coupling with boronic acid **67**, without the protection of hydroxy group, afforded 4,5-diarylpyrrole derivative **68**. Intramolecular alkylatioin through the corresponding tosylate led to isoquinoline derivative **69**. Bromination of the latter to **70** and Suzuki coupling with excess of boronic acid **71**, added in two portions, resulted in lamellarin G trimethyl ether (**5**).

**Scheme 10:**
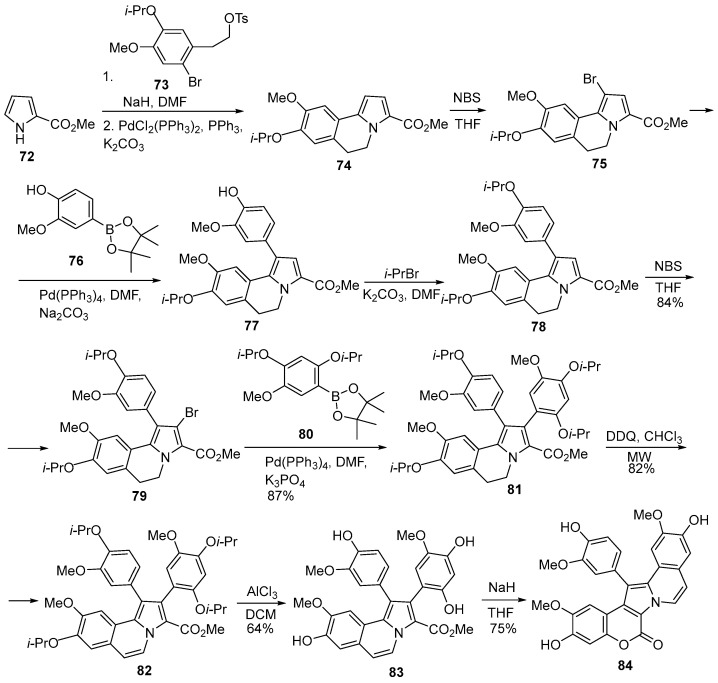
Synthesis of lamellarin D (**84**).

Pla et al. [86] synthesized the cytotoxic lamellarin D (**84**) in 8 steps and 18% overall yield from methyl pyrrole-2-carboxylate (**72**) using sequential bromination/Suzuki cross-coupling reactions like the above-mentioned procedure by Handy [85]. *N*-alkylation of **72** with tosylate **73** followed by Heck cross-coupling and cyclization furnished dihydroisoquinoline framework **74** (Scheme 10). Regioselective bromination with NBS provided bromopyrrole **75**, which under Suzuki cross-coupling with boronic ester **76** afforded 4-arylpyrrole adduct **77**. Protection of phenol **77** to provide **78** and bromination to **79** followed by Suzuki coupling with boronate **80** led to 3,4-diarylpyrrole derivative **81**. Oxidation with DDQ produced isoquinoline derivative **82**, which by deprotection to **83** and cyclization resulted in the formation of coumarin moiety of lamellarin D (**84**).

Lamellarin D (**84**) was earlier reported to have good cytotoxic activity against different human tumor cell lines and to be a lead candidate for the development of topoisomerase I-targeted antitumor agents [87].

**Scheme 11:**
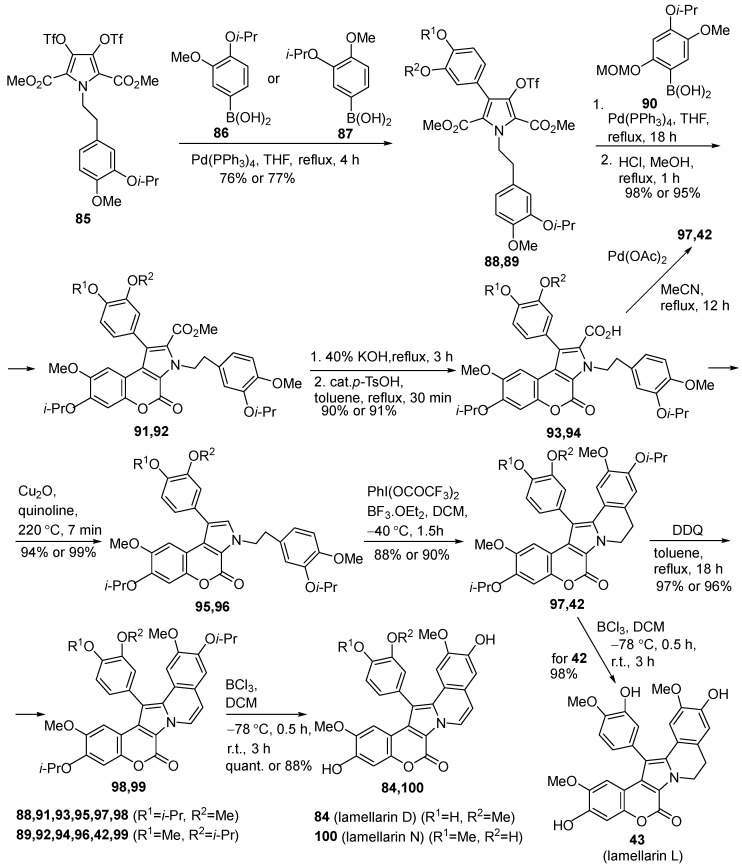
Synthesis of lamellarins D (**84**), L (**43**), and N (**100**).

Fujikawa et al. presented [88] the synthesis of lamellarins D (**84**), L (**43**), and N (**100**) in 54, 58, and 50% yields, respectively, from their common precursor **85** using the procedure utilized earlier (Scheme 8) from their group [84]. Pyrrole derivative **85** was prepared by Hinsberg-type pyrrole synthesis [88], and Suzuki cross-coupling with boronic acids **86** or **87** provided monoaryl pyrrole derivatives **88** or **89**, respectively (Scheme 11). A second Suzuki coupling with boronic acid **90** followed by treatment with HCl afforded coumarin adducts **91** or **92**, which by hydrolysis furnished acids **93** or **94**. Decarboxylation of **95** or **96** followed by oxidative cyclization by PIFA led to **97** or **42**. Oxidation by DDQ provided isoquinoline products **98** or **99** and by deprotection of isopropoxy group by BCl_3_ resulted in lamellarin D (**84**) or lamellarin N (**100**). The corresponding deprotection of **42** produced lamellarin L (**43**). Treatment of **93** or **94** with palladium acetate also provided dihydroisoquinoline derivatives **97** or **42**.

**Scheme 12:**
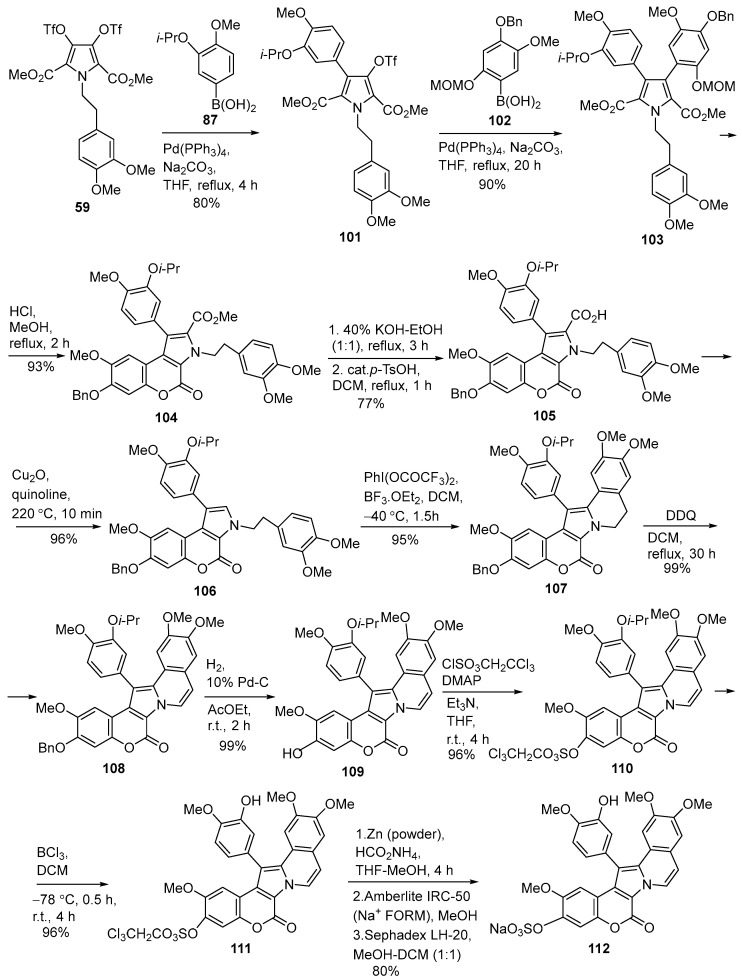
Synthesis of lamellarin α 20-sulfate (**112**).

The same group also reported the synthesis of lamellarin α 20-sulfate (**112**), selective inhibitor of HIV-1 integrase, in 24% total yield in a process similar to that mentioned above using as starting compound pyrrole derivative **59** [89]. Suzuki cross-coupling with boronic acid **87** provided 4-arylpyrrole adduct **101** and a second cross-coupling with boronic acid **102** led to 3,4-diarylpyrrole derivative **103** (Scheme 12). Treatment of the latter with concentrated HCl afforded coumarin derivative **104**, which upon hydrolysis to **105** followed by decarboxylation to **106** and oxidative cyclization furnished dihydroisoquinoline derivative **107**. Oxidation by DDQ produced isoquinoline adduct **108**. Sequential deprotection of benzyl group upon hydrogenolysis under Pd-C to **109**, reaction with trichloroethyl chlorosulfate to **110**, selective deprotection of isopropyl group with BCl_3_ to **111**, and finally reductive deprotection of trichloroethyl ester with Zn/HCO_2_NH_4_ followed by ion exchange under Amberlite resulted in lamellarin α 20-sulfate (**112**).

**Scheme 13:**
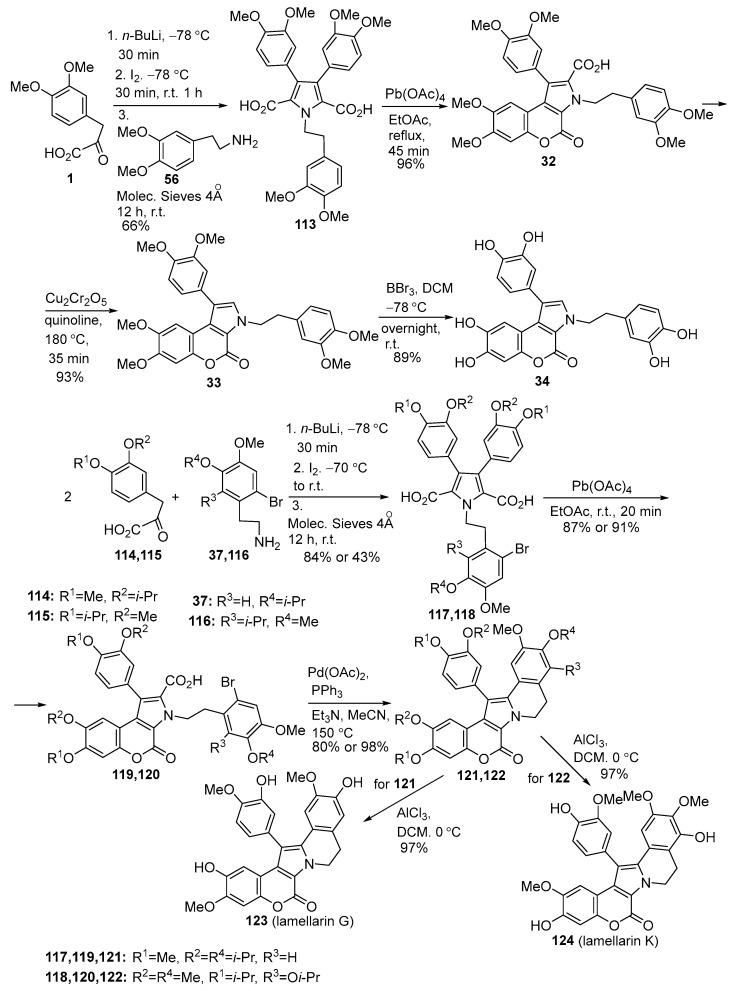
Synthesis of ningalin B (**34**), lamellarin G (**123**), and lamellarin K (**124**).

Peschko et al. developed the synthesis of marine alkaloids ningalin B (**34**), lamellarin G (**123**), and lamellarin K (**124**) in 52%, 56%, and 37% overall yields, respectively, utilizing as key step their earlier-applied method for the formation of 3,4-diarylpyrrole-2,5-dicarboxylic acids **113**, **117**, and **118** by cyclocondensation from arylpuruvic acids **1**, **114**, and **115** and 2-arylethylamines **56**, **37**, and **116**, respectively [90]. Oxidation of acids **113**, **117**, and **118** by lead tetraacetate led to coumarin derivatives **32**, **119**, and **120** (Scheme 13). Decarboxylation of **32** with copper chromite to **33** and deprotection with BBr_3_ resulted in ningalin B (**34**). Palladium-catalyzed Heck reaction and cyclization of **119** and **120** afforded the pentacyclic lamellarin derivatives **121** and **122**, respectively. Selective deprotection of isopropyl group of compounds **121** and **122** under treatment with aluminum trichloride resulted in lamellarin G (**123**) and lamellarin K (**124**), respectively.

**Scheme 14:**
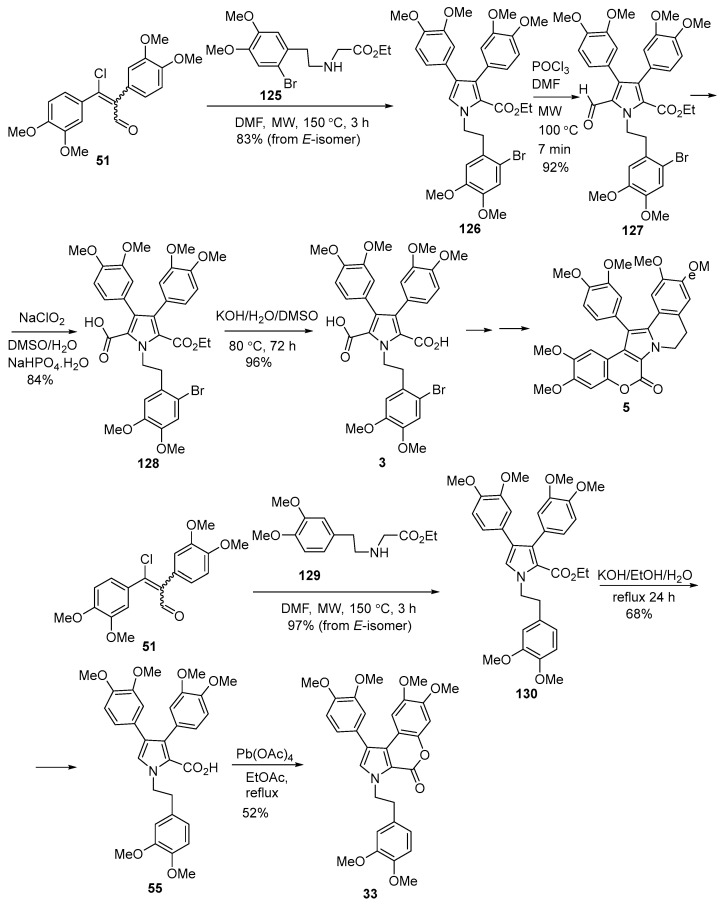
Synthesis of lamellarin G trimethyl ether (**5**) and ningalin B hexamethyl ether (**33**).

In 2009, Gupton et al. presented an alternative procedure for the synthesis of Steglich synthon **3** (Scheme 1), intermediate for the formation of lamellarin G trimethyl ether (**5**) [91]. The reaction of β-chloroenal **51** with alkyl glycine ester **125** to tetra-substituted pyrrole **126** was the key step for the construction of Steglish synthon **3** (Scheme 14). Vilsmeyer–Haack–Arnold formylation under microwave irradiation provided pyrrole derivative **127**, which by oxidation with sodium chlorite to **128** and hydrolysis afforded **3**. Ningalin B hexamethyl ether (**33**) was also synthesized by the reaction of aldehyde **51** with glycine ester **129** to pyrrole derivative **130**, followed by hydrolysis to pyrrole-2-carbocylic acid **55**, which by oxidation with lead tetraacetate resulted in product **33**.

**Scheme 15:**
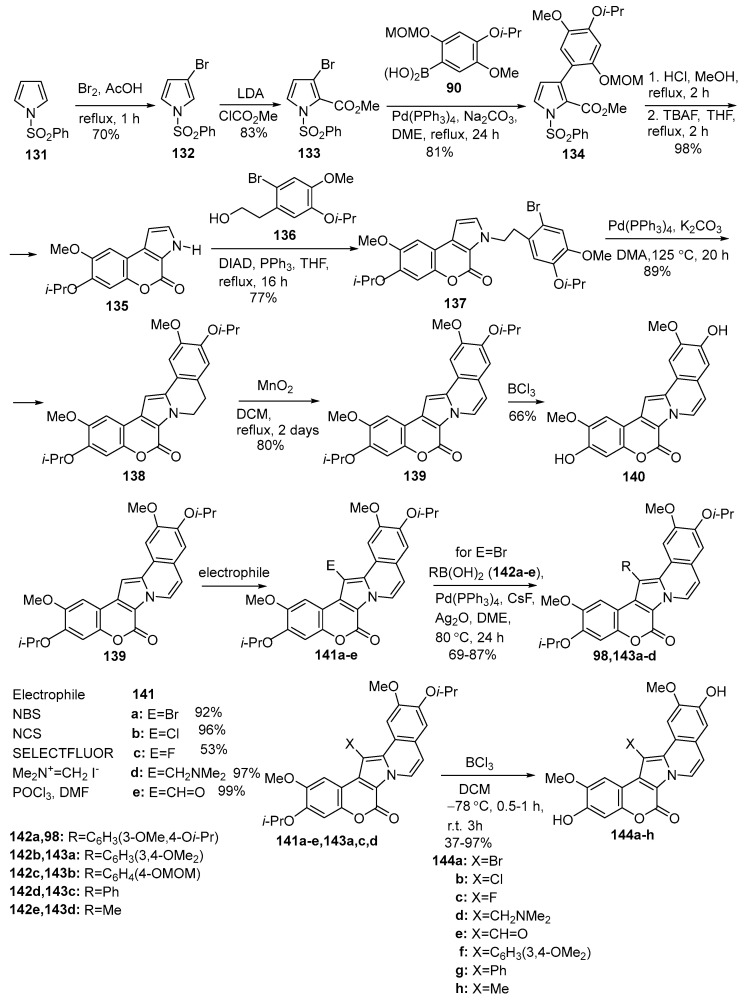
Synthesis of **140** and **144a**–**h**, analogues of lamellarin D.

The same year, Ohta et al. designed and synthesized the analogues of lamellarin D **140** and **144a**–**h** as inhibitors of topoisomerase I [92]. The key pentacycle intermediate **138** was obtained by intramolecular Heck reaction of pyrrolo[2,3-*c*]coumarin derivative **137** (Scheme 15). The synthesis started from bromination of pyrrole **131** to **132** followed by selective lithiation and reaction with methyl chloroformate to provide pyrrole derivative **133**. Palladium-catalyzed Suzuki cross-coupling reaction with **90** afforded **134**, which by hydrolysis and cyclization provided the coumarin derivative **135**. *N*-alkylation led to the intermediate compound **137**. Oxidation of **138** to isoquinoline derivative **139** and selective deprotection of isopropoxy group with BCl_3_ resulted in the non-substituted lamellarin D analogue **140**. Reaction with electrophiles of **139** provided derivatives **141a**–**e** in 53–99% yields. Suzuki cross-coupling of bromo derivative **141a** with boronic acids **142a**–**e** furnished derivatives **98** and **143a**–**d** in 69–82% yields. Selective deprotection of intermediates **141a**–**e** and **143a**,**c**,**d** resulted in the lamellarin D analogues **144a**–**h**.

Derivatives **140** and **144a**–**e**,**h** were tested for their antiproliferative activity and found to be as potent as parent compound lamellarin D (**84**) against 39 different human cancer cell lines [93].

**Scheme 16:**
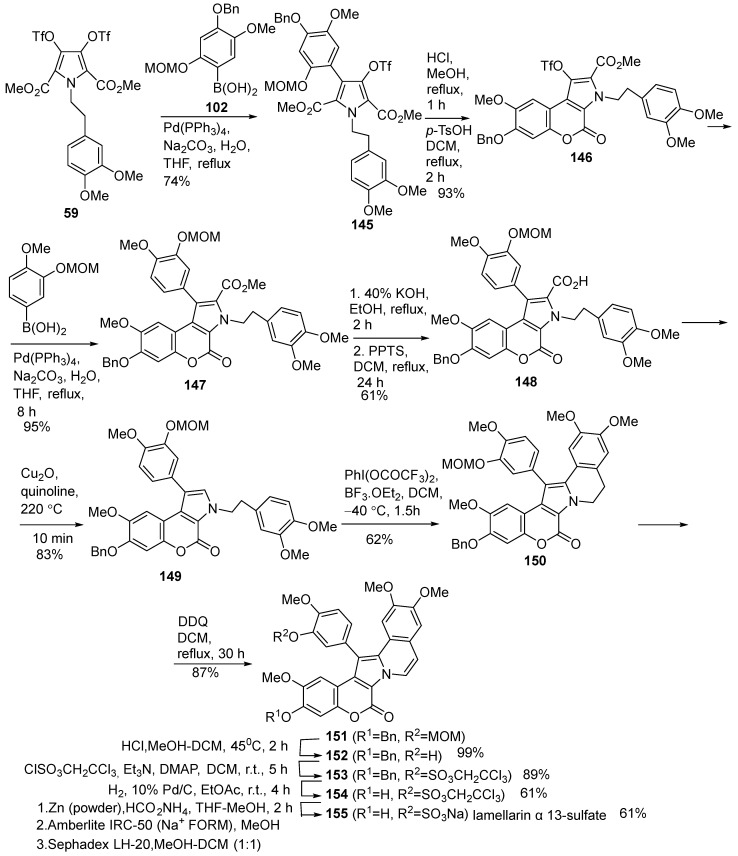

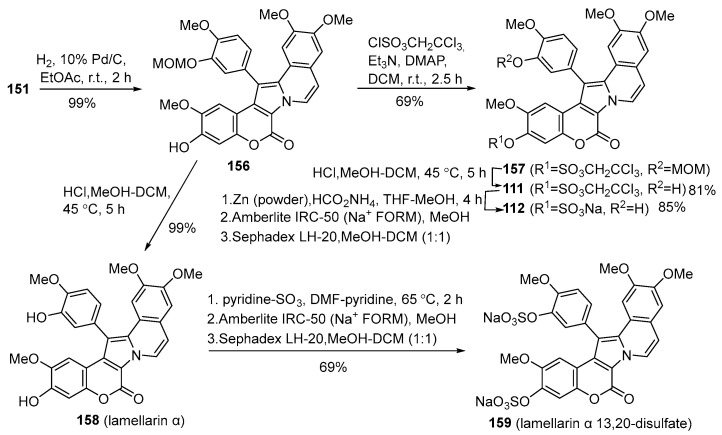
Synthesis of lamellarin α (**158**) and lamellarin α 13-sulfate (**155**), 20-sulfate (**112**), and 13,20-disulfate (**159**).

Fukuda et al. presented the synthesis of lamellarin α (**158**) and lamellarin α 13-sulfate (**155**), 20-sulfate (**112**), and 13,20-disulfate (**159**) using as common intermediate compound **151** with two different protecting groups, benzyl for 7-hydroxy coumarin moiety and methoxymethyl for 3-hydroxyphenyl moiety [94]. The reaction sequence started from penta-substituted pyrrole **59**, which by Suzuki cross-coupling with boronic acid **102** provided arylpyrrole derivative **145**. Hydrolysis and cyclization of **145** led to pyrrolo[2,3-*c*]coumarin **146**. A new Suzuki reaction with MOM-protected boronic acid afforded derivative **147**. Hydrolysis of the latter followed by decarboxylation, oxidation–cyclization with PIFA, and aromatization by DDQ resulted in the common intermediate **151** (Scheme 16).

Deprotection of MOM group of **151** with hydrochloric acid to 3-hydroxyaryl derivative **152** and reaction with 2,2,2-trichloroethyl chlorosulfate furnished sulfate **153**, which by hydrogenolysis and reductive deprotection of 2,2,2-trichloroethyl ester **154** with Zn-HCO_2_NH_4_ followed by ion exchange with Amberlite and Sephadex purification resulted in lamellarin α 13-sulfate (**155**) in 6% overall yield from **59**. Intermediate **151** under hydrogenolysis to hydroxycoumarin derivative **156** and reaction with 2,2,2-trichloroethyl chlorosulfate provided ester **157**, which by MOM deprotection led to 3-hydroxyaryl compound **111** (Scheme 16). Treatment, in analogy to the above-mentioned transformation of **154** to **155**, afforded lamellarin α 20-sulfate (**112**) in 8% total yield from **59**. Deprotection of MOM group of **156** with hydrochloric acid resulted in lamellarin α (**158**) in 12% overall yield from **59**, which by the reaction with pyridine-SO_3_ complex and ion exchange with Amberlite and purification by Sephadex led to lamellarin α 13,20-disulfate (**159**) in 8% total yield from **59**.

**Scheme 17:**
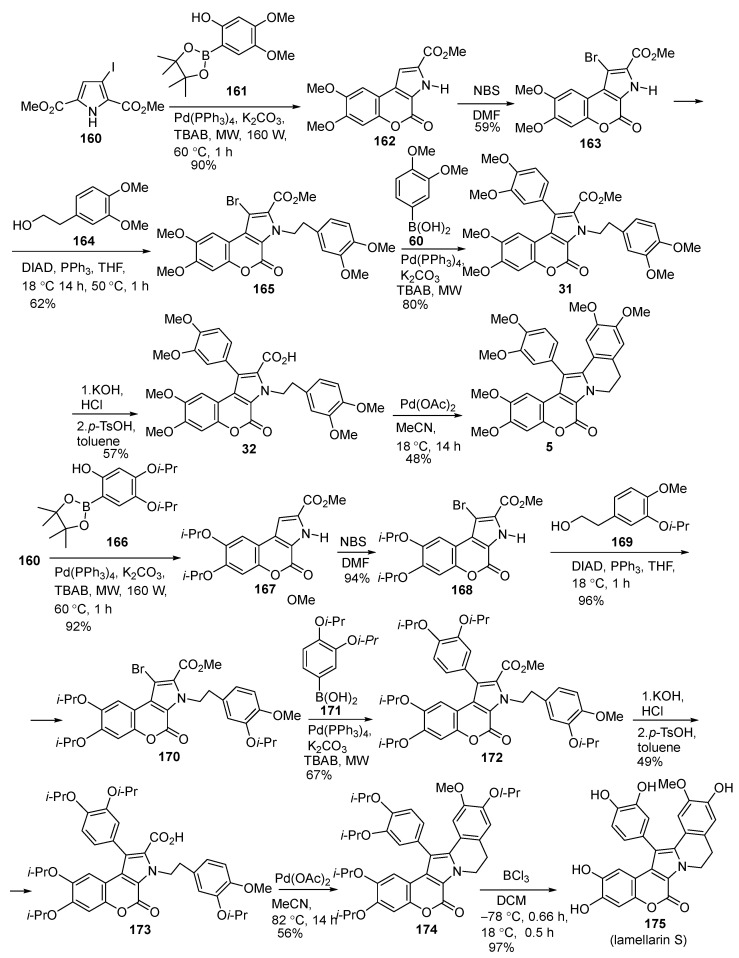
Synthesis of lamellarin G trimethyl ether (**5**) and lamellarin S (**175**).

Hasse et al. synthesized lamellarin G trimethyl ether (**5**) and lamellarin S (**175**) in 7% and 15% overall yields, respectively, starting from the iodopyrrole derivative **160** [95]. Suzuki cross-coupling reaction of **160** with boronic acid **161** followed by spontaneous lactonization afforded pyrrolo[2,3-*c*]coumarin derivative **162**, which by selective bromination with NBS provided bromide **163** (Scheme 17). *N*-alkylation of the latter under Mitsunobu conditions furnished derivative **165**, which by a new Suzuki coupling with boronic acid **60** generated compound **31**. Compound **31**, following the former method by Iwao [84], led to acid **32**, which by decarboxylative Heck cross-coupling reaction resulted in lamellarin G trimethyl ether (**5**). Lamellarin S pentamethyl ether (**174**) was formed under a similar procedure starting from iodopyrrole derivative **160** and using boronic acids **166** and **171** and 2-arylethanol **169**. Selective deprotection of isopropoxy group of **174** with BCl_3_ produced lamellarin S (**175**).

**Scheme 18:**
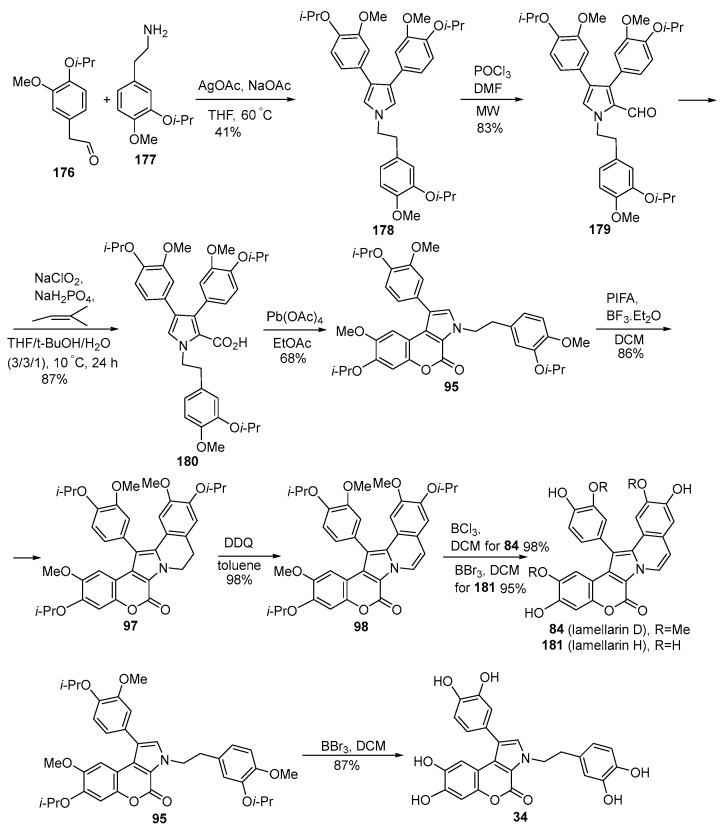
Synthesis of lamellarins D (**84**) and H (**181**) and ningalin B (**34**).

Li et al. reported the total synthesis of lamellarins D (**84**) in 16.6% and H (**181**) in 16.1% and ningalin B (**34**) in 15% overall yields from 2-(4-isopropoxy-3-methoxyphenyl) acetaldehyde (**176**) and 2-(3-isopropoxy-4-methoxyphenyl) ethylamine (**177**) [96]. The key step for this procedure is at first AgOAc-mediative oxidative coupling of **176** and cyclocondensation with **177** to form pyrrole derivative **178** followed by a microwave-accelerated Vilsmaier–Haack reaction to aldehyde **179**, which by Lindgren oxidation at 10 °C led to acid **180** (Scheme 18). A second oxidative cyclization of the latter with Pb(OAc)_4_ provided pyrrolo[2,3-*c*]coumarin compound **95**. Deprotection of **95** afforded ningalin B (**34**). The third oxidative cyclization of **95** upon treatment with PIFA afforded dihydro isoquinoline adduct **97**, which by oxidation with DDQ furnished isoquinoline product **98**. Selective deprotection led to lamellarin D (**84**), while deprotection with BBr_3_ resulted in lamellarin H (**181**).

**Scheme 19:**
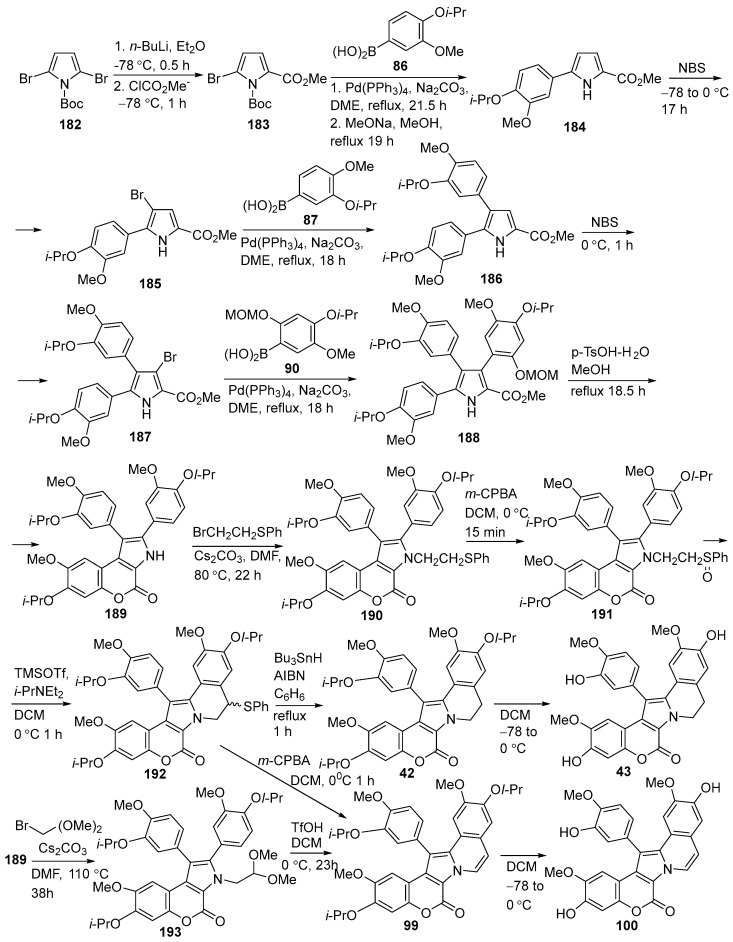
Synthesis of lamellarins L (**43**) and N (**100**).

In 2014, Komatsubara et al., presented the synthesis of lamellarins L (**43**) and N (**100**) using as starting compound 3,4,5-differentially triarylated pyrrole-2-carboxylate as the **188** [97]. The 5-bromopyrrole-2-carboxylate **183** was synthesized from 2,5-dibromo-*N*-Bocpyrrole (**182**) through Br-Li exchange and methoxycarbonylation. This compound then underwent Suzuki cross-coupling reaction with boronic acid **86** to yield the 5-arylpyrrole-2-carboxylate derivative **184** (Scheme 19). Bromination with NBS to **185** and a second cross-coupling reaction with boronic acid **87** provided 4,5-diaryl pyrrole adduct **186**. Bromination of the latter with NBS furnished 3-pyrrole derivative **187**, which was followed by a new Suzuki coupling with **90** to 3,4,5-triaryl pyrrole compound **188**. Treatment with *p*-TsOH led to pyrrolo[2,3-*c*]coumarin compound **189**. *N*-thioalkylation with bromoethyl phenyl sulfide provided thioether **190**. Oxidation of the latter to **191** and Pummerer cyclization led to dihydroisoquinoline derivative **192**. Radical desulfurization of **192** with Bu_3_SnH/AIBN provided lamellarin L triisopropyl ether (**42**), which by treatment with BCl_3_ resulted in lamellarin L (**43**) in 29% total yield. Treatment of **192** with m-CPBA furnished the lamellarin N triisopropyl ether (**99**) and by selective hydrolysis with BCl_3_ generated lamellarin N (**100**) in 34% total yield. In an alternative procedure for the synthesis of lamellarin N (**100**) in 42% overall yield, coumarin intermediate **189** was treated with bromoacetaldehyde dimethyl acetal to provide derivative **193**, which was cyclized in the presence of TfOH to provide triisopropyl ether of lamellarin N **99**.

**Scheme 20:**
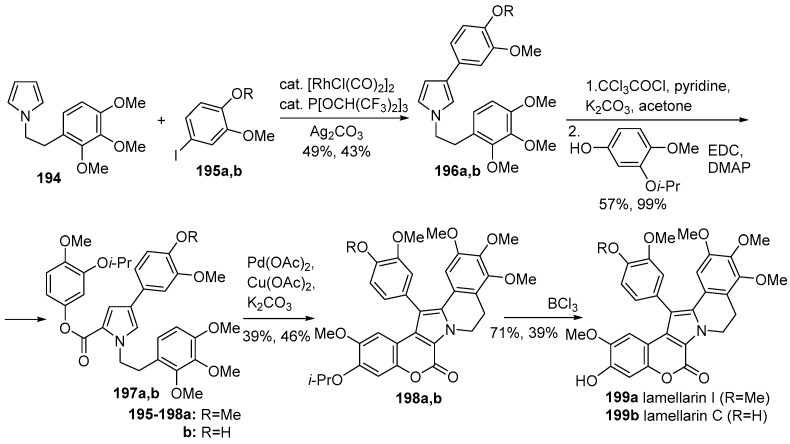
Synthesis of lamellarins I (**199a**) and C (**199b**).

Ueda et al. completed the synthesis of lamellarins I (**199a**) and C (**199b**) using the Rh-catalyzed β-selective cross-coupling arylation of pyrroles [98]. Pyrrole derivative **194** reacted with aryl iodides **195a**,**b** and selectively provided 3-arylpyrrole adducts **196a**,**b** under Rh-catalysis (Scheme 20). Reactions of the latter with trichloroacetyl chloride followed by hydrolysis and esterification with 3-isopropoxy-4-methoxyphenol afforded pyrrole-2-carboxylates **197a**,**b**, which by oxidative coupling and double C-C bond formation in the presence of stoichiometric Pd(OAc)_2_, Cu(OAc)_2_, and K_2_CO_3_ furnished the isopropyl ethers of lamellarins I and C **198a**,**b**. Finally, deprotection of isopropoxy group resulted in lamellarins I (**199a**) and C (**199b**).

**Scheme 21:**
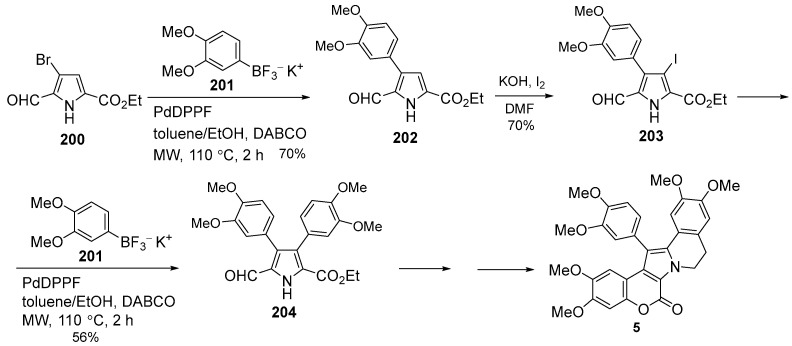
Synthesis of lamellarin G trimethyl ether (**5**).

Gupton et al. used the formyl group activation strategy of 5-formylpyrrole-2-carboxylates for the regioselective introduction of different building blocks through Suzuki cross-coupling reactions. This method was used to synthesize lamellarin G trimethyl ether (**5**) and other natural products [99]. Suzuki coupling of ethyl 4-bromo-5-formylpyrrole-2-carboxylate (**200**) with boron compound **201** provided 4-aryl pyrrole derivative **202**, which by iodination afforded 3-iodopyrrole compound **203** (Scheme 21). New Suzuki reaction with **201** generated 3,4-diarylpyrrole adduct **204**, which was the intermediate for the synthesis of derivatives **127**, **128**, and **3** (Scheme 14) and finally the formation of lamellarin G trimethyl ether (**5**).

**Scheme 22:**
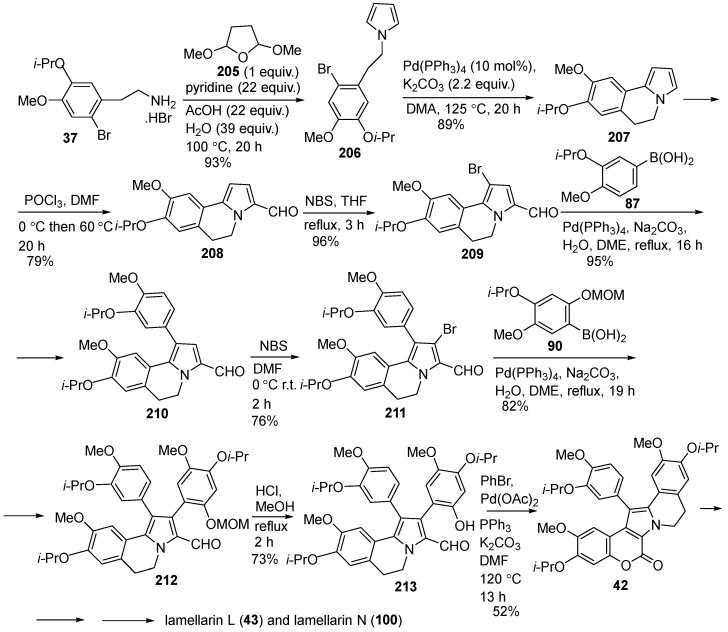
Synthesis of lamellarin L (**43**) and lamellarin N (**100**).

Fukuda et al. synthesized lamellarin L (**43**) and lamellarin N (**100**) through the Paal–Knorr synthesis of pyrrole derivative **206** by the reaction of aryl ethyl amine hydrobromide **37** and one equivalent of 2,5-dimethoxyfuran (**205**) (Scheme 22). Pd-catalyzed direct intramolecular arylation of the latter provided 5,6-dihydropyrrolo[2,1-α]isoquinoline scaffold **207** [100]. Vilsmeier–Haack formylation to **208** followed by bromination with NBS afforded aldehyde **209**, which by Suzuki cross-coupling with boronic acid **87** furnished arylpyrrole compound **210**. A new bromination to **211** and a next cross-coupling with boronic acid **90** led to diarylpyrrole derivative **212**. Hydrolysis with HCl followed by cyclization of the resulted phenolic aldehyde **213** upon treatment with Pd(OAc)_2_, bromobenzene, PPh_3_, and K_2_CO_3_ generated lamellarin L triisopropyl ether (**42**) in 14% overall yield. This was the intermediate (Scheme 11) for the former synthesis of lamellarin L (**43**) and lamellarin N (**100**) [87].

**Scheme 23:**
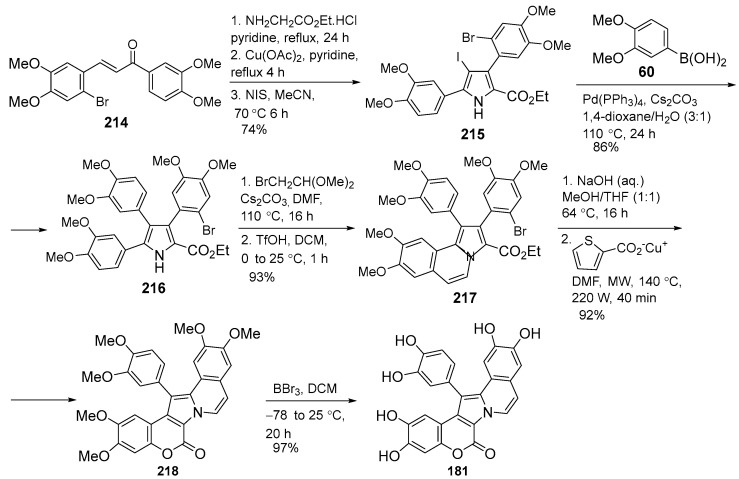
Synthesis of lamellarin D trimethyl ether (**218**) and lamellarin H (**181**).

Pyrrole core **215** was the key step for the synthesis of lamellarin D trimethyl ether (**218**) and lamellarin H (**181**) presented by Dialer et al. [101]. The electrocyclic ring closure of the adduct generated in situ from the chalcone **214** and glycine ethyl ester hydrochloride in the presence of Cu(OAc)_2_ and NIS afforded pyrrole **215**. Suzuki cross-coupling reaction of **215** with boronic acid **60** afforded 3,4,5-triaryl substituted pyrrole-2-carboxylate **216** (Scheme 23). *N*-alkylation of the latter with bromoacetaldehyde dimethyl acetal followed by intramolecular cyclization in the presence of triflic acid in a Pomeranz–Fritsch one-pot reaction led to isoquinoline product **217**. After saponification and cyclization of sodium salt with copper (I) thiophene-2-carboxylate under microwave irradiation in an Ullmann-type reaction, the lamellarin D trimethyl ether (**218**) was obtained in 54% yield from chalcone. Deprotection with BBr_3_ resulted in lamellarin H (**181**) in 53% yield from chalcone.

**Scheme 24:**
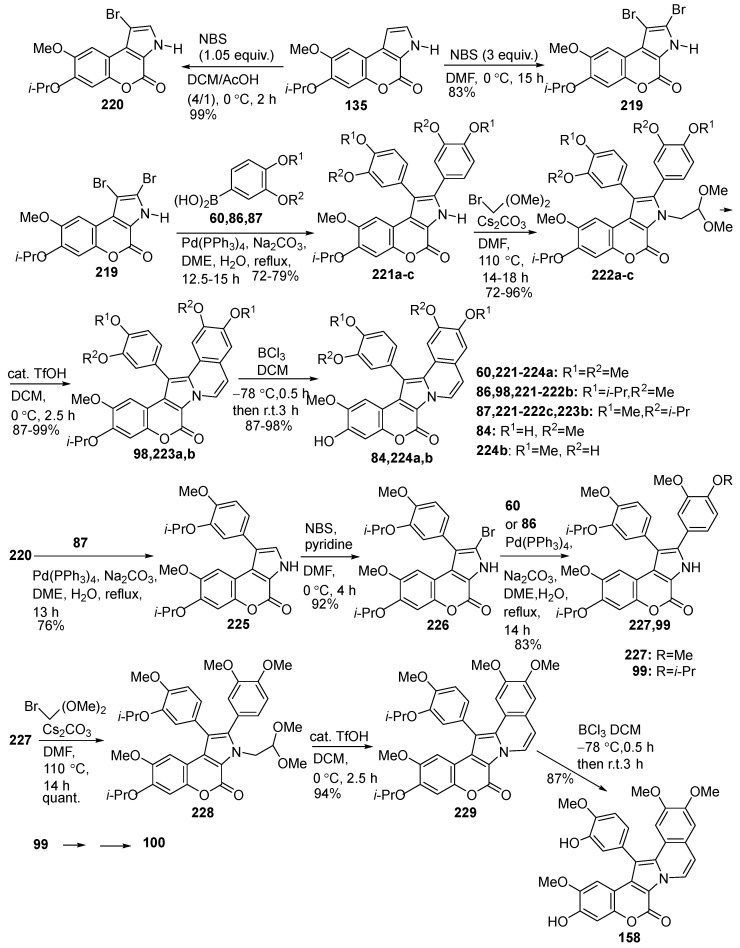
Synthesis of lamellarins η (**224a**), D (**84**), N (**100**), and α (**158**) and 5,6-dehydrolamellarin Y (**224b**).

In 2017, Fukuda et al. synthesized lamellarins η (**224a**) and D (**84**) and 5,6-dehydrolamellarin Y (**224b**) via a different method started from 7-isopropyl-8-methoxy-[1]benzopyrano[3,4-*b*]pyrrol-4(3*H*)-one (**135**), prepared from *N*-benzenesufonyl-1*H*-pyrrole (**131**) [92]. Bromination of **135** with excess NBS led to dibromo-derivative **219**, while, with one equivalent of NBS in DCM/AcOH (4:1), the monobromo-derivative **220** formed (Scheme 24). Suzuki cross-coupling reaction of **219** with boronic acids **60**, **86**, and **87** provided 2,3-diaryl-derivatives **221a**–**c**, which, upon *N*-alkylation with bromo-acetaldehyde dimethyl acetal, furnished compounds **222a**–**c**, respectively. TfOH-mediated cyclization of the latter afforded ethers **98** and **223a**,**b**. Selective deprotection of isopropyl group by BCl_3_ resulted in lamellarins D (**84**) and η (**224a**) and 5,6-dehydrolamellarin Y (**224b**) in 48%, 40%, and 56% overall yields, respectively [102]. Suzuki cross-coupling of **220** with boronic acid **87** provided 3-aryl derivative **225**, which upon bromination with NBS furnished bromo compound **226**. New Suzuki coupling with boronic acids **60** and **86** afforded ethers **227** and **99**, respectively. Compound **99** is the precursor for the synthesis of lamellarin N (**100**) [88]. Following the above-mentioned sequence by the *N*-alkylation of **227** with bromo-acetaldehyde dimethyl acetal, cyclization of **228** with TfOH, and selective hydrolysis of isopropyl group of **229** with BCl_3,_ the lamellarin α (**158**) was isolated in 47% total yield.

**Scheme 25:**
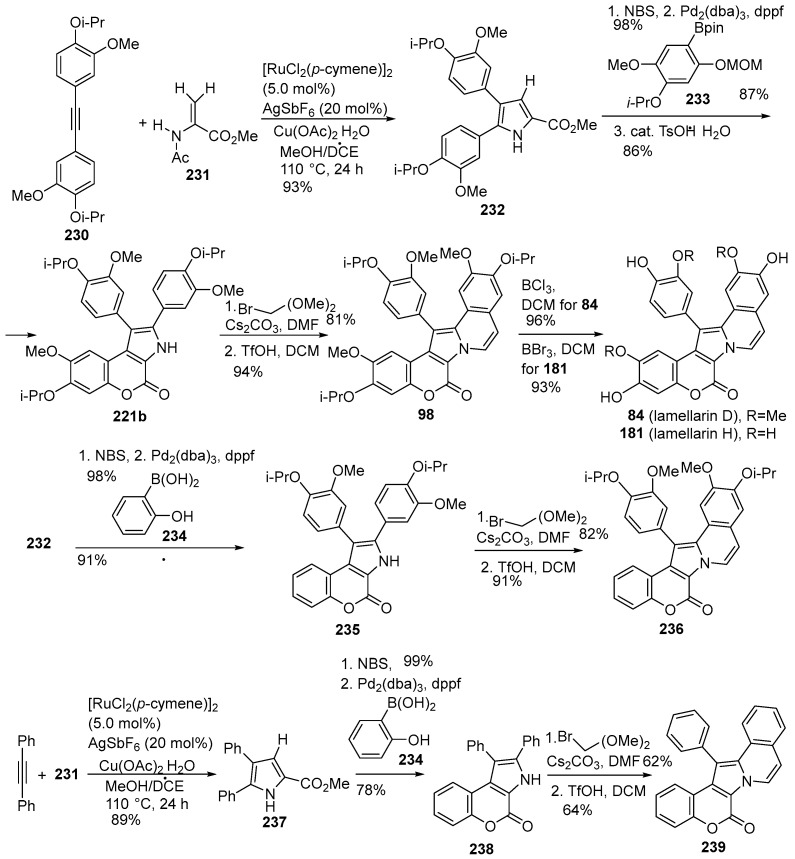
Synthesis of lamellarin D (**84**), lamellarin H (**181**), and lamellarin analogues **236** and **239**.

Ackermann and his colleagues presented the synthesis of lamellarins using the Ru-catalyzed C-H/N-H activation in an oxidation alkyne annulation process. So, the synthesis of pyrrole **232** was achieved from diaryl acetylene **230** and 2-aminoacrylate **231**. Pd-catalyzed annulated formation of lactone **221b** was obtained from **232** and boronate **233** in a one-pot bromination/Suzuki cross-coupling reaction procedure [103]. *N*-alkylation of compound **221b** with bromo-acetaldehyde dimethyl acetal followed by cyclization led to ether **98**, which by selective hydrolysis of isopropyl ether by BCl_3_ resulted in lamellarin D (**84**) in 55% total yield (Scheme 25). Deprotection of all ether groups in **98** by BBr_3_ afforded lamellarin H (**181**) in 54% overall yield. In this work, the lamellarin analogues **236** and **239** have been also prepared.

**Scheme 26:**
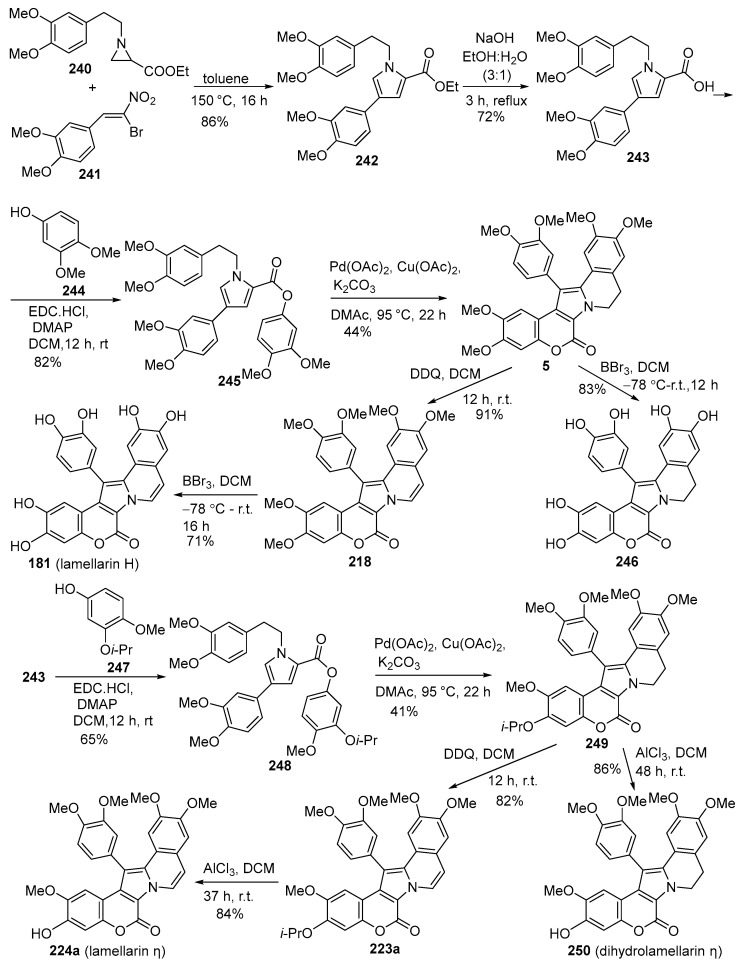
Synthesis of lamellarins H (**181**) and η (**224a**), dihydrolamellarin η (**250**), lamellarin G trimethyl ether (**5**), lamellarin D trimethyl ether (**218**), and tris-desmethyl lamellarin G (**246**).

**Scheme 27:**
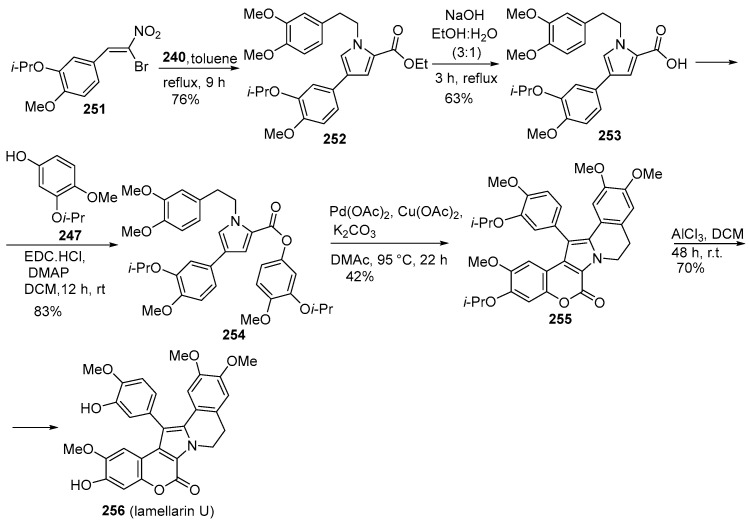
Synthesis of lamellarin U (**256**).

In 2019, Kumar et al., prepared 1,2,4-trisubstituted pyrrole as key precursor for the total synthesis of lamellarins [104]. Aziridine ester **240** reacted with β-bromo-β-nitrostyrene (**241**) in a one-pot [3 + 2] cycloaddition/elimination/aromatization reaction sequence to provide pyrrole ester **242**, which upon hydrolysis to **243** and esterification with phenol **244** led to the precursor 1,2,4-trisubstituted indole derivative **245** (Scheme 26). Double C-H oxidative coupling of the latter under Pd(OAc)_2_ catalysis in the presence of Cu(OAc)_2_ afforded lamellarin G trimethyl ether (**5**) in 22% total yield. Hydrolysis of **5** with BBr_3_ provided tris-desmethyl lamellarin G (**246**) in 18% total yield, while oxidation with DDQ furnished lamellarin D trimethyl ether (**218**) in 20% overall yield. Hydrolysis of **218** with BBr_3_ provided lamellarin H (**181**) in 14% total yield. Under analogous process, ester **248** led to dihydrolamellarin η (**250**) and lamellarin η (**224a**) in 14% and 11% total yields, respectively. By using β-nitrostyrene **251**, the above-mentioned reaction sequence resulted in the synthesis of lamellarin U (**256**) in 12% overall yield in five steps (Scheme 27).

**Scheme 28:**
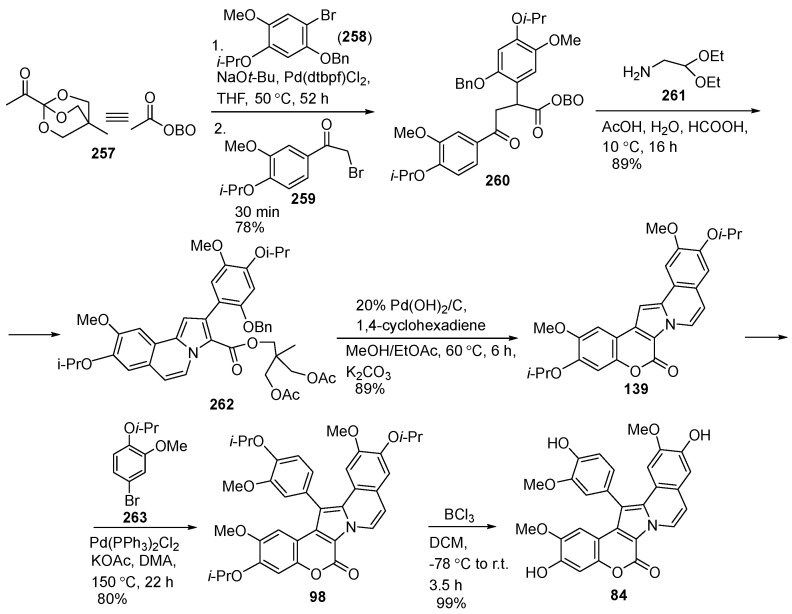
Synthesis of lamellarin D (**84**) using puruvic acid orthoester.

The same year, Shirley et al. demonstrated the synthesis of lamellarin D (**84**) in seven steps and 22% total yield from puruvic acid utilizing the puruvic acid orthoester **257** as building block for the synthesis of 1,4-dicarbonyl adduct **260**, by Pd-catalyzed arylation of **257** with bromoaryl derivative **258**, followed by enolate alkylation with α-bromoacetophenone **259** (Scheme 28). Treatment of **260** with amino acetaldehyde diethyl acetal (**261**) under reflux afforded the pyrrolisoquinoline compound **262**, which under transfer hydrogenation and lactonization furnished the fused pyrrolocoumarin product **139**. Pd-catalyzed C-H arylation of the latter with aryl bromide **263** led to triisopropyl lamelarine D (**98**), and finally selective deprotection with BCl_3_ resulted in lamellarin D (**84**) [105].

**Scheme 29:**
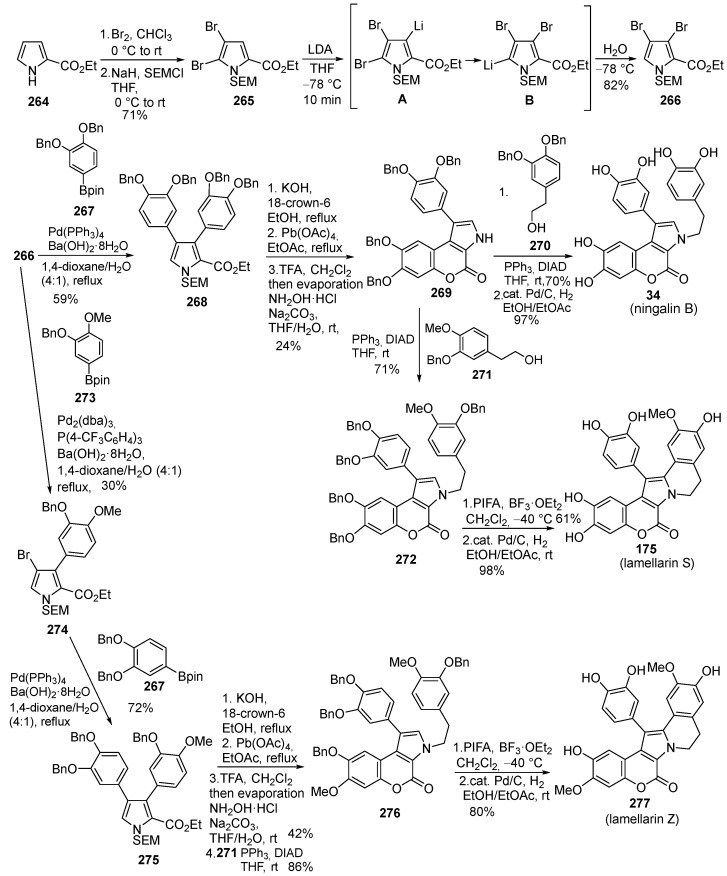
Synthesis of ningalin B (**34**) and lamellarins S (**175**) and Z (**277**).

Morikawa et al. reported the synthesis of ningalin B (**34**) and lamellarins S (**175**) and Z (**277**) using dibromopyrrolo derivative **266** as starting material [106]. Compound **266** was prepared by bromination of ethyl 1*H*-pyrrole-3-carboxylate (**264**) followed by protection of nitrogen with 2-(trimethylsilyl)ethoxymethyl (SEM) to provide **265** and subsequent treatment with LDA for the “halogen dance” via unstable intermediates **A** and **B** (Scheme 29). Suzuki–Miyaura cross-coupling reaction of **266** with boronate **267** afforded diarylated pyrrole ester **268**, which upon hydrolysis and Pb(OAc)_4_-mediated lactonization furnished pyrrolocoumarin derivative **269**. *N*-alkylation with **270** of the latter under Mitsunobu conditions resulted in ningalin B (**34**) in 10% yield over six steps from **266**. When the *N*-alkylation with arylethanol **271** was performed, compound **272** was formed, which under treatment with PIFA upon oxidative C-C formation and benzyl deprotection by Pd-catalyzed hydrogenation led to lamellarin S (**175**) in 6% yield and 7 steps from **266**. Regioselective Suzuki–Miyaura coupling of **266** with boronate **273** was provided under monoarylation pyrrole derivative **274**. Second Pd-catalyzed arylation of the latter with boronate **267** afforded diarylated pyrrole **275**. Similar treatment of the latter, like the above-mentioned compound **268**, resulted in the formation of lamellarin Z (**277**) in 6% yield over 8 steps from **266**.

**Scheme 30:**
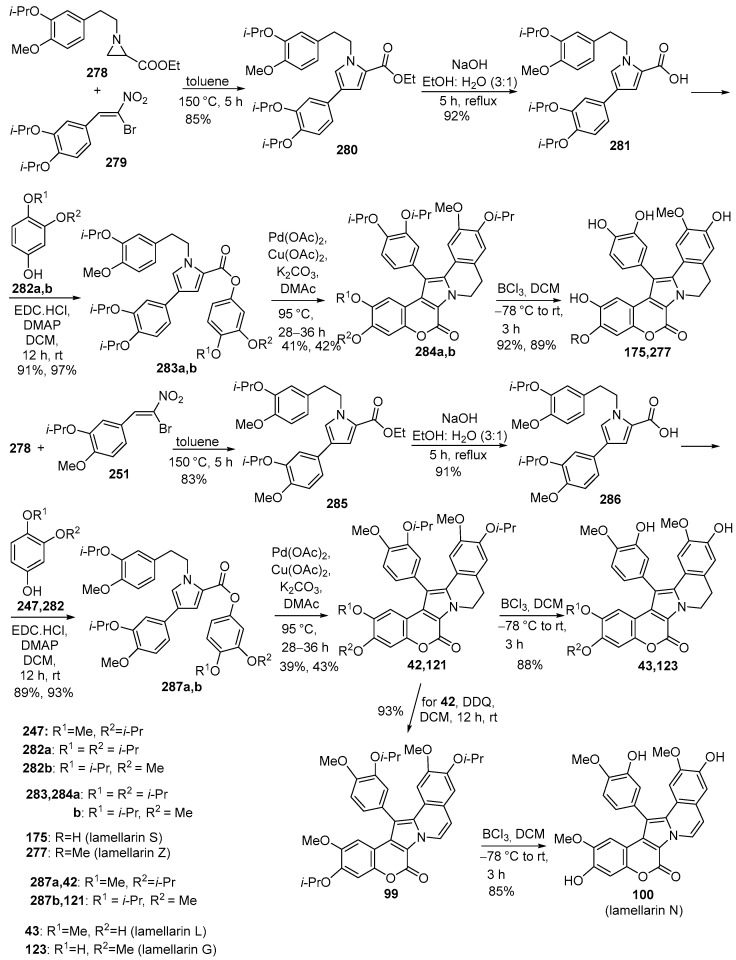
Total synthesis of lamellarins S (**175**), Z (**277**), L (**43**), G (**123**), and N (**100**).

Khan and coworkers presented [107] the total synthesis of lamellarins S (**175**), Z (**277**), L (**43**), G (**123**), N (**100**), and D (**84**) using the strategy of Scheme 26 and Scheme 27, applied for the synthesis of lamellarins H, η, and U. The one-pot [3 + 2] cycloaddition/elimination/aromatization domino reaction of aziridine ester **278** with β-bromo-β-nitrostyrene **279** afforded ester **280** (Scheme 30). Hydrolysis of the latter to **281**, esterification with phenols **281** or **282** to the esters **283a**,**b**, followed by Pd-catalyzed cross-dehydrogenative coupling to **284a**,**b**, and, finally, selective deprotection of isopropyl group resulted in lamellarins S (**175**) or Z (**277**) in 27% or 28% total yields, respectively, in 5 steps.

**Scheme 31:**
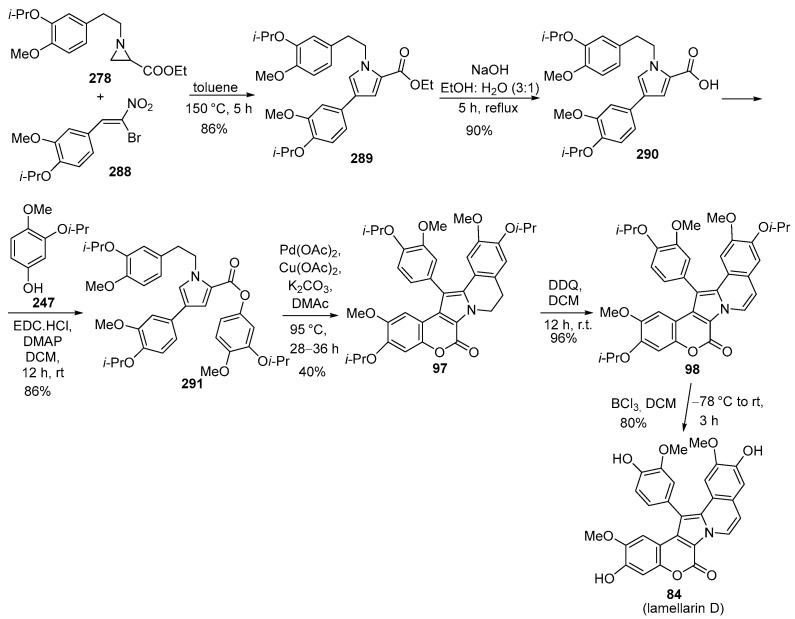
Synthesis of lamellarin D (**84**).

The analogous reaction sequence starting from compounds **278** and **251** led to lamellarins L (**43**) and G (**123**) in 27% and 23% total yields, respectively, in five steps. Oxidation of the intermediate **42** with DDQ afforded the triisopropyl ether of lamellarin N (**99**), which by selective deprotection by BCl_3_ furnished lamellarin N (**100**) in 24% total yield and 6 steps (Scheme 30). The similar reaction sequence started from derivatives aziridine ester **278** and β-bromo-β-nitrostyrene **288** resulted in the formation of lamellarin D (**84**) in 20% total yield in six steps (Scheme 31).

**Scheme 32:**
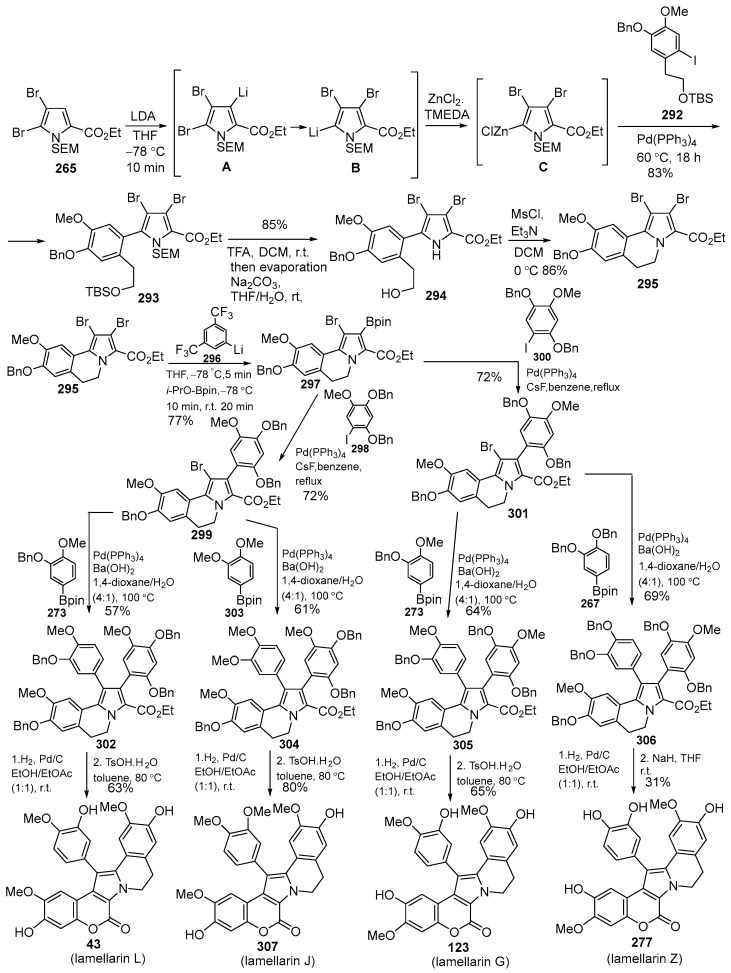
Total synthesis of lamellarins L (**43**), J (**307**), G (**123**), and Z (**277**).

In 2021, Okano and coworkers reported the total synthesis of lamellarins L (**43**), J (**307**), G (**123**), and Z (**277**) using a one-pot halogen dance/Negishi cross-coupling reaction of the α-lithiated dibromopyrrole intermediate **B** achieved from dibromopyrrole ester **265** (Scheme 32). Transmetallation of **B** to zinc-interrmediate **C** followed by coupling with iodide **292** afforded pentasubstituted pyrrole **293** [108]. Deprotection of the latter provided compound **294**, which by mesylation and cyclization led to fused dihydroisoquinoline derivative **295**. Borylation of **295** furnished the common intermediate for the synthesis of lamellarin **297**. Suzuki–Miyaura coupling of **297** with iodides **298** and **300** afforded bromopyrrole derivatives **299** and **301**, respectively. New Suzuki–Miyaura reaction of **299** and **301** with boronates **273** or **303** and **273** or **267** led to the fully arylated pyrrole derivatives **302** or **304** and **305** or **306**. Hydrogenolysis of benzyl ethers **302**, **304**, **305**, and acidic lactonization resulted in the formation of lamellarins L (**43**), J (**307**), and G (**123**), in 12%, 16%, and 14% total yields in 8 steps, respectively. After the hydrogenolysis of **306**, basic lactonization with NaH was necessary for the synthesis of lamellarin Z (**277**) in 7% total yield.

**Scheme 33:**
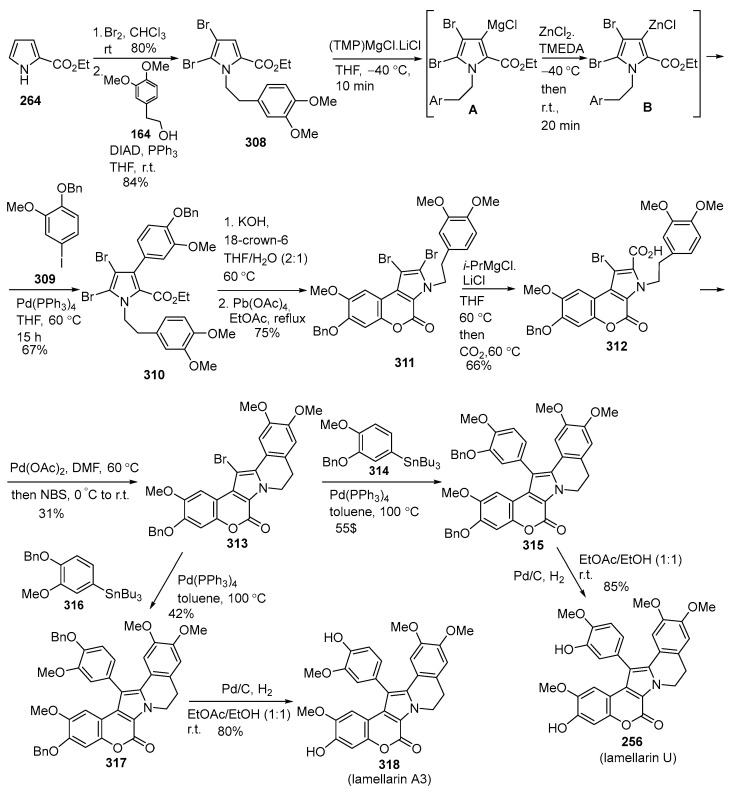
Total synthesis of lamellarins U (**256**) and A3 (**318**).

The same group [109] described the total synthesis of lamellarins U (**256**) and A3 (**318**) by interrupting the halogen dance of the metalated α,β-dibromopyrrole derivative (Scheme 33). Deprotonative metalation of pyrrole derivative **308** with (TMP)MgCl.LiCl (TMP = 2,2,6,6-tetramethylpiperidide) to intermediate **A** followed by transmetalation with ZnCl_2_.TMEDA furnished Zn-intermediate **B**, which did not promote halogen dance reaction. Negishi cross-coupling reaction of the latter with iodide **309** provided 4,5-dibromopyrrole ester **310** (Scheme 33). Hydrolysis and oxidative cyclization of **310** afforded [3,4]-fused pyrrolocoumarin **311**, which by α-selective bromo-magnesium exchange and reaction with CO_2_ afforded acid **312**. By Pd-mediated cyclization of **312**, pentacyclic derivative **313** achieved. Kosugi–Migita–Stille cross-coupling reaction of the latter with stannous compounds **314** or **316** led to aryl derivatives **315** or **317**, which by subsequent hydrogenolysis resulted in lamellarins U (**256**) and A3 (**318**) in 3% and 2% total yields in nine steps, respectively.

#### 2.1.2. Synthesis Using Isoquinoline Derivatives

**Scheme 34:**
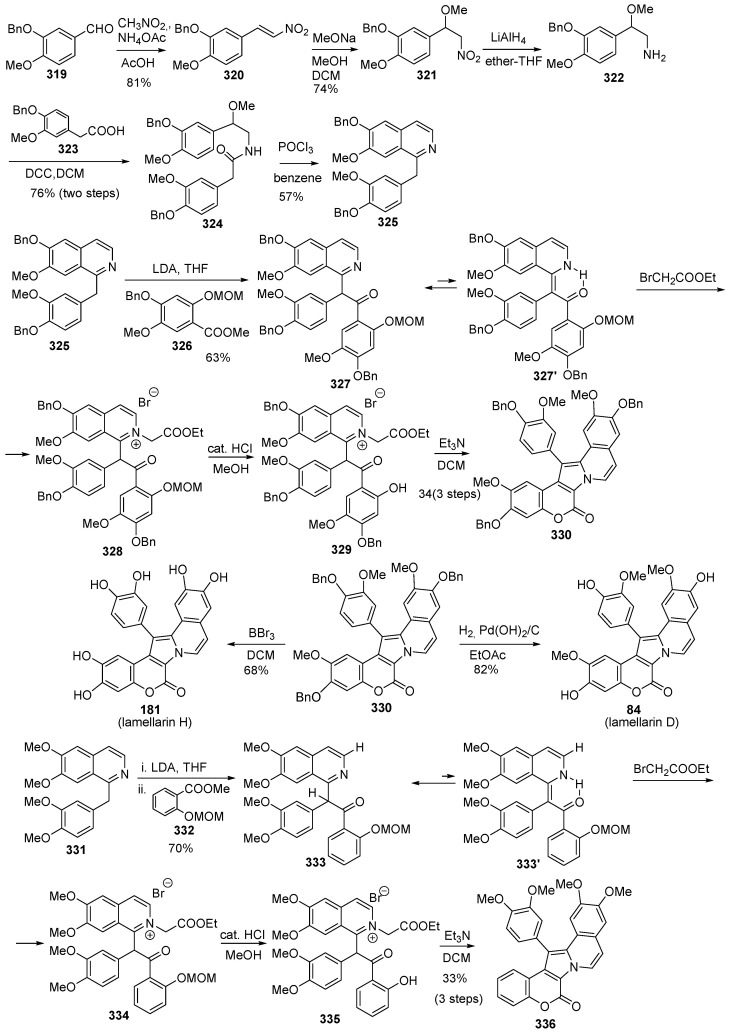
Synthesis of lamellarins D (**84**) and H (**181**) and lamellarin analogue **336**.

In 1997, Ishibashi and coworkers reported the total synthesis of lamellarins D (**84**) and H (**181**) as the first synthesis of lamellarin alkaloids [110]. The construction of pentacyclic ring of lamellarins was achieved by the *N*-ylide-mediated pyrrole formation from **329** followed by lactonization to provide ether **330**. Hydrogenolysis of benzyl group of compound **330** led to lamellarin D (**84**) in 18% yield in 5 steps from **325**, while deprotection under treatment with BBr_3_ resulted in lamellarin H (**181**) in 15% in 5 steps from **325** (Scheme 34). Isoquinolinium salt **329** was synthesized starting from benzylisoquinoline **325** (prepared from benzaldehyde **319** in 26% yield in 5 steps) under lithiation with LDA and reaction with benzoate **326** to provide the tautomeric mixture of acylated isoquinolines **327** and **327′**. Reaction of the latter with ethyl bromoacetate provided protected quinolinium salt **328**, which under deprotection with methanolic HCl afforded salt **329**. In a similar reaction sequence starting from papaverine (**331**), have synthesized the lamellarin analogue **336** with 33% yield in 3 steps.

**Scheme 35:**
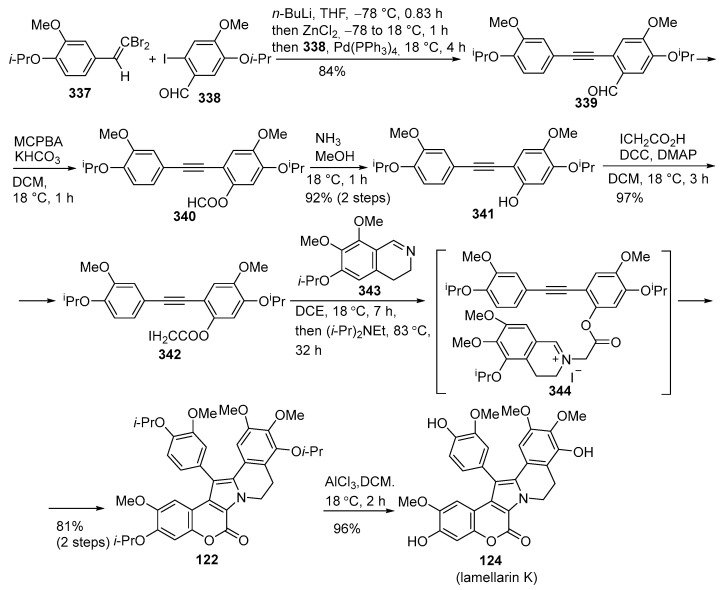
Total synthesis of lamellarin K (**124**).

The same year, Banwell et al. presented the total synthesis of lamellarin K (**124**) through an intramolecular [3 + 2] cycloaddition reaction of azomethine ylide, in situ formed from the isoquinolinium salt **344** in the presence of Hunig’s base (diisopropylethylamine), followed by aromatization to provide lamellarin K triisopropyl ether **122** (Scheme 35). Selective de-isopropylation of the latter with AlCl_3_ resulted in lamellarin K (**124**) [111]. The intermediate salt **344** was achieved by nucleophillic substitution of iodoacetate **342** by isoquinoline **343**. Iodoacetate ester **342** was obtained by the reaction sequence starting from the reaction of styrene **337** with iodide **338** via treatment of **337** with *n*-BuLi, transmetallation of lithium acetylide formed with ZnCl_2_, and Pd-mediated coupling of the resulting zinc acetylide with iodide **338** to provide alkyne derivative **339**. Baeyer–Villiger oxidation of **339** afforded formate ester **340**, which by hydrolysis to phenol **341** and esterification with iodoacetic acid furnished iodoacetate ester **342**.

**Scheme 36:**
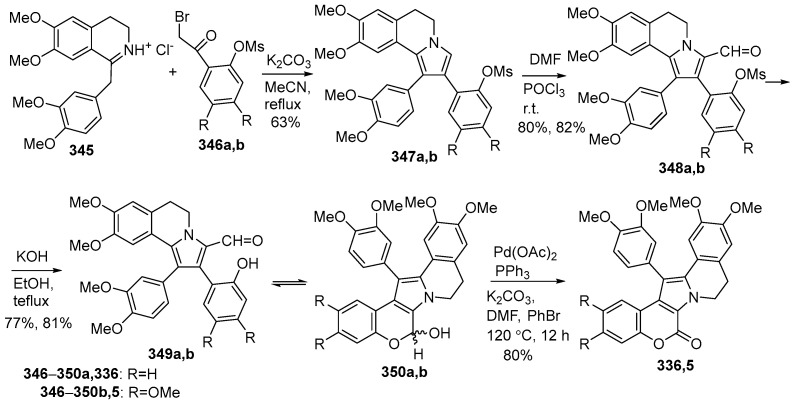
Total synthesis of lamellarin G trimethyl ether (**5**).

Ruchirawat and Mutarapat reported the total synthesis of lamellarin G trimethyl ether (**5**) starting from the condensation of dihydroisoquinolinium salt **345** and phenacyl bromide **346b** under basic conditions for the synthesis of dihydropyrrolo[2,1-*a*]isoquinoline **347b** [112]. Vilsmeier reaction of the latter led to the introduction of formyl group on pyrrole ring to provide adduct **348b** (Scheme 36). After deprotection and oxidation of the resulting hydroxypyran derivative **350b** in the presence of bromobenzene, Pd(OAc)_2_, PPh_3_, and K_2_CO_3_ in DMF, the lamellarin G trimethyl ether (**5**) was synthesized in 33% total yield.

**Scheme 37:**
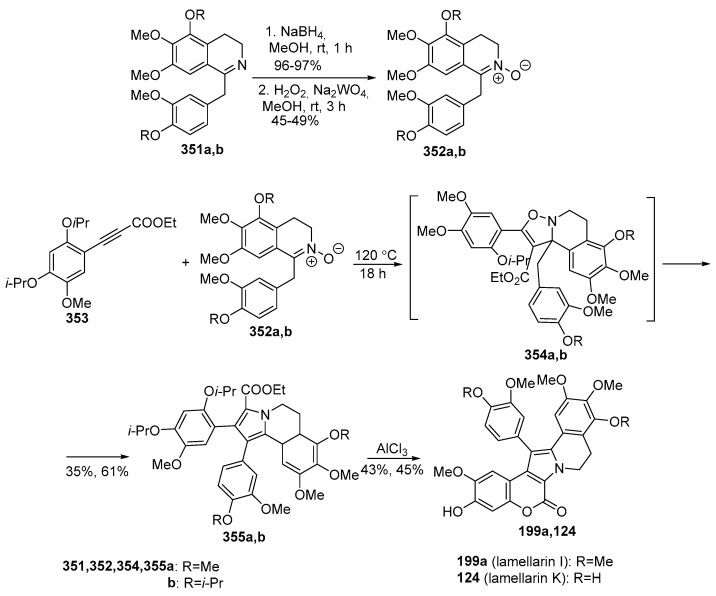
Synthesis of lamellarins I (**199a**) and K (**124**).

Diaz et al. synthesized lamellarins I (**199a**) and K (**124**) by the 1,3-cycloaddition reactions of nitrones **352a**,**b** with alkyne **353** [113]. The intermediate isoxazolines **354a**,**b**, bearing a benzyl substituent at 3 position, were rearranged to pyrrole derivatives **355a**,**b**, which by deprotection of isopropyl group with AlCl_3_ and lactonization resulted in lamellarins I (**199a**) and K (**124**) in 15%, and 27% yields, respectively (Scheme 37).

**Scheme 38:**
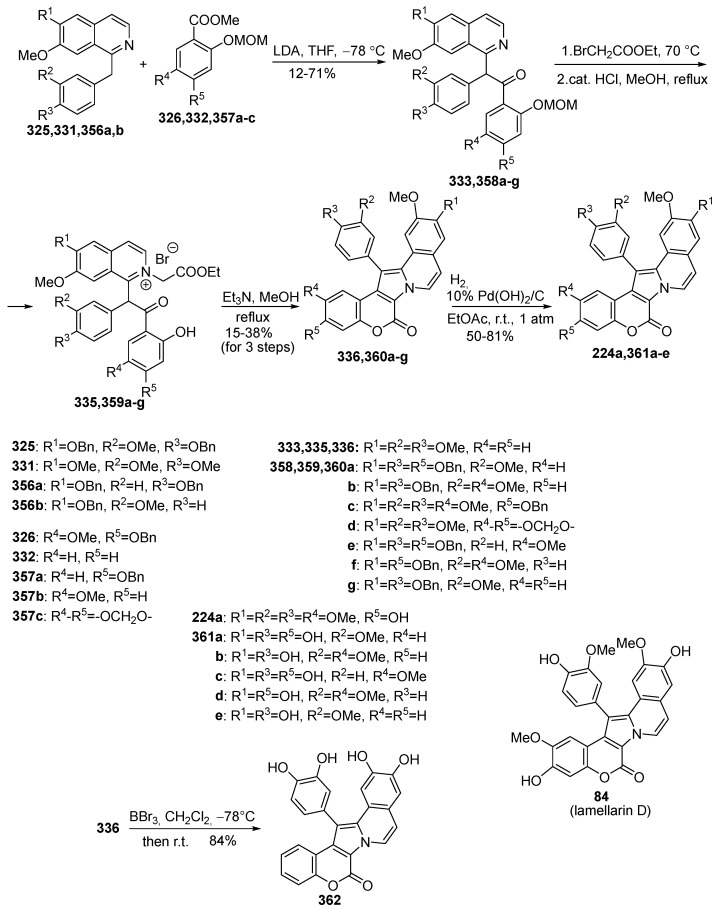
Synthesis of lamellarin D analogues.

Ishibashi et al. presented the synthesis of lamellarin D derivatives **224a**, **336**, **360a**–**g**, **361a**–**e**, and **362** and their evaluation for cytotoxicity against HeLa cell lines [114]. This synthesis started from the condensation of benzyl isoquinolines **325**, **330**, and **356a**,**b** with benzoates **326**, **331**, and **357a**–**c** in the presence of LDA to provide adducts **332** and **358a**–**g** (Scheme 38). *N*-alkylation of the latter with ethyl bromoacetate followed by hydrolysis of OMOM-protecting group afforded quaternary ammonium salts **335** and **359a**–**g**. Treatment with Et_3_N furnished intermediate *N*-ylide, which cyclized intramolecularly to form pyrrole ring with simultaneous aromatization and lactonization, which led to lamellarin derivatives **336** and **360a**–**g**. Hydrogenolysis of benzyl ethers resulted in products **224a** and **361a**–**e**. Deprotection of compound **336** with BBr_3_ afforded tetrahydroxy derivative **362**.

Compounds **84**, **361a**, **361c**, and **361d** showed high activity with IC_50_ values in the range 10.5–70.0 nM during the cytotoxicity test against HeLa cell lines. The authors examined the structure–activity relationships of all synthesized compounds and found that hydroxy groups at C-8 and C-20 positions were important for their cytotoxic activity.

**Scheme 39:**
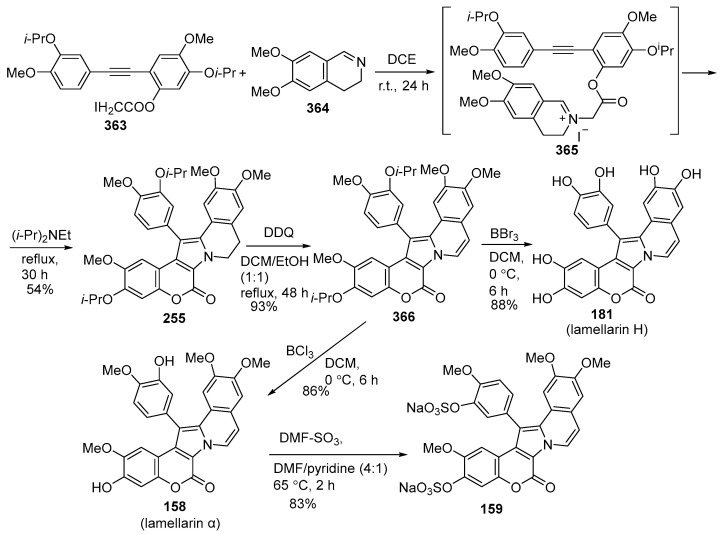
Synthesis of lamellarin H (**181**), lamellarin α (**158**), and lamellarin α 13,20-disulfate (**159**).

Faulkner and coworkers [115] reported the synthesis of lamellarin H (**181**), lamellarin α (**158**), and lamellarin α 13,20-disulfate (**159**) following the above-mentioned procedure of Banwell et al. with intramolecular [3 + 2] cycloaddition reaction of azomethine ylide to alkyne moiety (Scheme 35). Azomethine ylide was formed in situ from the quaternary ammonium salt **365**, achieved by coupling of iodoacetate **363** with dihydroisoquinoline **364**, after treatment with diisopropyl ethyl amine (DIPEA and Hunig’s base), and via the [3 + 2] cycloaddition reaction resulted in the [3,4]-fused pyrrolocoumarin derivative **255** (Scheme 39). Oxidation with DDQ afforded the intermediate lamellarin derivative **366**, which was the precursor for the synthesis of lamellarin H (**181**) and lamellarin α (**158**), after the deprotection with BBr_3_ or the selective deprotection with BCl_3_, respectively, in 15% overall yield. Treatment of compound **158** with a DMF complex of SO_3_ furnished lamellarin α 13,20-disulfate (**159**).

Compounds **158**, **159**, and **181** have been evaluated for their biological activities against HIV-1 integrase and MCV topoisomerase and for their cytotoxicity against HeLa cell lines. Lamellarin H (**181**) exhibited very potent inhibition of HIV-1 integrase with IC_50_ = 1.3 μM, better than lamellarin α 20-sulfate, which is a selective inhibitor of HIV-1 integrase (IC_50_ = 22 μM) but is also active in the MCV topoisomerase with IC_50_ = 0.23 μM having no specificity, as required to be attractive for drug development. Compound **181** was quite cytotoxic against HeLa cell lines with LD_50_ = 5.7 μM.

**Scheme 40:**
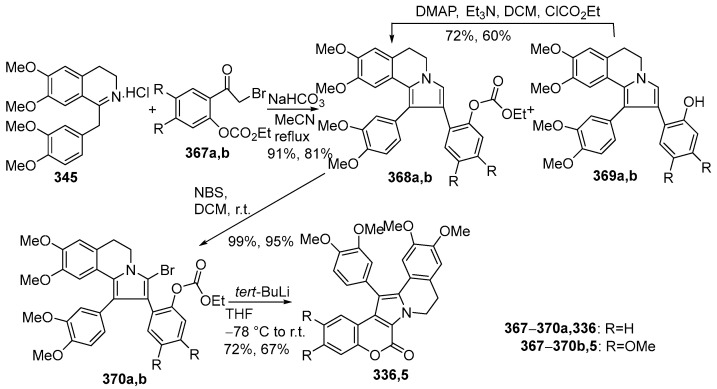
Synthesis of lamellarin alkaloids via metal–halogen exchange.

Ruchirawat and coworkers improved their former synthesis of lamellarin skeleton (Scheme 36) using as key step the direct remote metalation (DReM) or the metal–halogen exchange in intermediate fused pyrroloisoquinoline derivatives **368a**,**b**, bearing an *ortho*-carbonate substituent as a directing group in the 3-aryl pyrrole ring [116]. Intermediates **368a**,**b** achieved by the reaction of benzyl dihydroisoquinoline derivative **345** with carbonates **367a**,**b** along with phenol adducts **369a**,**b** as an inseparable mixture under treatment with DMAP, Et_3_N, and ethyl chloroformate afforded only carbonates **368a**,**b** (Scheme 40). Bromination of the latter with NBS to provide bromides **370a**,**b**, followed by treatment with *tert*-BuLi for the lithium–bromine exchange, and lactonization resulted in the formation of lamellarin analogues **336** and **5** in 72% and 67% yields, respectively.

**Scheme 41:**
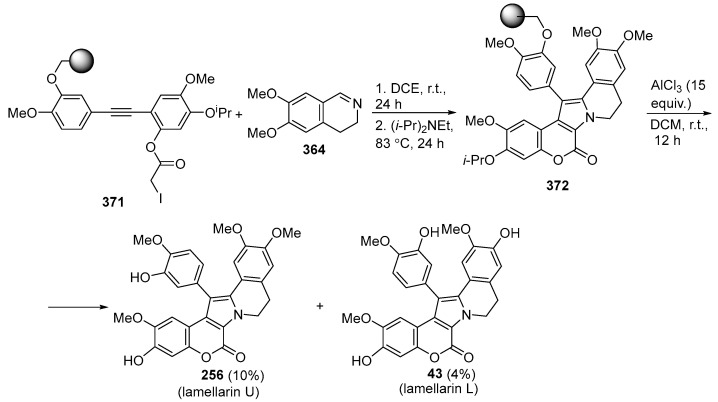
Synthesis of lamellarins U (**256**) and L (**43**).

Cironi et al. utilized a solid-phase synthesis using the Merrifield resin for the total synthesis of lamellarins U (**256**) and L (**43**) [117]. *N*-alkylation of dihydroisoquinoline derivative **364** with resin iodoacetate adduct **371** followed by [3 + 2] cycloaddition reaction of the azomethine ylide formed in situ after the treatment with DIPEA, in analogy to that depicted in Scheme 39, afforded the lamellarin framework **372** connected to the Merrifield-type resin (Scheme 41). The cleavage of the latter with excess of AlCl_3_ resulted in a crude product, which after separation with semipreparative HPLC furnished lamellarins U (**256**) and L (**43**) in 10% and 4% overall yields over 8-step solid-supported synthesis.

**Scheme 42:**
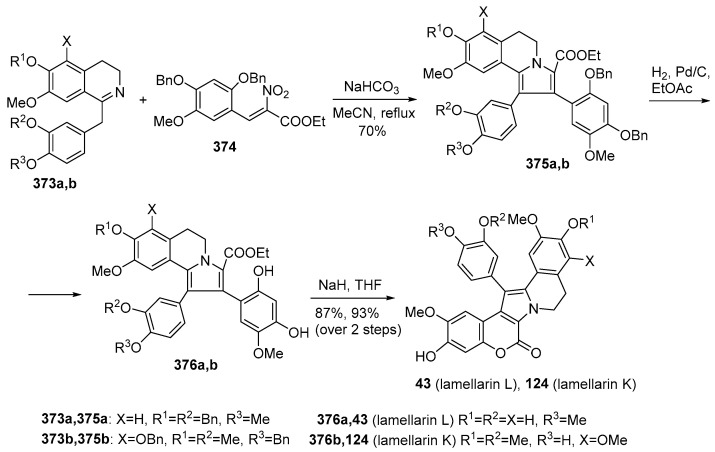
Synthesis of lamellarins L (**43**) and K (**124**).

Ruchirawat and coworkers presented another synthesis of lamellarins L (**43**) and K (**124**) in three steps from benzyl dihydroisoquinolines **373a**,**b** and ethoxycarbonyl-β-nitrostyrene **374** in 61% and 65% overall yields, respectively [118]. The key step of this reaction is the Michael addition of **373a**,**b** to **374** in the presence of NaHCO_3_ followed by ring closure to form the fused pyrrole ring of **375a**,**b** (Scheme 42). Hydrogenolysis of the latter afforded the hydroxy-substituted derivatives **376a**,**b**, which by lactonization under basic conditions resulted in the synthesis of lamellarins L (**43**) and K (**124**).

**Scheme 43:**
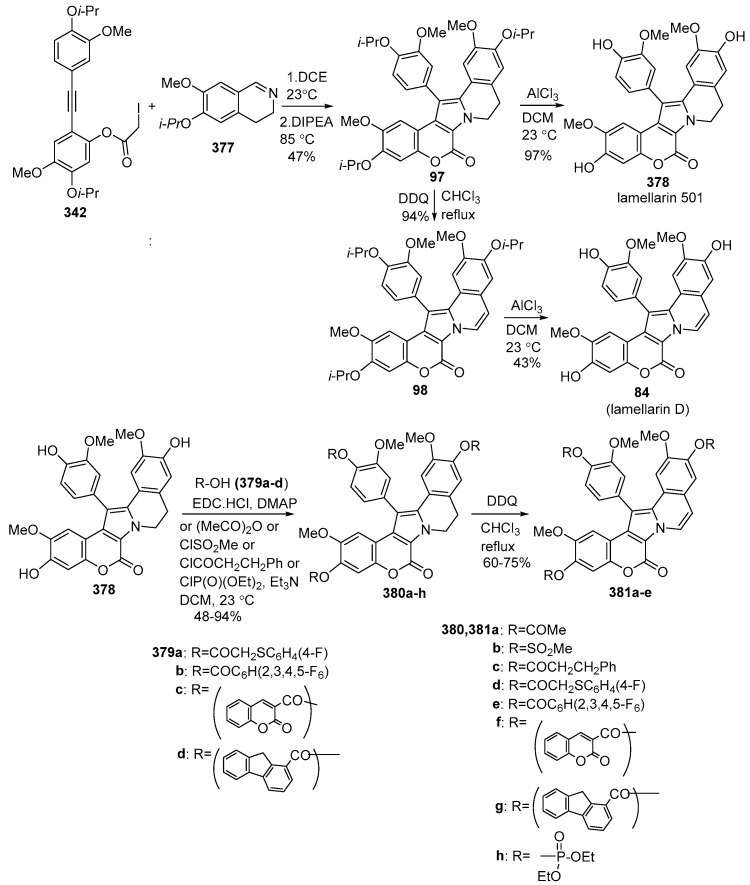
Synthesis of lamellarin D **(84**) and lamellarin 501 (**378**) derivatives.

**Scheme 44:**
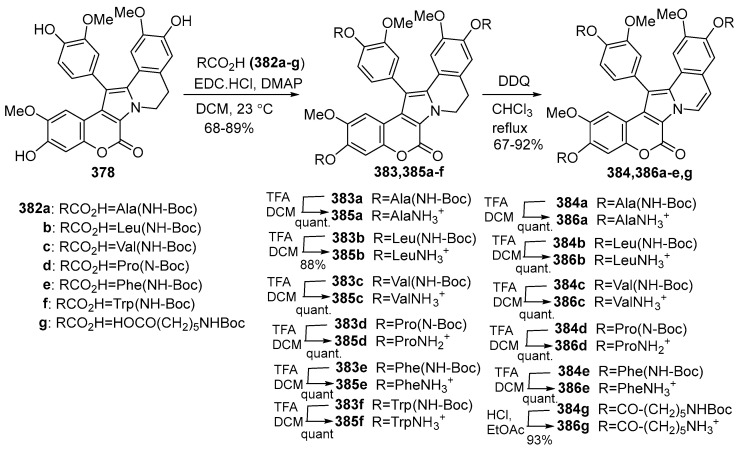
Synthesis of lamellarin D triester derivatives with aminoacids.

In 2004, Bailly and coworkers used the procedure described by Banwell for the synthesis of lamellarins [110] (Scheme 35) to achieve derivatives of lamellarin D and of the dihydro adduct lamellarin-501. This work aimed to study their structure–activity relationship as lamellarin D is an antitumor agent targeting topoisomerase I [119]. Coupling of dihydroisoquinoline derivative **377** with iodoacetate **342**, followed by azomethine ylide formation in the presence of DIPEA, intramolecular [3 + 2] cycloaddition reaction of azomethine ylide to triple bond, aromatization of pyrrole ring, and lactonization afforded lamellarin D triisopropyl ester **97**, having saturated the 5,6-double bond (Scheme 43). Oxidation of the latter furnished the lamellarin D triisopropyl ether (**98**), and selective hydrolysis of isopropyl group with AlCl_3_ resulted in the synthesis of lamellari D (**84**). The lamellarin 501 (**378**), with a saturated 5,6-double bond derivative of lamellarin D, was achieved by analogous hydrolysis of compound **97**. Lamellarin 501 (**378**) was the starting compound for the synthesis of triesters **380a**–**h** after the treatment with acids in the presence of EDC.HCl or from the acid chlorides in the presence of Et_3_N. Oxidation of the latter with DDQ afforded lamellarin D triesters **381a**–**e**. *N*-Protected aminoacid derivatives **383a**–**f** and **384a**–**e**,**g** of lamellarins 501 and D were achieved by similar reactions of the starting compound **378** (Scheme 44). Deprotection of aminoacids with trifluoroacetic acid (TFA) resulted in the cationic derivatives **385a**–**f** and **386a**–**e**,**g**, respectively.

Compounds **380a**–**h** and **381a**–**e** were evaluated as topoisomerase I inhibitors and showed no activity, whilst they were non-cytotoxic. Compounds **386a**–**e**,**g** strongly promoted DNA cleavage by human topoisomerase I. The Boc-protected compounds **384a**–**e**,**g** and the analogue derivatives **383a**–**f** without the 5,6-double bond showed no effect on topoisomerase I, and they were less cytotoxic than cationic compounds **386a**–**e**,**g**. These aminoacid derivatives were found to be good antitumor agents targeting topoisomerase I. Compounds **386c** and **386d** with GI_50_ = 10.8 nM and 18.6 nM, respectively, have been selected for preclinical tests for their antitumor activity in vivo.

**Scheme 45:**
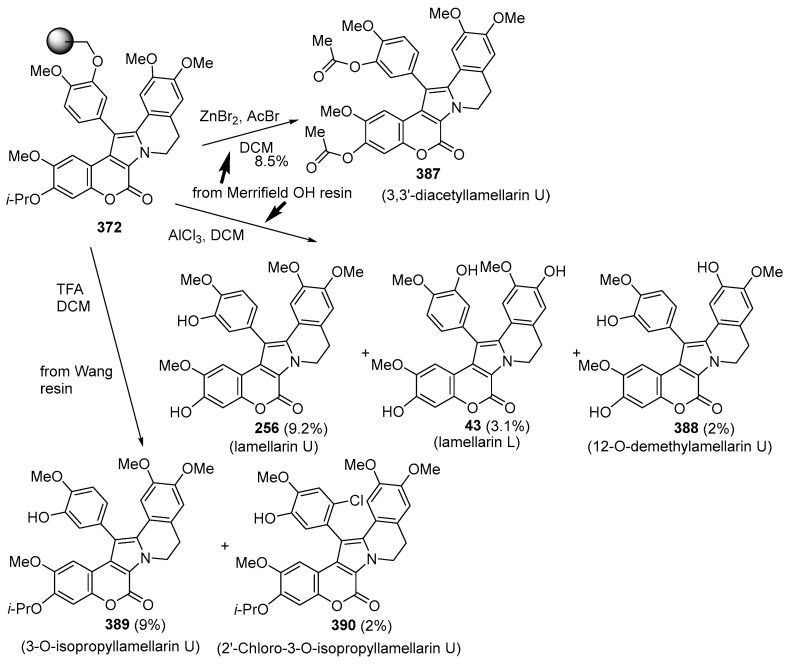
Synthesis of lamellarin U (**256**) and its derivatives, and lamellarin L (**43**) by solid-phase procedure.

Cironi et al. utilized their former solid-phase synthesis (Scheme 41) to synthesize lamellarin derivatives using different Lewis acids for the cleavage of resins [120]. From the intermediate lamellarin derivative **372**, prepared from Merrifield OH resin or Wang resin, 3,3′-diacetyllamellarin U (**387**) was achieved by treatment with ZnBr_2_ in the presence of acetylbromide in 8.5% overall yield (Scheme 45). When AlCl_3_ was used with the same derivative, **372** lamellarin U (**256**), lamellarin L (**43**), and 12-O-demethoxylamellarin U (**388**) were obtained in 9.2%, 2.0%, and 3.1% overall yields, respectively. The use of TFA with intermediate **372**, prepared from Wang resin, resulted in the synthesis of 3-*O*-isopropyllamellarin U (**389**) and 2′-chloro-3-isopropyllamellarin (**390**) in 9% and 2% overall yields, respectively.

**Scheme 46:**
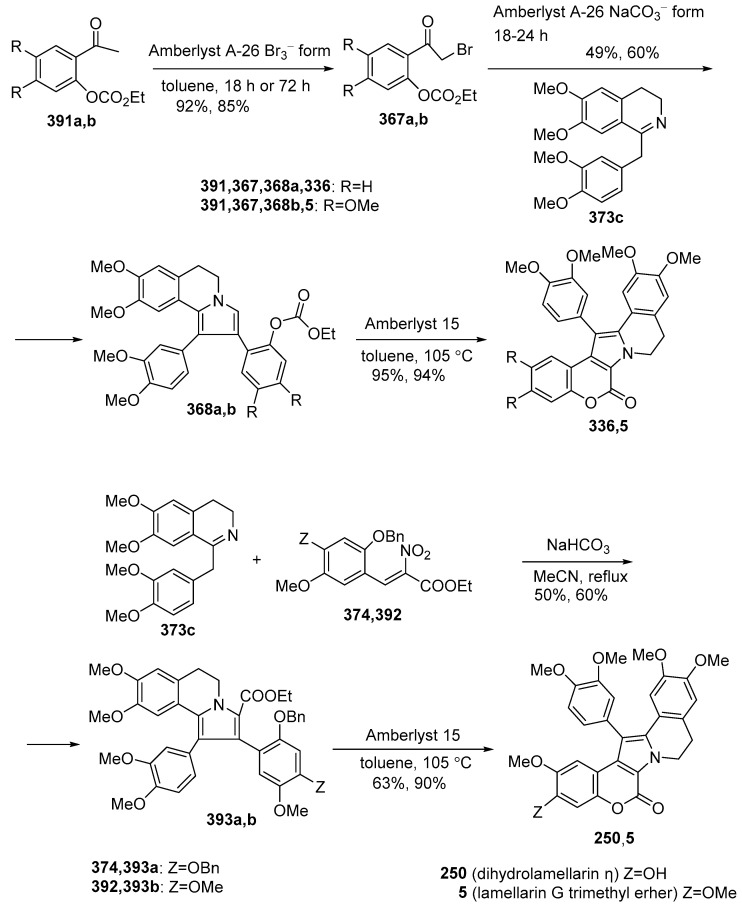
Synthesis of lamellarins by polymer-supported reagents.

Ruchirawat and coworkers presented the polymer-supported synthesis of lamellarins starting from phenacyl bromide derivatives or α-nitrocinnamate derivatives [121]. Bromination of substituted acetophenones **391a**,**b** by the polymer-supported Amberlyst A-26 Br_3_^-^ form afforded phenacyl bromide **367a**,**b** (Scheme 46). Coupling of the latter with dihydroisoquinoline derivative **373c** in the presence of Amberlyst A-26 NaCO_3_^−^ as base furnished the pyrrole derivatives **368a**,**b**. Intramolecular Friedel–Crafts transacylation from the carbonate carbonyl group to 2 position of pyrrole ring in the presence of acidic Amberlyst 15 followed by lactonization resulted in lamellarin derivatives **336** and **5**. In an alternative procedure, Michael addition of **330** to α-nitrocinnamates **374**,**392** under basic conditions followed by ring closure afforded pyrrole derivatives **393a**,**b** (Scheme 46). Deprotection of benzyl group with Amberlyst 15 followed by lactonization resulted in the formation of dihydrolamellarin η (**250**) and lamellarin G trimethyl ether (**5**) in 63% and 90% yields for the last step.

**Scheme 47:**
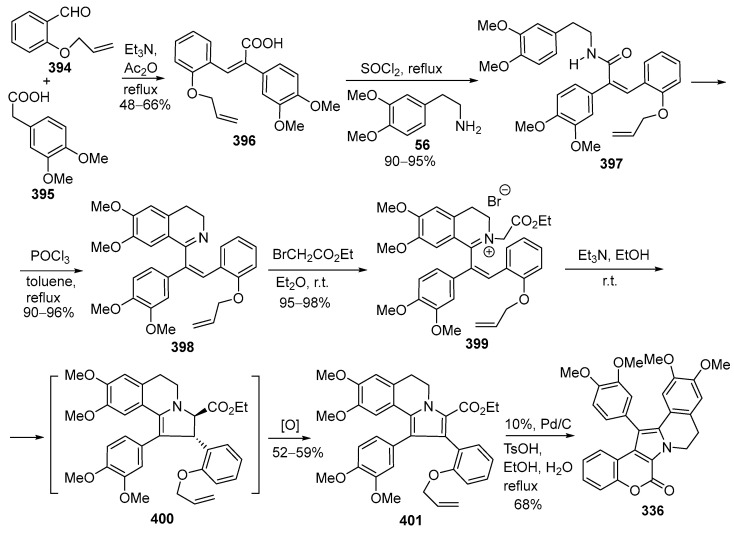
Synthesis of lamellarin skeleton by 1,5-electrocyclization of azomethine ylides.

Nyerges and Toke utilized the 1,5-electrocyclization of azomethine ylides leading to dihydropyrrolo[2,1-*a*]isoquinolines for the construction of lamellarin moiety [122]. The quaternary salt **399**, precursor of azomethine ylide, was achieved from *o*-allyloxy stilbenic acid **396** under amide **397** formation by the reaction with aryl ethyl amine **56** (Scheme 47). Condensation of amide **397** under treatment with POCl_3_ afforded dihydroisoquinoline derivative **398**, which after coupling with ethyl bromoacetate furnished salt **399**. Treatment of the latter with Et_3_N led in situ to azomethine ylide, which after intramolecular 1,3-dipolar cycloaddition reaction with 1,5-electrocyclization afforded dihydropyrrole intermediate **400**, and by aerobic oxidation resulted in the pyrrole derivative **401**. Deprotection of the allyloxy group by 10% Pd/C in the presence of TsOH followed by lactonization led to the formation of lamellarin skeleton of **336** in 21% total yield.

**Scheme 48:**
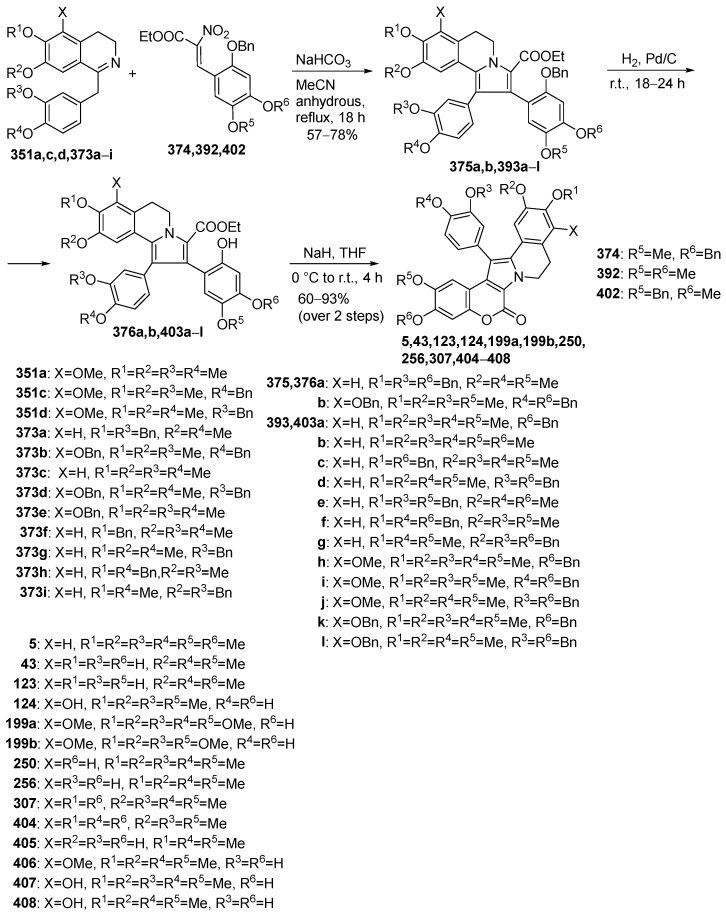
Synthesis of lamellarins G trimethyl ether (**5**), L (**43**), G (**123**), K (**124**), I (**199a**), C (**199b**), dihydro η (**250**), U (**256**), J (**307**), X (**404**), Y (**405**), T (**406**), F (**407**), and E (**408**).

**Scheme 49:**
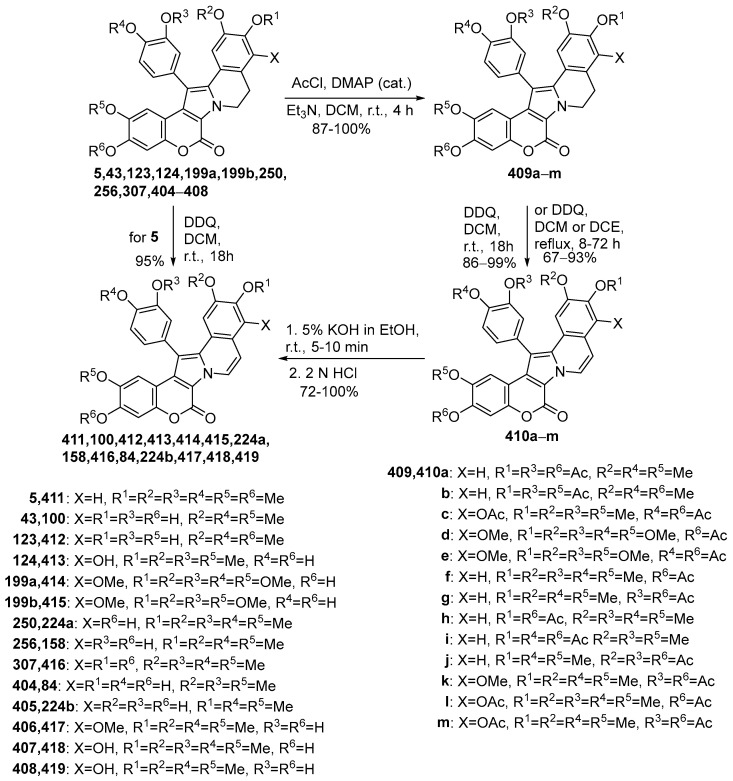
Synthesis of lamellarins D (**84**), N (**100**), α (**158**), η (**224a**), 5,6-dehydro Y (**224b**), 5,6-dehydro G trimethyl ether (**411**), 5,6-dehydro G (**412**), M (**413**), ζ (**414**), B (**415**), 5,6-dehydro J (**416**), W (**417**), ε (**418**), and X (**419**).

Ploypradith et al. synthesized 28 natural and synthetic lamellarins using their former procedure (Scheme 42) with benzyl dihydroisoquinolines **351a**,**c**,**d** and **373a**–**i** and *α*-nitrocinnamates **374**, **392**, and **402**. Michael addition of **351a**,**c**,**d**, **373a**–**i** to β-nitro styrenes **374**, **392**, and **402** in the presence of NaHCO_3_ followed by ring closure afforded the fused pyrrole rings of **375a**,**b** and **393a**–**l** [123]. Hydrogenolysis of the latter furnished hydroxy-substituted derivatives **376a**,**b** and **403a**–**l**, which, by lactonization under basic conditions, resulted in the synthesis of lamellarins G trimethyl ether (**5**), L (**43**), G (**123**), K (**124**), I (**199a**), C (**199b**), dihydro η (**250**), U (**256**), J (**307**), X (**404**), Y (**405**), T (**406**), F (**407**), and E (**408**) in 45-71% overall yields in three steps (Scheme 48). For the synthesis of 5,6-dehydrated derivatives of the above-mentioned lamellarins, the derivatives L (**43**), G (**123**), K (**124**), I (**199a**), C (**199b**), dihydro η (**250**), U (**256**), J (**307**), X (**404**), Y (**405**), T (**406**), F (**407**), and E (**408**) were acetylated with acetyl chloride in the presence of DMAP and Et_3_N to provide acetylated derivatives **409a**–**m** (Scheme 49). Oxidation of the latter by DDQ afforded deacetylatived derivatives **410a**–**m**, which upon oxidation with DDQ resulted in the synthesis of lamellarins D (**84**), N (**100**), α (**158**), η (**224a**), 5,6-dehydro Y (**224b**), 5,6-dehydro G (**412**), M (**413**), ζ (**414**), B (**415**), 5,6-dehydro J (**416**), W (**417**), ε (**418**), and X (**419**) in 54–95% overall yields. The total yield over 6 steps was 23–63%. Similar oxidation of lamellarins G trimethyl ether (**5**) furnished 5,6-dehydro G trimethyl ether (**411**) in 95% yield.

Ploypradith and coworkers examined the above synthesized lamellarin derivatives for their cytotoxicities against cancer cell lines of lung, breast, liver, oral, cervix, and blood cell [124]. Lamellarins D (**84**), X (**419**), K (**124**), M (**413**), N (**100**), ε (**418**), and dehydrolamellarin J (**416**) exhibited more potent anticancer activities with IC_50_ in nanomolar or low micromolar ranges than the positive control etoposide in most cancer cell lines. The results of the SAR studies revealed the contribution of the C5=C6 double bond as well as the hydroxy group at the C-8 and C-20 positions toward the anticancer activity of the lamellarin derivatives.

**Scheme 50:**
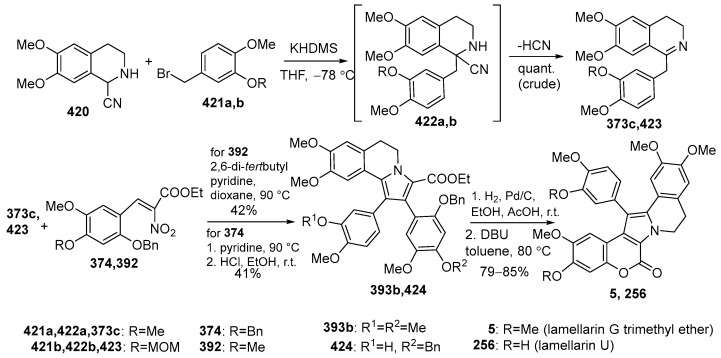
Synthesis of lamellarin U (**256**) and lamellarin G trimethyl ether (**5**).

Liermann and Opatz used 1,2,3,4-tetrahydroisoquinoline-1-carbonitrile **420** for the preparation of 1-benzyl-3,4-dihydroisoquinolines **373c** and **423** for the synthesis of lamellarins according to the above-mentioned method [125]. By this procedure, they avoided the harsh conditions of the Bischler–Napieralski reaction for the subsequent synthesis of fused 5,6-dihydropyrrolo[2,1-α]isoquinolines like **393b** and **424**, especially when the phenolic hydroxy moieties contain acid-sensitive protecting groups. The substitution of compounds **421a**,**b** by aminonitrile **420** in the presence of KHDMS afforded derivatives **373c** and **423** after the removal of HCN from intermediates **422a**,**b** (Scheme 50). Michael addition of **373c** and **423** to β-nitro styrenes **374** and **392** in the presence of pyridines followed by ring closure afforded the fused pyrrole ring of compounds **393b** and **424**. Hydrogenolysis of the latter and lactonization in the presence of DBU resulted in the synthesis of lamellarin G trimethyl ether (**5**) and lamellarin U (**256**) in 33% and 35% yields, respectively, for the last 2 steps.

**Scheme 51:**
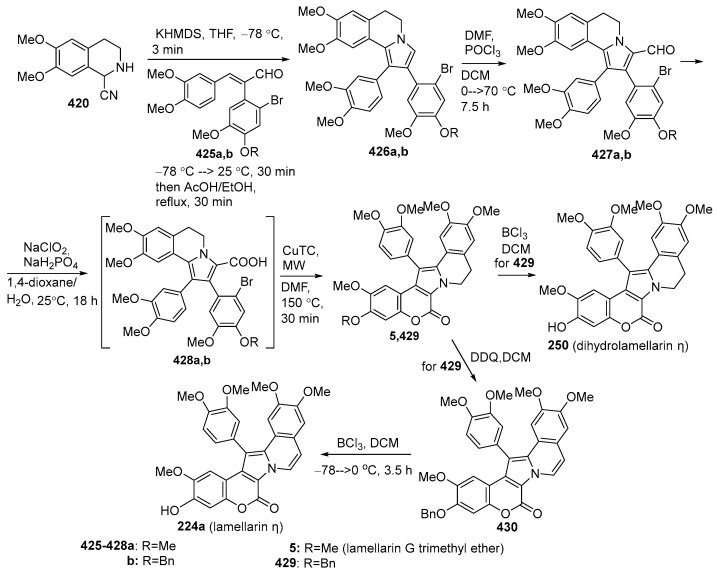
Synthesis of lamellarin G trimethyl ether (**5**), lamellarin η (**224a**), and dihydrolamellarin η (**250**).

The synthesis of lamellarin G trimethyl ether (**5**) was achieved by the same group in 69% total yield over 7 steps starting from 1,2,3,4-tetraydroisoquinoline-1-carbonitrile **420** [126]. The reaction of the latter in the presence of KHMDS with the aldehydes **425a**,**b** (prepared from 3,4-dialkoxyphenylacetonitrile) afforded fused 5,6-dihydropyrrolo[2,1-α]isoquinolines **426a**,**b**, which by Vilsmeier formylation furnished formyl-substituted pyrrole derivatives **427a**,**b** (Scheme 51). Lamellarin G trimethyl ether (**5**) and dihydrolamellarin η benzyl ether (**429**) were formed under oxidation of **427a**,**b** with sodium chlorite and treatment with copper(I)-thiophene-2-carboxylate (CuTC) upon microwave irradiation of intermediate acids **428a**,**b**. Selective deprotection of the benzyl group of the latter with BCl_3_ afforded dihydrolamellarin η (**250**) in 59% overall yield over 9 steps, while oxidation with DDQ furnished the benzyl ether of lamellarin η **430**. Similar selective deprotection with BCl_3_ in compound **430** resulted in the synthesis of lamellarin η (**424a**) in 54% overall yield over 10 steps.

**Scheme 52:**
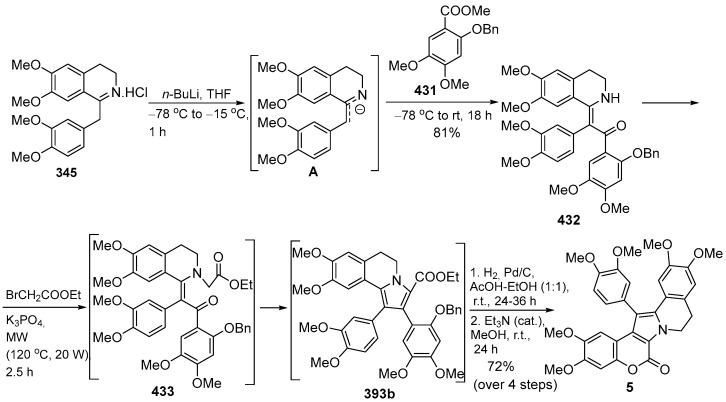
Synthesis of lamellarin G trimethyl ether (**5**).

Klintworth et al. presented a green synthesis of lamellarin G trimethyl ether (**5**) in 58–61% total yield starting from dihydropapaverine hydrochloride (**345**) and benzoate **431**, which have been prepared from starting materials derived from woody biomass sources like lignin and lignocellulose [127]. The key step of this synthesis was the preparation of enaminone **432** by the treatment of hydrochloride **345** with excess of *n*-BuLi to provide intermediate aza-enolate **A**, and subsequent reaction of the latter with benzoate **431** (Scheme 52). The reaction of enaminone **432** with ethyl bromoacetate, in analogy to the pioneering work of Iwao for the synthesis of lamellarin D [110], afforded lamellarin G trimethyl ether (**5**) via intermediate *N*-substituted enaminone **433** and pyrrole adduct **393b** followed by deprotection of benzyl group by hydrogenolysis and lactonization. In this process, no purification of the intermediates was necessary.

**Scheme 53:**
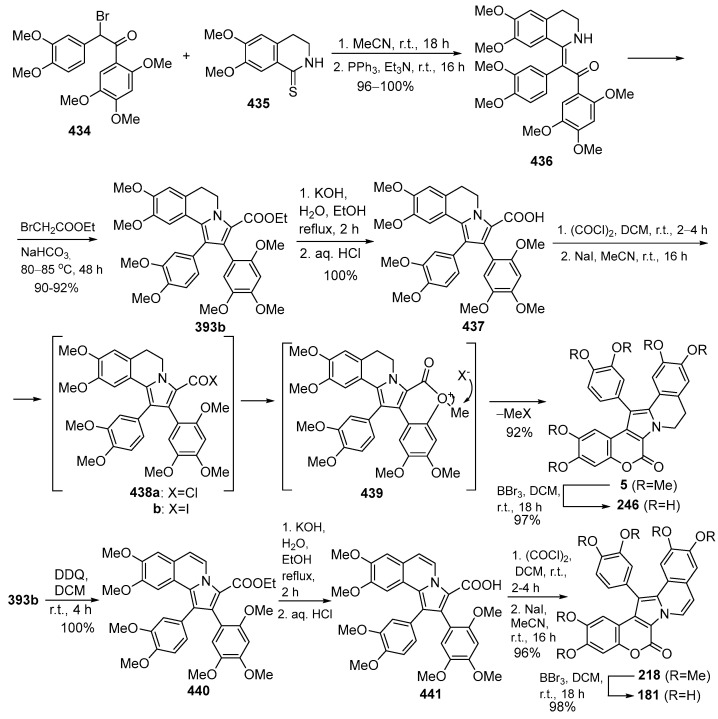
Synthesis of lamellarin G trimethyl ether (**5**), lamellarin A4 or trisdesmethyllamellarin G (**246**), lamellarin D trimethyl ether (**218**), and lamellarin H (**181**).

The same group, using another enaminone derivative prepared trimethyl ethers of lamellarin G (**5**) and D (**218**) in 82% (6 steps) and 86% overall yields (7 steps) [128]. Enaminone **436** was achieved from the Eschenmoser sulfide contraction of the reaction of bromoacetophenone derivative **434** with dihydroisoquinolinethione **435** (Scheme 53). The reaction of enaminone **436** with ethyl bromoacetate afforded pyrrole ester **393b**, which upon hydrolysis furnished pyrrole carboxylic acid **437**. Demethylative lactonization of intermediate **436**, upon formation of carboxylic acid chloride **438a** under treatment with oxalyl chloride and transformation to acid iodide **438b** by addition of NaI, and removal of NaCl, resulted in the synthesis of lamellarin G trimethyl ether (**5**). Oxidation of **393b** with DDQ to pyrrole carboxylic ester **440** followed by the above-presented reaction sequence afforded lamellarin D trimethyl ether (**218**). Treatment of trimethyl ethers **5** and **218** with BBr_3_ resulted in the synthesis of lamellarin A4 or trisdesmethyllamellarin G (**246**) and lamellarin H (**181**) in 97 and 98% yields, respectively.

Lamellarin A4 (**246**) was evaluated against P-glycoprotein-mediated multidrug resistance in human colon adenocarcinoma cell line SW620 Ad300 and the corresponding parental cell line SW620 and it was determined that it is no P-gp inhibitor as it is non-cytotoxic [63]. When **246** was assessed against neurodegenerative disease targets casein kinase 1 (CK1δ) and cyclin-dependent kinase 5 (CDK5), it was found to exhibit activity against CK1δ with IC_50_ = 3 μM.

**Scheme 54:**
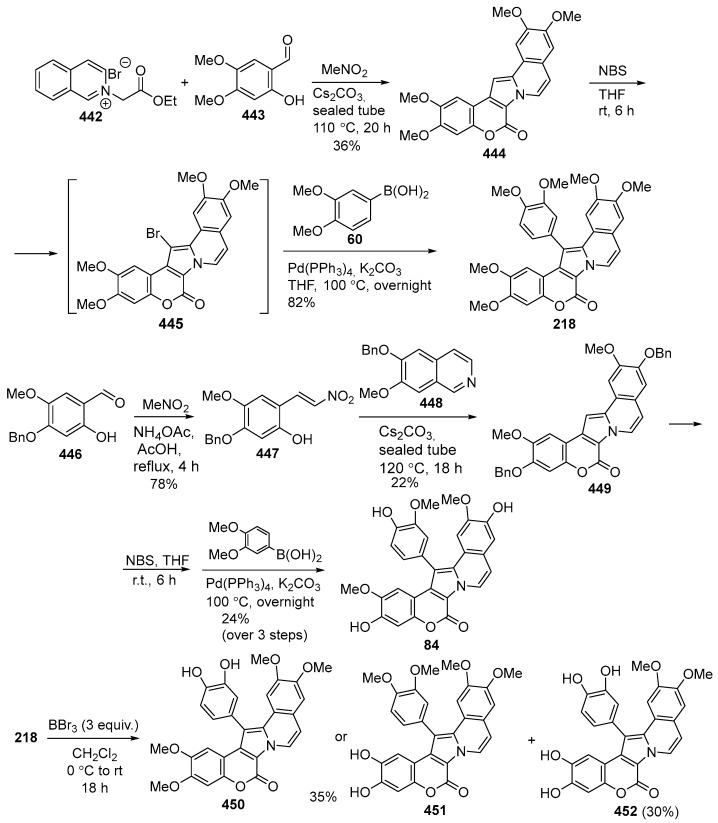
Synthesis of lamellarin D trimethyl ether (**218**), lamellarin D (**84**), and lamellarin analogues.

Manjappa et al. presented the synthesis of lamellarin D trimethyl ether (**218**), lamellarin D (**84**), and lamellarin analogues **450**–**452** using MCR constructing the lamellarin skeleton [129]. The multicomponent reaction of isoquinolinium salt **442** (prepared from the reaction of isoquinoline with ethyl bromoacetate), salicyl aldehyde **443**, and nitromethane in the presence of Cs_2_CO_3_ afforded, after pyrrole ring formation and lactonization, coumarin-fused pyrrolo[1,2-α]isoquinoline **444** (Scheme 54). Bromination of the latter with NBS to provide bromo-pyrrole derivative **445**, followed by Suzuki coupling reaction with boronic acid **60**, resulted in the synthesis of lamellarin D trimethyl ether (**218**) in 24% total yield over 3 steps. Lamellarin D (**84**) was synthesized starting from salicyl aldehyde **446** through the formation of α-nitrostyrene **447** with nitromethane, followed by a reaction with isoquinoline **448** to yield pyrrolocoumarin derivative **449**. Subsequent bromination with NBS, Suzuki coupling reaction, and debenzylation afforded lamellarin D (**84**) in 24% yield from **449**, following their previously used procedure for the synthesis of lamellarins H and D starting from coumarin derivatives [130]. Regioselective demethylation of **218** under treatment with excess of BBr_3_ furnished tetrahydroxy lamellarin derivative **452** in 30% yield, along with dihydroxy derivatives **450** or **451** in 35% yield.

**Scheme 55:**
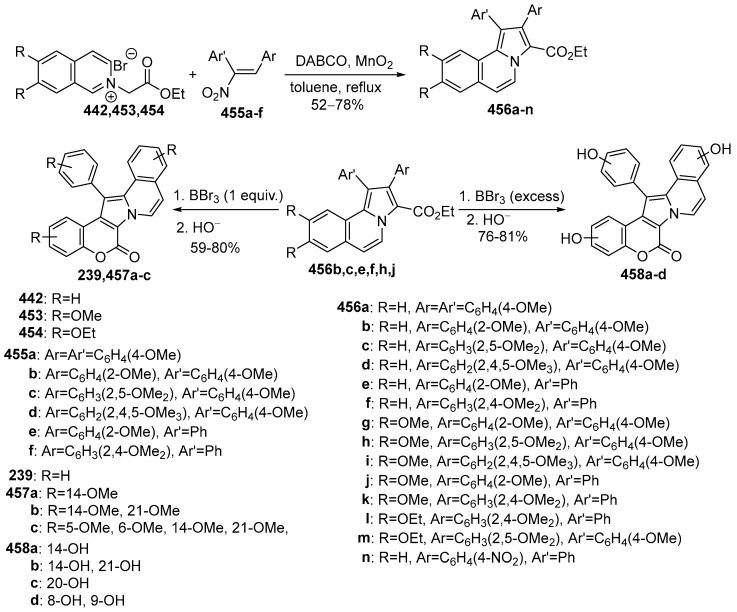
Synthesis of lamellarin analogues via 1,3-dipolar cycloaddition reaction of isoquinolinium ylides to nitrostilbenes.

Recently, Silyanova et al. reported the synthesis of 1,2-diarylpyrrolo[2,1-α]isoquinoline 3-carboxylates and their transformation to lamellarin analogues [131]. 1,3-Dipolar cycloaddition reaction of nitrostilbenes **455a**–**f** with isoquinolinium ylide, prepared in situ under treatment of isoquinolinium salt **442**, **453**, or **454** with DABCO, resulted in the preparation of pyrrolo[2,1-α]isoquinoline derivatives **456a**–**n** in 52–78% yields. This process involved the removal of HNO_2_ from the intermediate formed pyrrolidine and subsequent oxidation by MnO_2_ (Scheme 55). Deprotection of the methoxy group of compounds **456b**,**c**,**e**,**f**,**h**,**j** with 1 equivalent of BBr_3_ afforded unsubstituted lamellarin analogue **239** in 80% yield, and methoxy lamellarin analogues **457a**–**c** in 59-70% yields, while treatment with excess of BBr_3_ furnished hydroxy lamellarin analogues **458a**–**d** in 76–81% yields.

#### 2.1.3. Synthesis Starting from Coumarin Derivatives

**Scheme 56:**
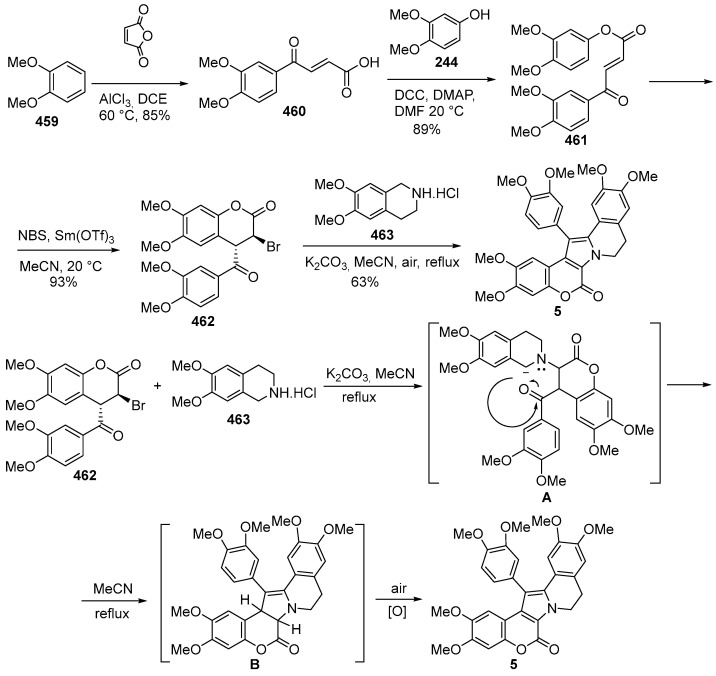
Synthesis of lamellarin G trimethyl ether (**5**) from 3-bromo-4-(3,4-dimethoxybenzoyl)-6,7-dimethoxychroman-2-one (**459**).

Yadav and coworkers synthesized lamellarin G trimethyl ether (**5**) preparing at first 3-bromo-4-(3,4-dimethoxybenzoyl)-6,7-dimethoxychroman-2-one (**462**) [132]. Friedel–Crafts reaction of veratrole (**459**) and maleic anhydride afforded α,β-unsaturated acid **460**, which upon esterification with phenol **244** furnished ester **461** (Scheme 56). Bromoarylation of ester **461** with NBS in the presence of Sm(OTf)_3_ resulted in the preparation of chroman-2-one **462**. Coupling compound **462** with 6,7-dimethoxy-1,2,3,4-tetrahydroisoquinoline hydrochloride (**463**) in the presence of K_2_CO_3_ in MeCN under reflux led to lamellarin G trimethyl ether (**5**) in 44% overall yield over 4 steps. For the mechanism of the reaction, *N*-alkylation of isoquinoline **463** with bromo derivative **462** furnished *N*-alkylated adduct **A**, which with intramolecular aldol-type condensation afforded dihydro-derivative **B**. Oxidation of the latter by air resulted in the formation of lamellarin **5**.

**Scheme 57:**
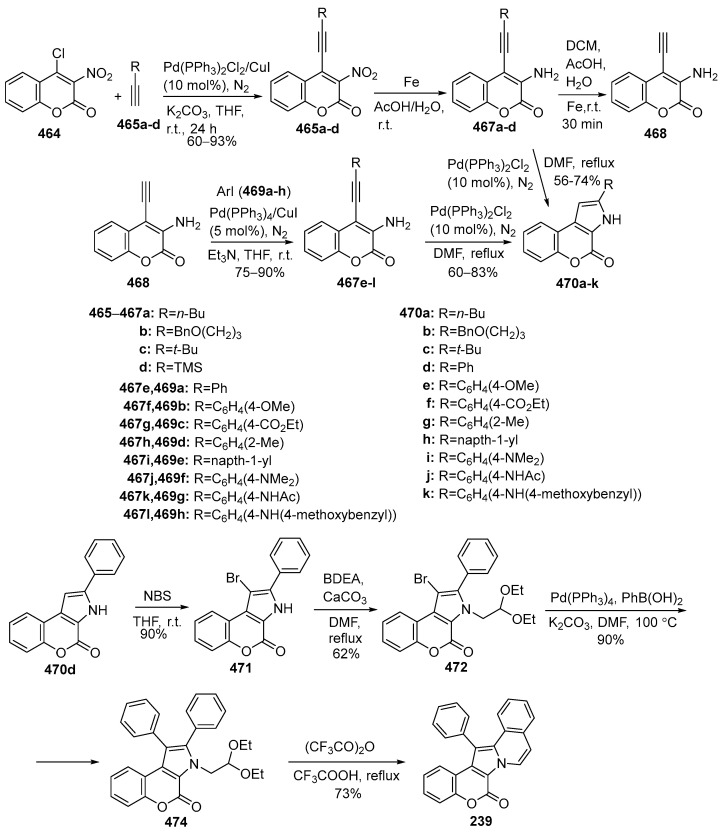
Synthesis of lamellarin scaffold **239** from 2-phenylchromeno[3,4-*b*]pyrrol-4(3*H*)-one (**470d**).

Chen and Hu reported the synthesis of lamellarin scaffold **239** from 2-phenylchromeno[3,4-*b*]pyrrol-4(3*H*)-one (**470d**) [133]. 2-Alkylchromeno[3,4-*b*]pyrrol-4(3*H*)-ones **470a**–**c** were synthesized starting from 4-chloro-3-nitrocoumarin (**464**) by Sonogashira cross-coupling reaction with terminal alkynes **465a**–**d** followed by reduction and C-N coupling reaction catalyzed by Pd(PPh_3_)Cl_2_ (Scheme 57). 2-Arylchromeno[3,4-*b*]pyrrol-4(3*H*)-ones **470d**–**k** were achieved from 3-amino-4-ethynylcoumarin (**468**) through Sonogashira coupling reaction with aryl iodides **469a**–**h** and subsequent Pd-catalyzed C-N coupling reaction of intermediate derivatives **470e**–**l**. Bromination of compound **470d** with NBS to provide bromo-pyrrole **471** followed by substitution of bromo-acetaldehyde diethyl acetal (BDEA) afforded *N*-alkylated derivative **472**. Suzuki cross-coupling of the latter with phenylboronic acid furnished diphenyl adduct **473**. Treatment of **473** with (CF_3_CO)_2_O/CF_3_CO_2_H resulted in the synthesis of pentacyclic lamellarin moiety **239**.

**Scheme 58:**
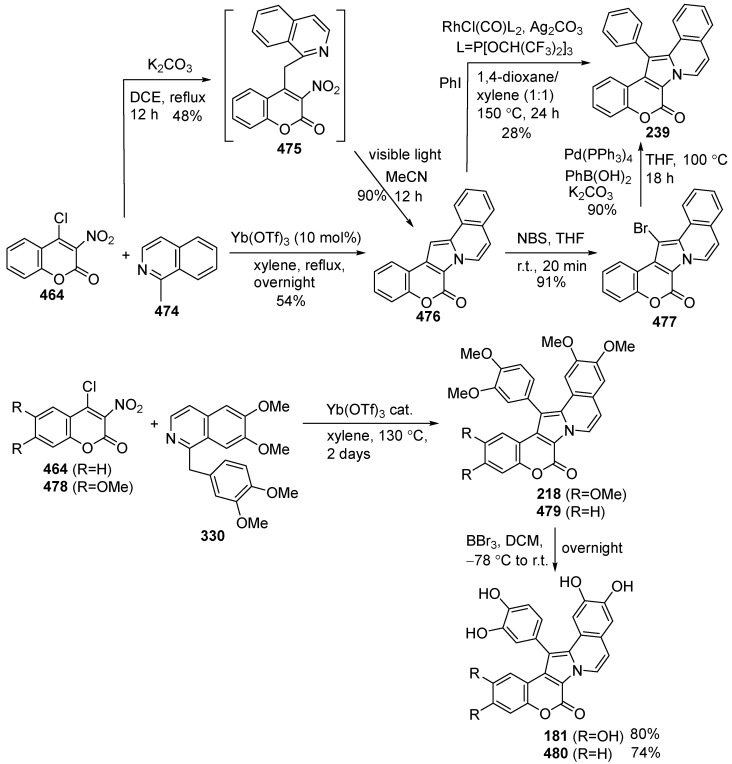
Synthesis of lamellarin D trimethyl ether (**218**), lamellarin H (**181**), and the lamellarin analogues **479** and **480**.

Manjappa et al. demonstrated the construction of a coumarin–pyrrole–isoquinoline-fused pentacycle like **476** using the visible-light-promoted cyclization of 4-(isoquinolin-1-ylmethyl)-3-nitrocoumarin (**475**) or the Yb(OTf)_3_-catalyzed coupling of 4-chloro-3-nitrocoumarin (**464**) with 1-methylisoquinoline (**474**) [134]. Bromination of compound **476** with NBS followed by Suzuki coupling with phenylboronic acid afforded lamellarin core **239**, which was also achieved by Rh-catalyzed coupling with phenyl iodide (Scheme 58). Lamellarin D trimethyl ether (**218**) was synthesized by application of this method from the reaction of 4-chloro-6,7-dimethoxy-3-nitrocoumarin (**478**) with 1-benzyl isoquinoline **330** in the presence of Yb(OTf)_3_. Deprotection of the latter with BBr_3_ resulted in the synthesis of lamellarin H (**181**). Lamellarin analogues **479** and **480** were also synthesized by this method.

**Scheme 59:**
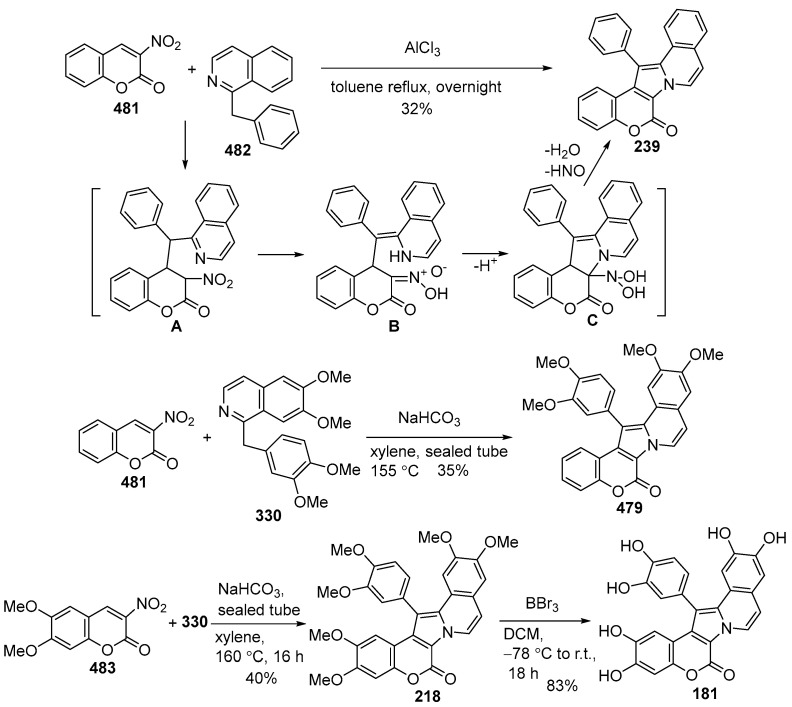
Synthesis of lamellarin D trimethyl ether (**218**) and lamellarin H (**181**).

**Scheme 60:**
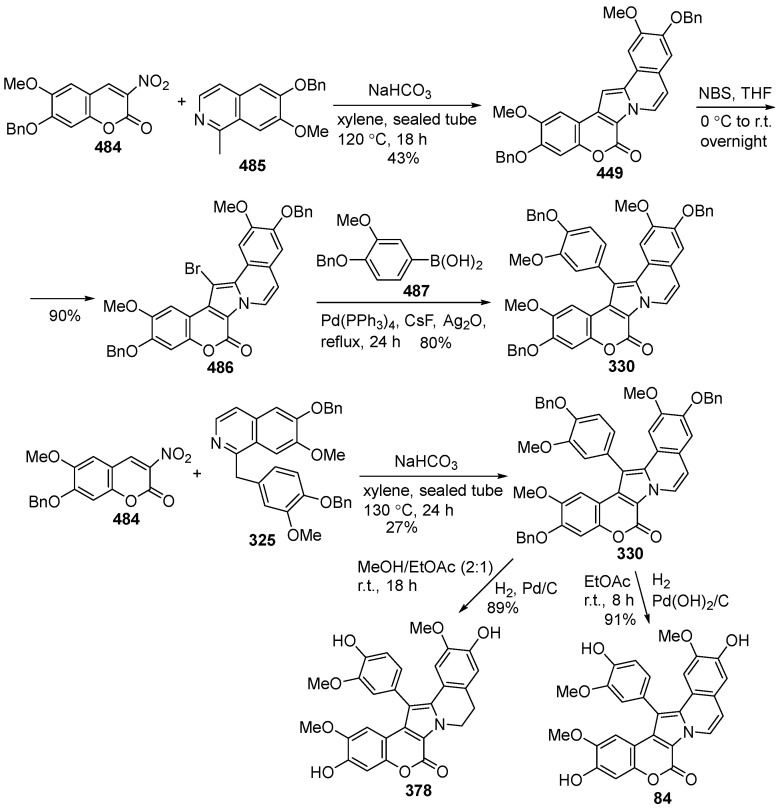
Synthesis of lamellarin D (**84**) and 501 (**378**) and tribenzyl ether of lamellarin D (**98**).

The same group demonstrated the synthesis of lamellarin core **239** from the reaction of 3-nitrocoumarin (**481**) with 1-benzylisoquinoline (**482**) [130], replacing 1-benzyl-3,4-dihydroisoquinoline used earlier by Ploypradith et al. [118] producing lamellarins in only 5–6% yield. Derivative **239** was achieved in 32% yield by refluxing a mixture of **481** and **482** in toluene in the presence of AlCl_3_ via the intermediates **A**, **B**, and **C** following the removal of water and nitroxyl (Scheme 59). They also synthesized the lamellarin analogue **479** by the NaHCO_3_-promoted Grob-type coupling of 3-nitrocoumarin (**481**) and papaverine (**330**) in a sealed tube. The same method with 6,7-dimethoxy-3-nitrocoumarin (**483**) and compound **330** as starting material afforded lamellarin D trimethyl ether (**218**) in 40% yield, which after deprotection of methoxy group with BBr_3_ furnished lamellarin H (**181**) in 31% overall yield in 3 steps. Tribenzyl ether of lamellarin D (**330**) was achieved in 31% yield, over 3 steps, from the similar reaction of 7-benzyloxy-6-methoxy-3-nitrocoumarin (**484**) with 6-benzyloxy-7-methoxy-1-methylisoquinoline (**485**) to provide derivative **449**, bromination of the latter with NBS and Suzuki cross-coupling of derivative **486** formed with boronic acid **487** (Scheme 60). The Grob-type coupling of compound **484** with 1-benzylquinoline **325** resulted in the synthesis of tribenzyl ether **330** in 27% yield. Selective hydrogenation of the latter with Pd(OH)_2_/C afforded lamellarin D (**84**) in 12% or 14% in 6 or 8 steps overall yields, respectively, while hydrogenation with Pd/C furnished lamellarin 501 (**378**).

**Scheme 61:**
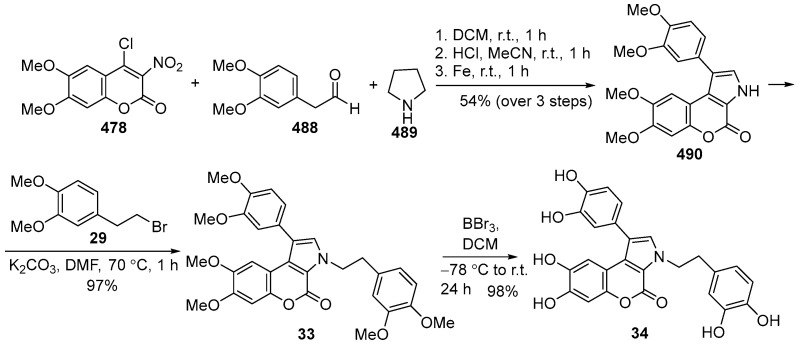
Synthesis of ningalin B (**34**).

Wu et al. reported the synthesis of ningalin B (**34**) starting from a three-component reaction of 4-chloro-6,7-dimethoxy-3-nitrocoumarin (**478**), (3,4-dimethoxyphenyl)acetaldehyde (**488**), and pyrrolidine (**489**) [135]. By mixing the above-mentioned reagents in DCM for 1 h and after the addition of HCl and iron powder, pyrrolocoumarin **490** was achieved (Scheme 61). Alkylation of the latter with 4-(2-bromoethyl)-1,2-dimethoxybenzene (**29**) afforded hexamethyl ether of ningalin B (**33**), which upon exhaustive demethylation with BBr_3_ furnished the ningalin B (**34**) in 41.5% overall yield over five steps.

Ningalin B hexamethyl ether (**33**) was found to have immunosuppressive activity in tumor bearing mice [136]. Derivative **33** also exhibited an anti-inflammatory effect by intraperitonial injections in mice.

### 2.2. Synthesis of Isolamellarins and Analogues

**Scheme 62:**
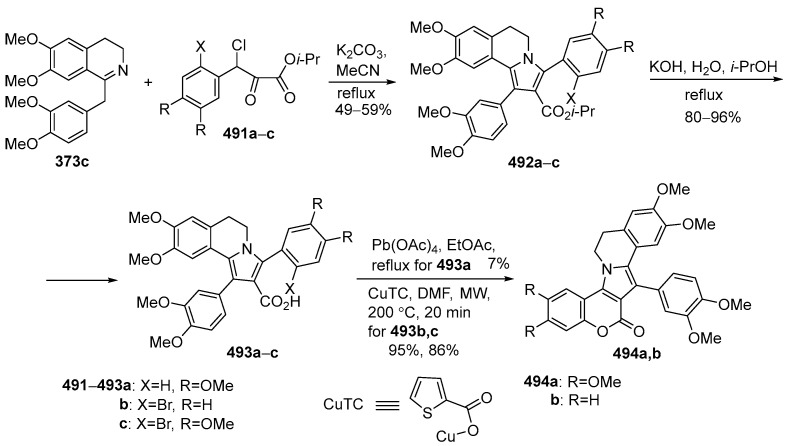
Synthesis of isolamellarins A **494a**,**b**.

Thassana et al. presented the synthesis of isolamellarins A **494a**,**b** from dihydroisoquinoline and aryl puruvates under basic conditions and Cu-mediated C_aryl_-O_carboxylic acid_ lactonization under microwave irradiation [137]. The reaction of dihydroisoquinoline **373c** with aryl puruvates **491a**–**c** afforded the fused pyrrolo[2,1-a]isoquinoline esters **492a**–**c** (Scheme 62). Basic hydrolysis of the latter followed by oxidation with Pb(OAc)_4_ for **493a** or CuTC (copper (I) thiophene-2-carboxylate) treatment under MW for the intermediates **493b**,**c** resulted in the synthesis of isolamellarins **494a**,**b** in 3% (from **493a**) or 48% (from **493c**) and 40% (from **493b**) overall yields, respectively.

**Scheme 63:**
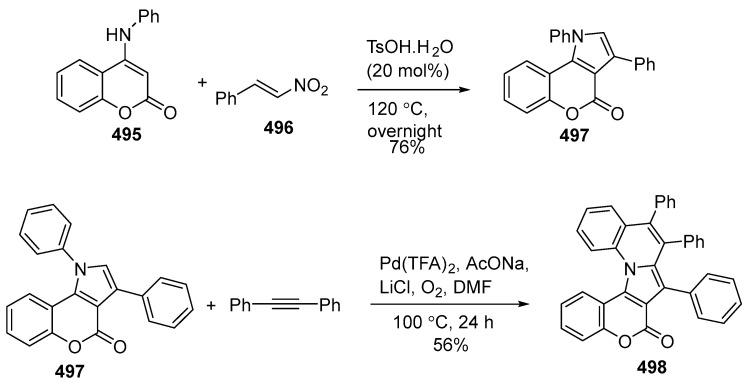
Synthesis of pentacyclic compound **498**.

Padilha et al. used *p*-toluenesulfonic acid for the synthesis of 1,3-diphenylchromeno[4,3-*b*]pyrrol-4(1*H*)-ones **497** from 4-*N*-phenylaminocoumarins **495** and β-nitrostyrene (**496**) under solvent-free conditions (Scheme 63). Compound **497** reacted with diphenyl acetylene under oxidative cyclocondensation reaction to provide the pentacyclic derivative **498**, analogue of isolamellarin skeleton in 43% yield over 2 steps [138].

**Scheme 64:**
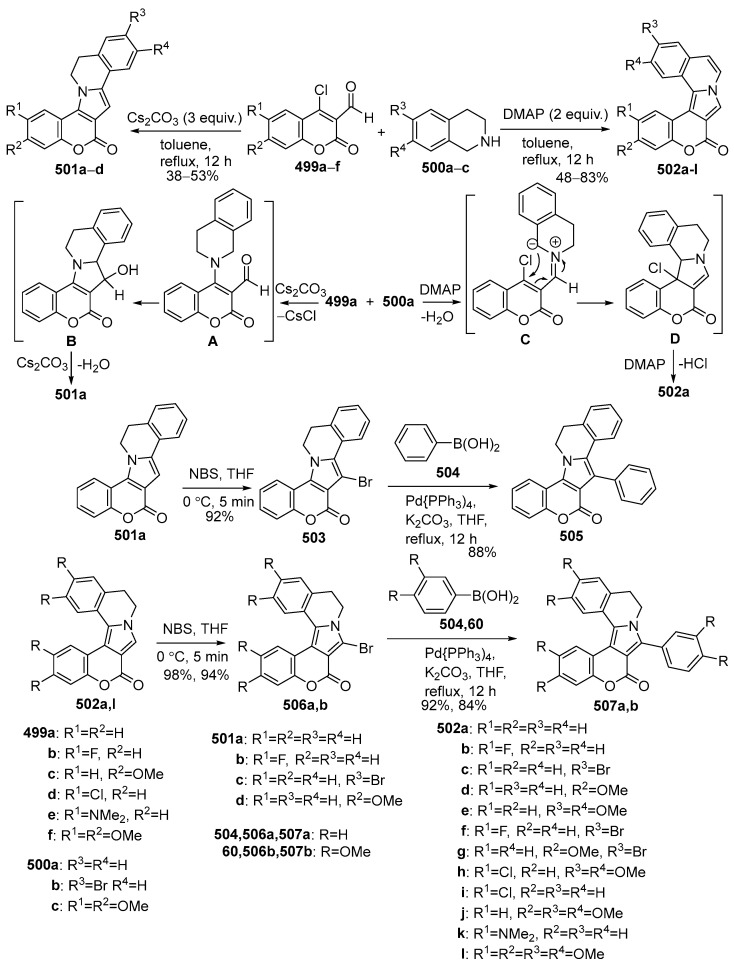
Synthesis of isolamellarin A **505** and isolamellarins B **507a**,**b**.

Vyasamudri and Yang demonstrated the synthesis of isolamellarin A **505** and B **507a**,**b** starting from the reactions of 4-chloro-3-formylcoumarins **499a**–**f** with 1,2,3,4-tetrahydroisoquinolines **500a**–**c** [139]. The reactions of coumarins **499a**–**f** with tetrahydroisoquinolines **500a**–**c** in the presence of Cs_2_CO_3_ as a base selectively afforded the pentacycle derivatives **501a**–**d** in 38–53% yields, while the presence of DMAP as the base resulted in the synthesis of pentacycles **502a**–**l** in 48–83% yields (Scheme 64). Bromination of compounds **501a** and **502a**,**l** with NBS furnished the bromo-pyrrole derivatives **503** and **506a**,**b**, respectively, which by Suzuki cross-coupling reaction with boronic acids **504** or **60** afforded isolamellarin A **505** and isolamellarins B **507a**,**b**, respectively. Compound **507b** is the isolamellarin G trimethyl ether.

**Scheme 65:**
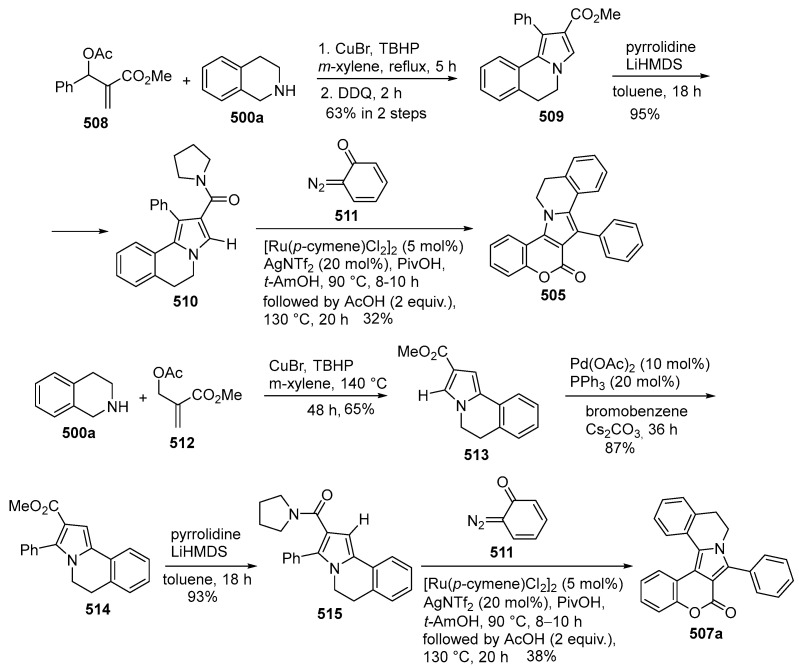
Synthesis of isolamellarin A **505** and isolamellarin B **507a** under Ru(II)-catalysis.

Sarkar and Samanda reported the synthesis of isolamellarins A **505** and B **507a** under *tert*-amide-assisted Ru(II)-catalyzed insertion of quinoid carbene in substituted indole-carboxylic acid amides during the synthesis of azacoumestans and related heterocycles [140]. The reaction of acrylate **508** with 1,2,3,4-tetrahydroisoquinoline (**500a**) in the presence of CuBr/TBHP followed by oxidation with DDQ afforded pyrrole[2,1-*a*]isoquinoline derivative **509**, which by treatment with pyrrolidine and LiHMDS furnished the amide **510** (Scheme 65). Ru(II)-catalyzed reaction of the latter with *o*-quinonediazide **511** resulted in the formation of isolamellarin A **505** in 19% overall yield. The reaction of **500a** with allyl acetate **512** in the presence of CuBr/TBHP furnished pyrrole[2,1-*a*]isoquinoline derivative **513**. Site-selective C2-arylation of the latter under Pd(OAc)_2_ catalysis afforded 2-phenyl pyrrole derivative **514**, which by treatment with LiHMDS and pyrrolidine provided the amide **515**. Similarly, reaction with *o*-quinonediazide **511** under Ru(II)-catalysis resulted in the synthesis of isolamellarin B **507a** in 20% overall yield.

### 2.3. Synthesis of Azacoumestans, Isoazacoumestans, and Analogues

**Scheme 66:**
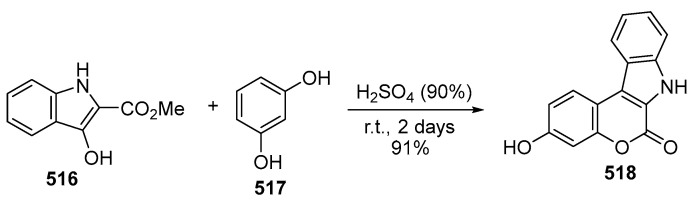
Synthesis of isoazacoumestan **518**.

In 1948, King et al. synthesized for the first time 3-hydroxychromeno[3,4-*b*]indol-6(7*H*)-one (**515**), an isoazacoumestan [141]. The von Pechmann reaction of methyl 3-hydroxyindole-2-carboxylate (**516**) with resorcinol (**517**) afforded isoazacoumestan **518** in 91% yield (Scheme 66).

**Scheme 67:**
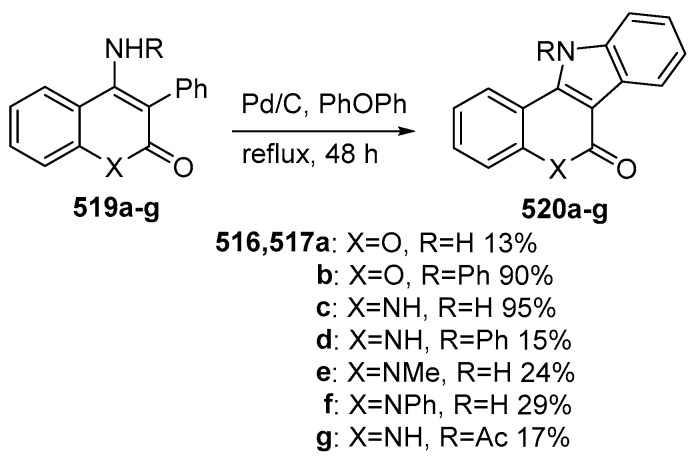
Synthesis of azacoumestans **520a**,**b**.

In 1984, Stadlbauer and Kappe reported the first synthesis of azacoumestans [142]. The reflux of 4-amino-3-phenylcoumarin **519a**,**b** in diphenyl ether in the presence of Pd/C resulted in the synthesis of azacoumestans **520a**,**b** in 13% and 90% yields, respectively (Scheme 67). This reaction proceeded via cyclodehydrogenation. 11*H*-Indolo[3,2-*c*]quinolin-6(5*H*)-ones **520c**–**g** have been also been synthesized by this method.

Azacoumestans are known to have antiosteoporotic activity [143].

**Scheme 68:**
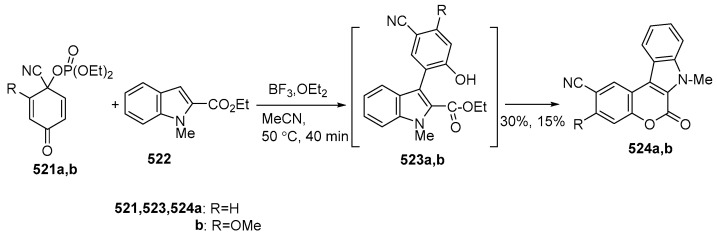
Synthesis of isoazacoumestans **524a**,**b**.

The synthesis of isoazacoumestans **524a**,**b** was demonstrated by Kurihara and coworkers [144]. The reaction of 1,4-benzoquinone cyanohydrin phosphates **521a**,**b** with ethyl *N*-methyl indol-2-carboxylate (**522**) in the presence of boron trifluoride etherate resulted in the synthesis of isoazacoumestans **524a**,**b** via the intermediate 3-arylindole-2-carboxylates **523a**,**b** in 30% and 15% yields, respectively (Scheme 68).

**Scheme 69:**
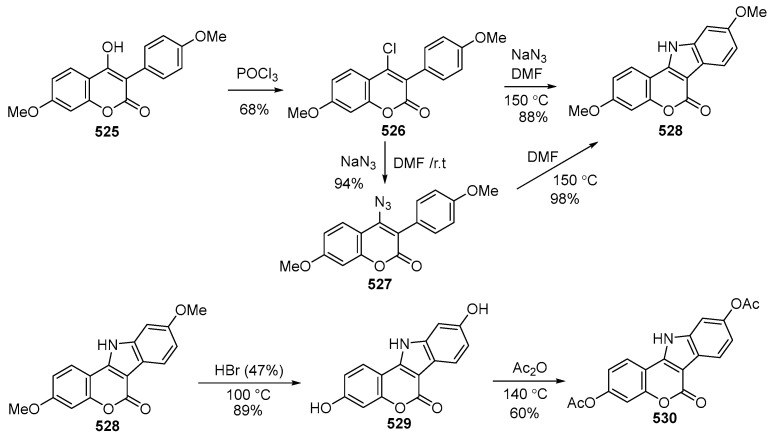
Synthesis of azacoumestrol (**529**).

Stadlbauer and Kappe presented the synthesis of azacoumestrol (**529**) and azacoumestrol dimethyl ether **528** and diacetate **530** [68,143]. 4-Hydroxycoumarin derivative **525** under chlorination with POCl_3_ afforded the 4-chlorocoumarin derivative **526** (Scheme 69). Treatment of the latter with sodium azide in DMF at 150 °C afforded azacoumestrol dimethyl ether (**528**) in 88% yield. When the same reaction was performed at room temperature, the 4-coumarinyl azide **527** was synthesized, which upon heating at 150 °C furnished compound **528** in 92% total yield. Deprotection of the methyl group with HBr resulted in the synthesis of azacoumestrol (**529**) in 89% yield. Reaction of **529** with acetic anhydride afforded the diacetate **530**.

Azacoumestrol (**529**) was identified as a non-cytotoxic compound that inhibits vascular endothelial growth factor receptor-2 (VEGFR2) kinase effectively, blocking the progression of angiogenesis in the low micromolar range (10μM) [43].

**Scheme 70:**
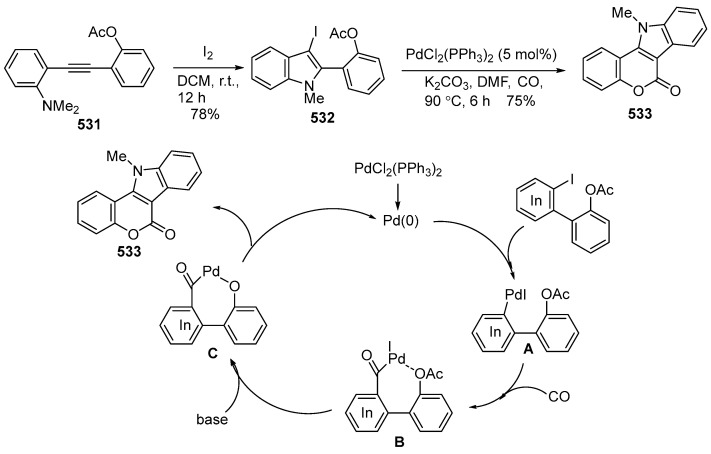
Pd-catalyzed synthesis of azacoumestan **533**.

Larock and coworkers synthesized azacoumestan **533** from 2-aryl-3-iodoindole derivative **532** upon Pd-catalyzed carbonylation/lactonization reactions [145]. 3-Iodoindole compound **532** was achieved by iodocyclization from diaryl alkyne **531**. In the proposed mechanism for this carbonylation, oxidative addition of Pd(0) to indolyl iodide afforded the Pd(II)-intermediate **A**. Insertion of CO to this generated the acyl-palladium intermediate **B** (Scheme 70). Deacylation in the presence of K_2_CO_3_ furnished complex **C**, which by reductive elimination resulted in the formation of azacoumestan **533**, regenerating the Pd-catalyst.

**Scheme 71:**
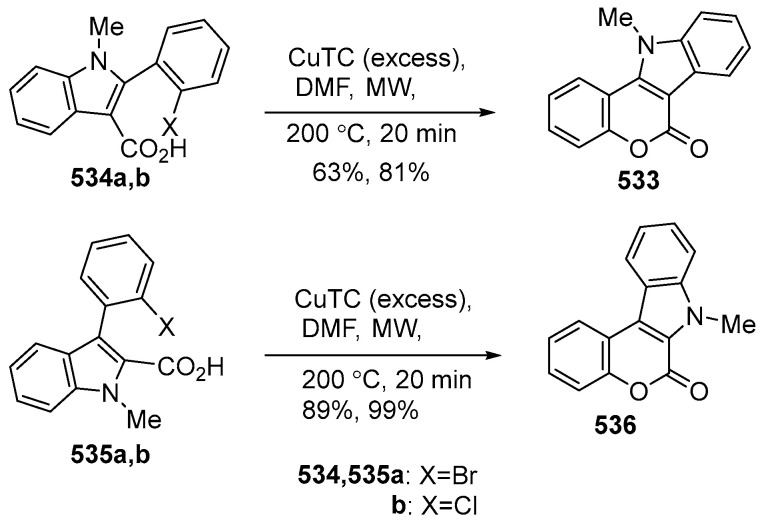
Synthesis of azacoumestan **533** and isoazacoumestan **536**.

Thasana and coworkers demonstrated the synthesis of azacoumestan **533** and isoazacoumestan **536** along with the referred synthesis of isolamellarins A **494a**–**c** in Scheme 62 [137]. The treatment of (2-haloaryl) indolecarboxylic acids **534a**,**b** or **535a**,**b** with excess of copper thiophene-2-carboxylate under microwave irradiation afforded azacoumestan **533** or isoazacoumestan **536** through C-O carboxylic coupling reaction in 63–99% yields (Scheme 71).

**Scheme 72:**
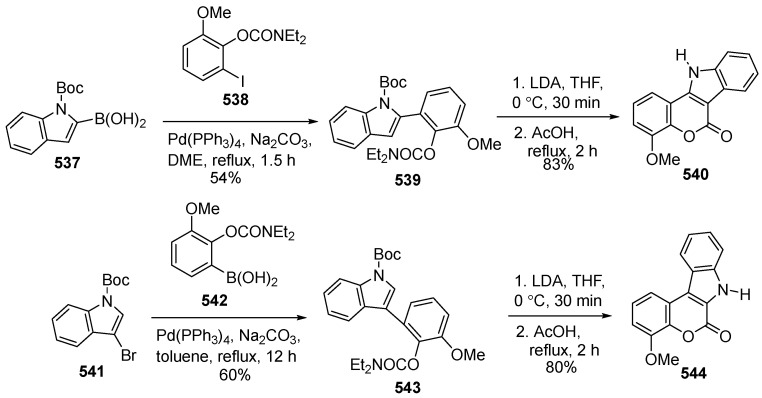
Synthesis of azacoumestan **540** and isoazacoumestan **544**.

Snieckus and coworkers presented the synthesis of azacoumestan **540** and isoazacoumestan **544** by the LDA-induced C-O coupling of *o*-indoloaryl *O*-carbamates **539** and **543** in 83 and 60% yields, respectively [146]. The synthesis of derivatives **539** and **543 was** achieved by the Suzuki cross-coupling reaction of aryl iodide **538** with 2-indoleboronic acid **537** and arylboronic acid **542** with 3-bromoindole **541** in 54 and 60% yields, respectively (Scheme 72).

**Scheme 73:**
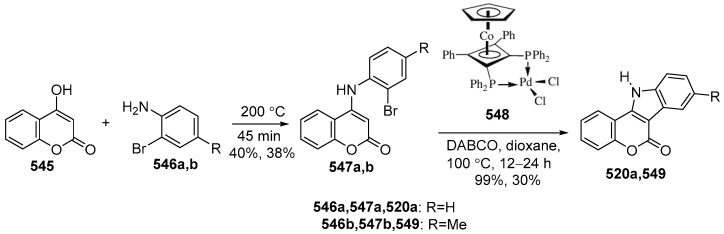
Pd-catalyzed synthesis of azacoumestanes **520a** and **549**.

Chang et al. have prepared the cobalt-sandwich diphosphine chelate palladium complex **548**, which was used as catalyst for the synthesis of azacoumestans **520a** and **549** in 73–79% isolated yields by the intramolecular Heck coupling reaction of 4-anilinocoumarins **547a**,**b** [147]. The latter was achieved by the heating of 4-hydroxycoumarin (**545**) and anilines **546a**,**b** (Scheme 73).

**Scheme 74:**
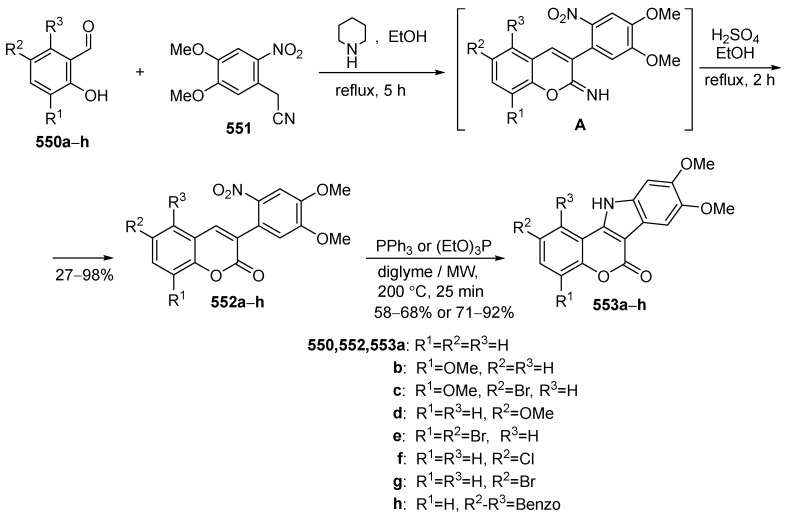
Synthesis of azacoumestans **553a**–**h** by the Cadogan reaction.

Irgashev et al. demonstrated the synthesis of indol[3,2-*c*]coumarins (azacoumestans) **553a**–**h** by the Cadogan reaction from 3-(*o*-nitroaryl)coumarins **552a**–**h** [148]. Perkin condensation of salicylaldehydes **550a**–**h** with benzyl cyanide **551** afforded derivatives **552a**–**h**. Treatment of the latter with PPh_3_ or P(OEt)_3_ under microwave irradiation at 200 °C resulted in the synthesis of azacoumestans **553a**–**h** in 16–85% total yields over 2 steps (Scheme 74).

**Scheme 75:**
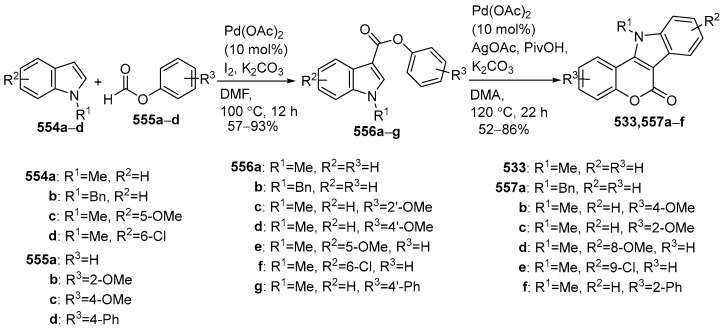
Pd-catalyzed synthesis of azacoumestans **533** and **557a**–**f**.

Wu et al. synthesized the azacoumestans **533** and **557a**–**f** in 52–86% yields by the Pd-catalyzed intramolecular oxidative C-H/C-H coupling of 3-indolcarboxylic acid aryl esters **556a**–**g** [149]. The latter was achieved in 65–93% yields by the Pd-catalyzed C-H carbonylation of indoles **554a**–**d** by aryl formates **555a**–**d** (Scheme 75).

**Scheme 76:**
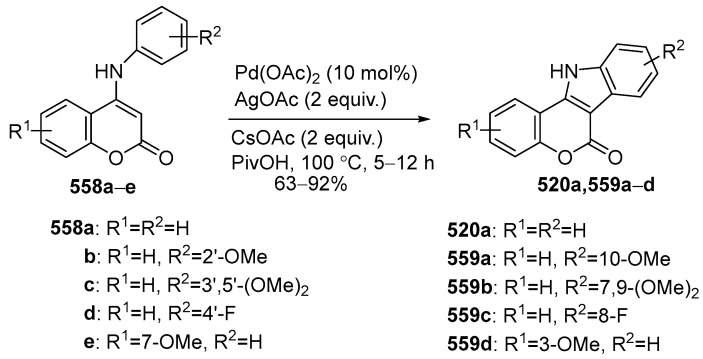
Pd-catalyzed synthesis of azacoumestans **520a** and **559a**–**d**.

Cheng et al. presented the Pd-catalyzed synthesis of azacoumestans and coumestans [150]. Pd-catalyzed intramolecular cross-dehydrogenative coupling of 4-arylaminocoumarins **558a**–**e** in the presence of excess of AgOAc as oxidative agent and CsOAc as base afforded the azacoumestans **520a** and **559a**–**d** in 63–92% yields (Scheme 76).

**Scheme 77:**
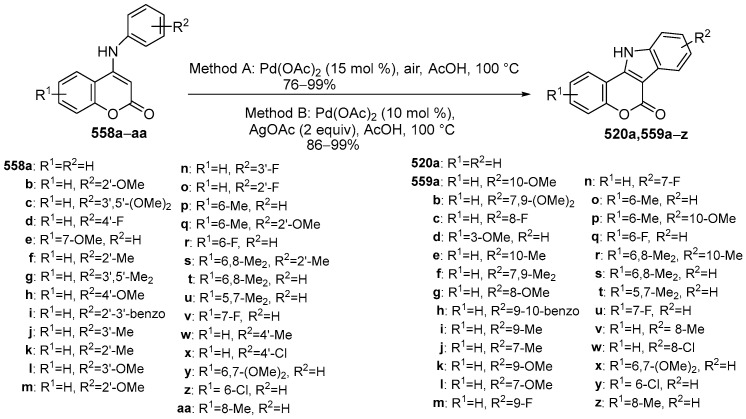
Pd-catalyzed synthesis of azacoumestans **520a** and **559a**–**z** (without base).

The same group also demonstrated the Pd-catalyzed intramolecular cross-dehydrogenative coupling reactions of 4-anilinocoumarins **558a**–**aa** for the synthesis of azacoumestans under base-free conditions in better yields [151]. Method A with air as oxidant was utilized for the synthesis of compounds **520a** and **550a**–**n** in 76–99% yields, while Method B with AgOAc as oxidant was used for the synthesis of compounds **559o**–**z** in 86–99% yields (Scheme 77).

**Scheme 78:**
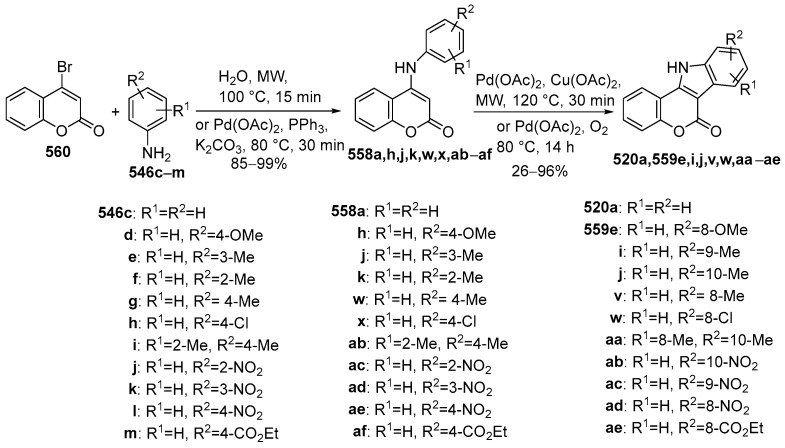
Pd-catalyzed synthesis of azacoumestans **520a** and **559e**,**I**,**j**,**v**,**w**,**aa**–**ae** under MW.

In 2017, Balalas et al. presented the Pd-catalyzed synthesis of azacoumestans under microwave irradiation [152]. The Pd-catalyzed oxidative coupling of (4-arylamino)coumarins **558** in the presence of Cu(OAc)_2_ under microwave irradiation or in the presence of O_2_ under reflux afforded the azacoumestans **520a** and **559** in 50–96% yields (Scheme 78). (4-Arylamino) coumarins have been prepared under Pd-catalyzed C-N coupling from 4-bromocoumarin (**560**) and aniline **546** and heating in toluene, resulting in 85–99% yields. The arylaminocoumarins not having electron-withdrawing group were also achieved quantitatively by substitution of compound **560** with the corresponding anilines under microwaves.

In the preliminary biological tests, most of the azacoumestans exhibited high (100%) anti-lipid peroxidation at 0.1 mM. Compounds **520a** and **559e** presented IC_50_ values of 26.5 and 26 μM, respectively, as inhibitors of soybeal lipoxygenase, an enzyme implicated in inflammation.

**Scheme 79:**
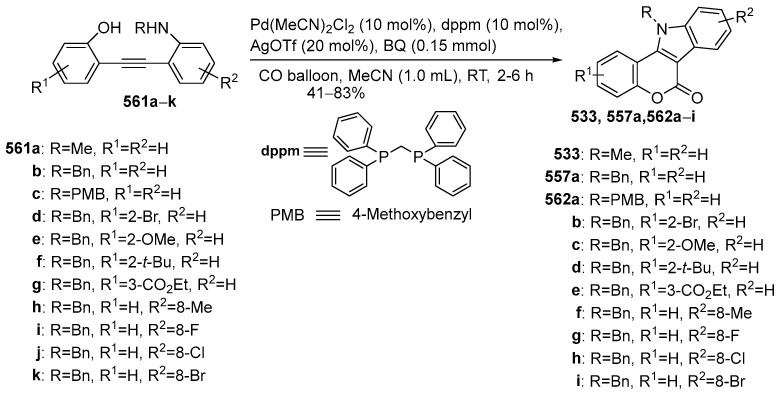
Pd-catalyzed synthesis of azacoumestans from alkynes under carbonylation.

Ding et al. reported the synthesis of indolo[3,2-c]coumarins by the Pd(II)-catalyzed carbonylated cyclization of o-hydroxy-o’-aminosubstituted alkynes **561a**–**k** regioselectively in the presence of bulky and electron-rich ligands (Scheme 79). The reaction of alkynes **561a**–**k**, etc., in the presence of Pd(MeCN)_2_Cl_2_ as catalyst, bis(diphenylphosphino)methane (DPPM) as ligand, 1,10-phenanthroline-5,6-dione (BQ) as oxidant under cyclization, carbonylation, and lactonization afforded azacoumestans **533**, **557a**, **562a**–**l**, and some others with more complicated structures [153].

**Scheme 80:**
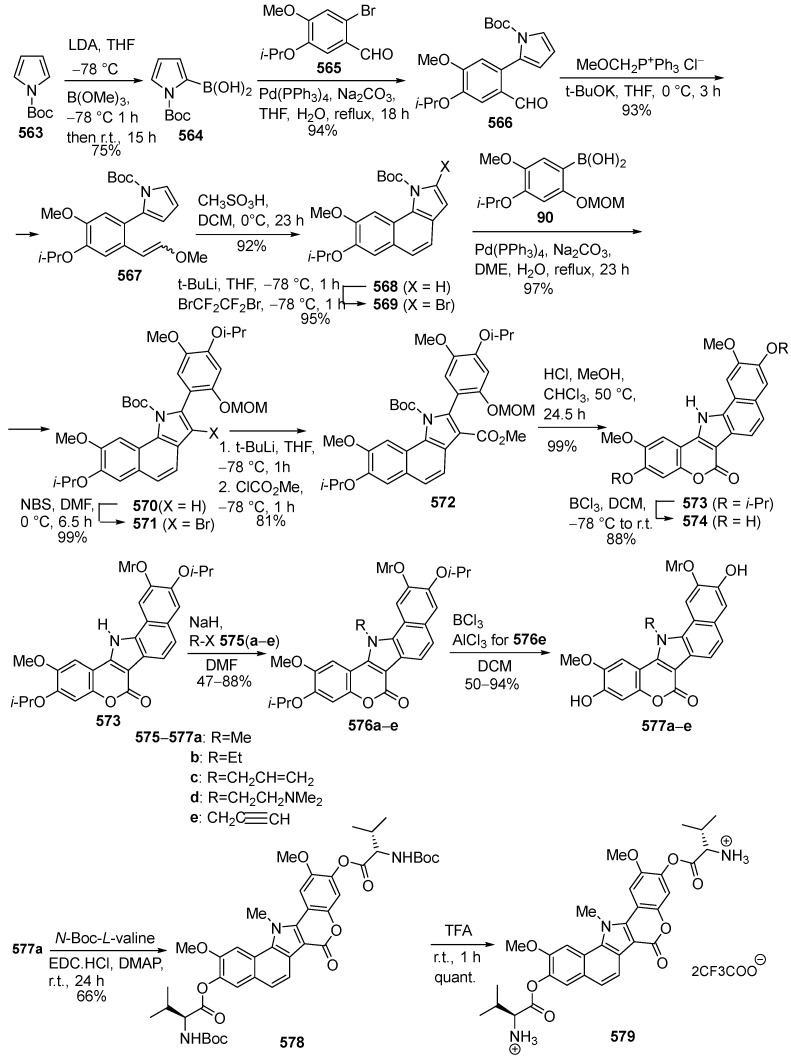
Synthesis of azacoumestan derivatives **573**, **574**, and **576**–**579**.

Fukuda et al. synthesized the azacoumestan derivatives **573**, **574**, **576**–**577a**–**e**, **578**, and **579** and tested them for their potent topoisomerase I inhibitory activity in DNA relaxation assays [93]. N-Boc pyrrole (**563**) reacted with B(OMe)_3_ in the presence of LDA to provide pyrrole-2-boronic acid **564**, which by a Suzuki cross-coupling reaction with o-bromobenzaldehyde derivative **565** afforded aldehyde **566** (Scheme 80). Wittig reaction of the latter with methoxymethylene triphenylphosphorane furnished enol ether **567**. Acid-catalyzed cyclization resulted in the formation of benzo[g[indole derivative **568**. Lithiation of the latter with t-BuLi followed by reaction with 1,2-dibromo-1,1,2,2-tetrafluoroethane afforded the 2-bromoindole derivative **569**. Suzuki cross-coupling reaction with boronic acid **90** provided the 2-aryl indole adduct **570**, which by electrophilic bromination with NBS furnished the 3-bromo-indole compound **571**. Treatment with t-BuLi for a bromine-lithium exchange followed by a subsequent substitution of methyl chloroformate furnished the ester **572**. Simultaneous deprotection of Boc and MOM group by hydrochloric acid and lactonization resulted in the formation of benzo[g][1]benzopyrano[4,3-b]indol-6(13H)-one skeleton of **573**. Selective deporotection of isopropyl group by BCl_3_ provided derivative **574** in 39% overall yield in 10 steps from compound **563**. Alkylation of compound **573** with alkyl halides **575a**–**e** afforded N-alkyl derivatives **576a**–**e**, which under deprotection with BCl_3_ furnished derivatives **577a**–**e**. The water-soluble valine ester **579** was prepared from N-methyl derivative **577a** by treatment with N-Boc-L-valine in the presence of EDC and DMAP to provide O-valinate ester **578**, followed by cleavage of N-Boc groups with TFA.

The topoisomerase I inhibitory activity of compounds **574**, **577a**, **577d**, and **579 was** found to be higher than that of reference compounds camptothecin (topoisomerase I inhibitor) and lamellarin D (**84**). The water-soluble compound **579** also exhibited antitumor activity against colon 26 (murine colon carcinima cell lines) comparable to the approved anticancer agent irinotecan.

**Scheme 81:**
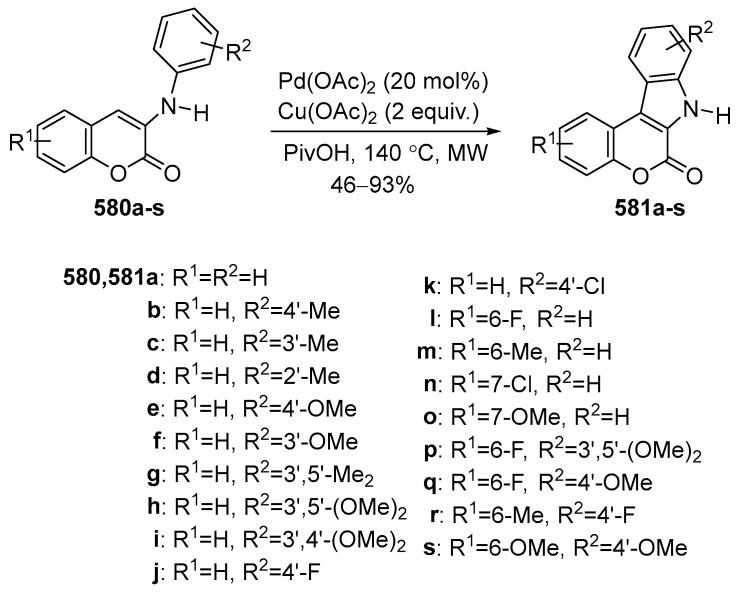
Synthesis of isoazacoumestns **581a**–**s**.

Gu et al. reported the Pd-catalyzed synthesis of indolo[2,3-*c*]coumarins **581a**–**s** from 3-arylaminocoumarins **580a**–**s** under microwave irradiation [154]. The Pd-catalyzed cross-dehydrogenative coupling (CDC) of 3-anilinocoumarins **580a**–**s** under microwave irradiation and base-free conditions resulted in the synthesis of isoazacoumestans **581a**–**s** in 46–88% yields (Scheme 81).

**Scheme 82:**
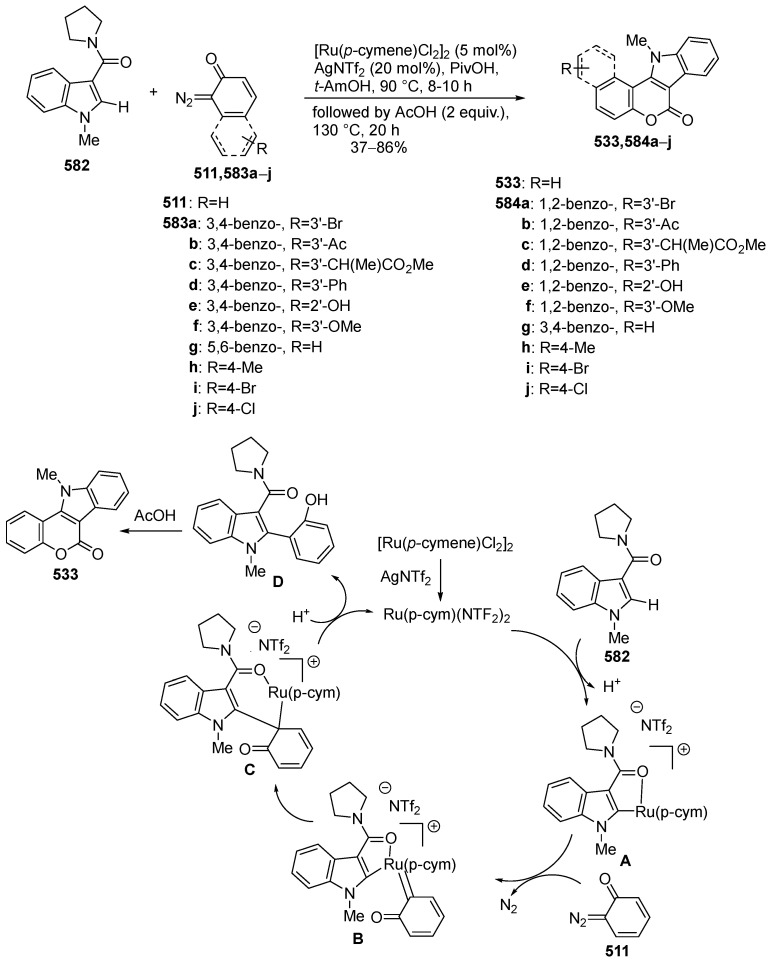
Synthesis of azacoumestans **533 and 584a**–**j**.

Sarkar and Samanda reported along with the synthesis of isolamellarins A **505** and B **507a** the synthesis of azacoumestan **533** and related heterocycles **584a**–**j** under tert-amide-assisted Ru(II)-catalyzed insertion of quinoid carbene in substituted indole-carboxylic acid amides [140]. The Ru-catalyzed reaction of 3-indole amide **597** with o-diazoquinones **511** and **583a**–**j** provided azacoumestan **533** and azacoumestan derivatives **584a**–**j** in 37–86% yields, respectively (Scheme 82). In the proposed mechanism, cationic Ru(II) species were formed by exchange. C-H metalation at C-2 carbon of indole **582** afforded the ruthenacycle **A**. Intermediate **A** reacted with quinonediazide **511** to provide Ru-quinoid intermediate **B**, under removal of nitrogen. Six-membered intermediate **C** was formed by migratory insertion. Protodemetalation of the latter and aromatization furnished intermediate **D**, followed by lactonization in the presence of acetic acid to provide the final product, **533**.

**Scheme 83:**
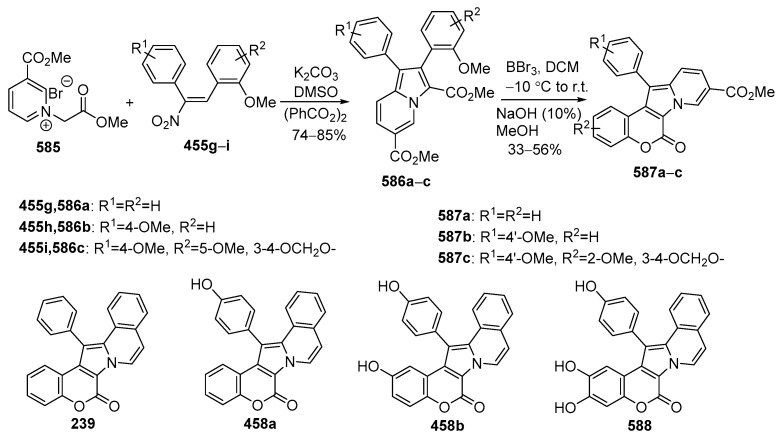
Synthesis of “E-ring free” lamellarin analogues **587a**–**c**.

Recently, Rusanov et al. presented the synthesis and biological evaluation for their cytotoxicity of isoazacoumestan analogues, the “E-ring free” lamellarin analogues **587a**–**c** [155]. The 1,3-dipolar cycloaddition reaction of pyridinium ylide, generated in situ from pyridinium salt **585** under basic conditions, bearing the electron-withdrawing group CO_2_Me in meta-position, with α-stilbenes **455g**–**i** afforded the indolizine derivatives **586a**–**c** in 74–85% yields (Scheme 83). The selective O-demethylation of the o-OMe group with BBr_3_ followed by lactonization resulted in the formation of lamellarin analogues **587a**–**c** in 33–56% yields.

In the evaluation of anticancer activity for compounds **587a**–**c**, they have found that these compounds are devoid of cytotoxicity. In comparison, lamellarin analogues **239**, **458a**, **458b**, and **588**, bearing the “E-ring”, exhibited cytotoxicity in a micromolar concentration range in four cancer cell lines, especially for **458a**, **458b**, and **588**.

**Scheme 84:**
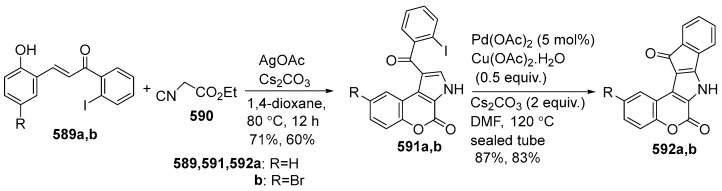
Synthesis of pentacycle isoazacoumestan analogues **592a**,**b**.

Das et al. demonstrated the reactions of 2-hydroxychalcones like **589a**,**b** with ethyl isocyanoacetate **590** for the synthesis of 1-benzoylchromeno[3,4-*b*]pyrrol-4(3*H*)-ones like **591a**,**b** [156]. The [3 + 2] cycloaddition reaction of ethyl isocyanoacetate (**590**) in the presence of a base with the double bond of chalcone **589** followed by intramolecular lactonization afforded pyrrolocoumarin **581** in one step. When the o-iodo substituent existed in the benzoyl group, like **591a**,**b**, the Pd-catalyzed cross-coupling reaction resulted in the formation of pentacycle isoazacoumestan analogues **592a**,**b** in 87% and 83% yields, respectively (Scheme 84).

## 3. Conclusions

In this literature review, three routes were presented for the synthesis of lamellarins. First, pyrrole derivatives are the starting or the intermediate compounds, and then they are fused to isoquinoline or a coumarin moiety. Then, isoquinoline is the starting compound, next forming the indole moiety. In the last route, with fewer synthetic procedures, the synthesis starts from coumarins, which are then fused to the pyrrole moiety and to the isoquinoline framework. The synthesis processes of isolamellarins, azacoumestans, isoazacoumestans, and analogues are also described. Similar reaction strategies are utilized for the construction of pyrrole, isoquinoline, or coumarin rings, such as metal-catalyzed cross-coupling, [3 + 2] cycloaddition, substitution, lactonization, multicomponent reactions, or *N*-ylide-mediated pyrrole ring formation. The following reactions, the Suzuki–Miyaura cross-coupling reaction, Heck reaction, Stille reaction, Sonogashira reaction, Cadogan reaction, Michael addition, Perkin condensation, Friedel–Crafts reaction, Bischler–Napieralski reaction, Vilsmeier–Haack formylation, Paal–Knorr reaction, Baeyer–Villiger oxidation, and Diels–Alder reaction, are useful for the described synthesis.

The title compounds exhibit properties including cytotoxicity, multidrug resistance (MDR), topoisomerase I-targeted antitumor, anti-HIV, antiproliferative, anti-neurodegenerative disease, anti-inflammatory, and antiosteoporotic activities. Especially, lamellarin D (**84**) and its derivatives **464a**,**c**,**d** demonstrated cytotoxic activity against the Hella cell lines in the range of 10.7–70.0 nM with the hydroxy group at the C-8 and C-20 positions to be important for this activity. Lamellarin’s D derivatives with valine (**386c**) and proline (**386d**) have been selected for preclinical trials as they were found to be good antitumor agents targeting topoisomerase I with GI_50_ = 10.8 nM and 18.6 nM, respectively. Lamellarin H (**181**) exhibited the inhibition of HIV-1 integrase with IC_50_ = 1.3 μM and is active against MCV topoisomeraee with IC_50_ = 0.23 μM. In the test against neurodegenerative disease targeting casein kinase 1 (CK1δ), lamellarin A4 (**246**) demonstrated IC_50_ = 3 μM. Azacoumestrol (**529**) inhibited the VEGFR2 kinase in the 10 μM range, blocking the progression of angiogenesis.

We hope this review can benefit researchers in the fields of lamellarins, azacoumestans, and generally the field of coumarins.

## Data Availability

No new data were created or analyzed in this study.
